# A poxvirus ankyrin protein LSDV012 inhibits IFIT1 in a host-species-specific manner by compromising its RNA binding ability

**DOI:** 10.1371/journal.ppat.1012994

**Published:** 2025-03-17

**Authors:** Shijie Xie, Yongxiang Fang, Zhiyi Liao, Lianxin Cui, Kang Niu, Shuning Ren, Junda Zhu, Wenxue Wu, Zhizhong Jing, Chen Peng

**Affiliations:** 1 National Key Laboratory of Veterinary Public Health, College of Veterinary Medicine (CVM), China Agricultural University, Beijing, China; 2 Beijing Key Laboratory for Prevention and Control of Infectious Diseases in Livestock and Poultry, Institute of Animal Husbandry and Veterinary Medicine, Beijing Academy of Agriculture and Forestry Sciences, Beijing, China; 3 State Key Laboratory for Animal Disease Control and Prevention, College of Veterinary Medicine, Lanzhou University, Lanzhou Veterinary Research Institute, Chinese Academy of Agricultural Sciences, Lanzhou, China; Thomas Jefferson University, UNITED STATES OF AMERICA

## Abstract

Poxviruses are large DNA viruses with an arsenal of immune-modulatory genes, many of which remain uncharacterized. Proteins with ankyrin repeats are distinct features of poxviruses, although the biological functions of ankyrin proteins are not fully understood. Lumpy skin disease virus (LSDV) encodes five proteins with ankyrin repeats. Here, we reveal the role of LSDV012, an ankyrin protein, in conferring resistance to type I interferon (IFN) in cells. Deletion of LSDV012 from LSDV significantly impacted viral replication in the presence of type I IFN, highlighting the importance of LSDV012 in antagonizing type I IFN responses. Further investigation revealed that LSDV012 interacted with interferon-induced proteins with tetratricopeptide repeats (IFITs), particularly IFIT1, altering its subcellular localization, interacting with its C-terminus and inhibiting its RNA-binding ability without inducing its degradation. Phylogenetic analysis demonstrated that LSDV012 orthologs are conserved in *capripoxviruses* and *cervidpoxviruses*, and exhibit host species-specific interactions with IFIT1. Notably, LSDV012 was able to rescue the degradation of IFIT1 mediated by VACV C9. These findings provide novel insights into the viral strategies employed by LSDV to subvert host antiviral defenses and underscore the evolutionary adaptations of poxvirus ankyrin proteins in host species-specific immune evasion.

## Author summary

In this study, we investigated the role of ankyrin repeat proteins in the Lumpy skin disease virus (LSDV), focusing specifically on LSDV012. Poxviruses, known for their large genome size (>180kbp), often encode proteins with ankyrin repeats, yet their functions are not fully understood. Our research revealed that LSDV012 plays a crucial role in providing resistance to type I interferon (IFN) in infected cells. By deleting LSDV012 from the virus, we observed a significant reduction in viral replication when type I IFN was present, indicating LSDV012’s role in counteracting the host’s antiviral response. Further analysis showed that LSDV012 interacts with interferon-induced proteins with tetratricopeptide repeats (IFITs), specifically IFIT1, modifying its subcellular localization and inhibiting its RNA-binding capacity without degrading the protein. Phylogenetic studies indicated that LSDV012 orthologs are conserved across capripoxviruses and cervidpoxviruses, highlighting species-specific interactions with IFIT1. Interestingly, LSDV012 also rescued IFIT1 from degradation mediated by vaccinia virus (VACV) C9. These findings enhance our understanding of how LSDV evades host immune defenses and the evolutionary significance of ankyrin proteins in poxviruses.

## 1 Introduction

Poxviruses, representative members of the Nucleocytoplasmic large DNA viruses (NCLDVs) [1], are renowned for encoding a vast array of protein products that modulate or subvert the host’s immune responses. Many poxviruses encode proteins with ankyrin repeat domains (ANK), commonly found in mammals and bacteria [2]. ANKs are generally responsible for protein-protein interactions, facilitating a diverse set of biological functions within cells. The essence of ANK is captured by a tandemly repeated module of approximately 33 amino acids, which fold into a distinct helix-turn-helix structure [2]. In poxviruses, ANKs often modulate host cellular pathways, particularly by subverting immune responses [3–11]. For instance, the vaccinia virus C9, an ANK protein with a C-terminal F-box domain, binds and degrades host IFITs by recruiting cellular SCF (CUL1, SKP-1, F-box) components [12, 13]. Similarly, CPXV007 binds and degrades host RIPK3 through the same recruitment mechanism [14]. However, the functions of the majority of poxvirus ANK proteins remain poorly characterized [3, 4].

Mpox virus (MPXV) [15]and lumpy skin disease virus (LSDV) have garnered significant attention in poxvirus research due to their recent expansion in transmission [16]. LSDV, a member of the Capripoxvirus genus, presents an emerging challenge to agriculture and veterinary medicine [17]. The consequences of LSDV infection include infertility, reduced milk production, and occasional mortality among affected herds, posing a substantial economic threat to the cattle and water buffalo industries [18–21]. Outbreaks of LSD have continued to increase, gradually expanding from sub-Saharan Africa to countries in Europe, and Eastern and Southeast Asia, underscoring the importance of extensive research in LSDV virology and the implementation of more effective biosecurity measures [22]. The genome of LSDV encodes five proteins with ANK: LSDV012, LSDV145, LSDV147, LSDV148, and LSDV152 [23]. The biological function has not yet been reported.

Interferon-induced proteins with tetratricopeptide repeats (IFITs) are a class of antiviral proteins, comprised of 4 members, ISG56/IFIT1 [24, 25], ISG54/IFIT 2[26], ISG60/IFIT3 [24,27], and ISG58/IFIT5 [28, 29] that are strongly induced in response to type I interferon (IFN) stimulation [25,30,31]. IFITs have a wide range of antiviral functions, especially for viruses that do not have a cap at the 5’ end, and the inhibition effect is significant [32]. Three different modes of action have been reported for IFITs [33,34]: 1) IFIT1/5 can directly bind to viral RNA and inhibit viral protein translation. 2) IFIT1 can bind to host proteins or viral proteins and inhibit their normal function. 3) IFITs participate in antiviral immune signal transduction and activate or inhibit the synthesis of other antiviral proteins.

Here, we aimed to elucidate the biological function of LSDV012, a previously uncharacterized ANK protein. An IP-Mass spec analysis indicated that LSDV012 interacted with bovine IFIT1, and further molecular characterization suggested that LSDV012 effectively inhibited the function of IFIT1 without causing its degradation. Mechanistically, LSDV012 is competitively bound to IFIT1, thereby preventing its interaction with mRNA, a step crucial for its antiviral function. Subsequently, we conducted a phylogenetic analysis of 636 poxvirus ankyrin (ANK) proteins spanning various poxvirus genera and identified several LSDV012 orthologs from *chordopoxvirinae* and *entomopoxvirinae*. All identified ANK proteins were further categorized into 35 distinct groups, with LSDV012 assigned to group ANK4. Importantly, orthologs of LSDV012 were not only found in *capripoxviruses* but also deerpox virus, tanapox virus, and eptesipox virus. Interestingly, we demonstrated that LSDV012 exhibited host species-specific interaction with IFIT1 from different species. These findings underscore the importance of adaptation in the co-evolution of viruses with their host species and highlight why host species are a crucial factor in researching the interplay between viruses and antiviral immune responses. Our findings unveil a novel mode of action by which IFIT1 is inhibited by a viral antagonist and emphasize the importance of continuous research into poxvirus ANK proteins.

## 2 Results

### 2.1 LSDV012 impacts viral resistance to type I IFN

**Fig 1:**
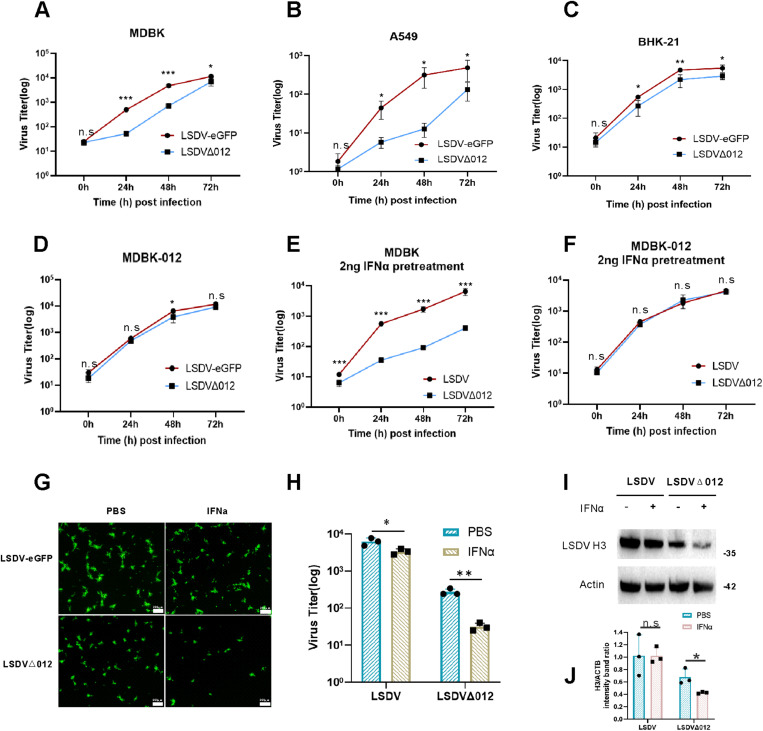
LSDV Δ**012 demonstrates heightened sensitivity to IFN**α **compared to LSDV.** Growth curves were measured in various cell lines following infection with LSDV or LSDVΔ012 at a multiplicity of infection (MOI) of 0.01 pfu/cell. Viral titers were determined at 0, 24, 48, and 72 hours post-infection in the following cell types: MDBK cells (A), A549 cells (B), BHK-21 cells (C), and MDBK-012 cells(D). Effect of Interferon on LSDVΔ012 Replication. MDBK cells (E) and MDBK-012 cells (F) were pretreated with different concentrations of IFNα (2ng/ml) for 12 hours. Cells were then infected with either LSDV or LSDVΔ012 at 0.01 pfu/cell, and viral titers were measured at 0, 24, 48, and 72 hours post-infection. Additionally, MDBK cells were pre-stimulated with PBS or IFNα (2ng/ml) for 12 hours, followed by infection with LSDV-eGFP or LSDVΔ012 virus at 0.01 pfu/cell for 48 hours. Fluorescence images were captured (I), and viral titers (J), along with LSDV H3 protein levels (K and L), were evaluated from collected samples. Significance Levels: *p < 0.05, ** p < 0.01, *** p < 0.001, n.s: non-significant.

To determine if LSDV012 contributes to LSDV’s resistance to the treatment of type I IFN, a recombinant virus was constructed by replacing the open reading frame (ORF) of LSDV012 with a cassette containing an eGFP driven by a poxvirus P11 promoter. The resulting virus was clonally purified, confirmed by Sanger sequencing, and named LSDVΔ012 (S1A and S1B Fig). In addition, a recombinant LSDV expressing a P11-driven eGFP was included as a control [22]. MDBK, A549, and BHK21 cells were infected with LSDV-eGFP or LSDVΔ012 at 0.01 pfu/cell, viruses were harvested at 0, 24, 48, and 72 h post-infection (hpi) and a plaque assay determined viral titers reported previously and growth curves were generated [22]. Deletion of LSDV012 significantly compromised the replication of LSDV in MDBK and A549 cells but not in BHK21 cells, in which the type I IFN responses are naturally deficient [35] (Fig 1 A-C). To confirm whether LSDV infection activates type I IFNs in cells, real-time PCR was employed to detect the level of IFNα/β in cells after LSDV infection (S1D Fig). LSDV infection caused the up-regulation of IFNα/β expression in A549 and MDBK cells, but not in BHK-21 cells. In addition, we found that type I IFN exhibited a dose-dependent effect on LSDV replication (S1C Fig). To verify whether the reduced replication could be attributed to type I IFN, an MDBK cell line that constitutively expresses a codon-optimized version of LSDV012 was generated. The ectopically expressed LSDV012 contained a 2× myc tag at the N-terminus (S1E Fig) named MDBK-012 cell line. We selected 2 ng/ml IFNα as the optimal concentration due to its relatively mild inhibitory effect on LSDV replication compared to higher concentrations (S1C Fig). Despite this, it is adequate to trigger activation of the interferon signaling pathway, as evidenced by a significant increase in ISG15 mRNA expression (S1G Fig). MDBK and MDBK-012 cells were pre-treated with IFNα for 12 h before LSDV infection and the results revealed that IFNα treatment dramatically depressed the replication of LSDVΔ012 in comparison to LSDV-eGFP in MDBK cells (Fig 1E), but there was no significant difference in MDBK-012 cells (Fig 1F). At this concentration, however, the inhibitory effect on LSDVΔ012 virus infection at a high multiplicity of infection (MOI) was not pronounced (S1H-J Fig). In contrast, viral replication at a low MOI was significantly suppressed (Fig 1G-J). Viral replication and post-replicative protein synthesis (H3) were also monitored by observing eGFP signals (Figs 1H and S1G) and Western blotting analysis (Fig 1I and 1J), respectively, and IFNα treatment severely impacted the protein synthesis (H3) of LSDVΔ012. It is worth noting that the treatment of IFNα also slightly reduced the replication and protein synthesis of LSDV, although the difference was much less prominent than that of LSDVΔ012. In summary, these data indicated that LSDV012 protein may antagonize the antiviral status induced by IFNα in cells.

### 2.2 LSDV antagonizes type I IFN response by targeting IFITs.

**Fig 2:**
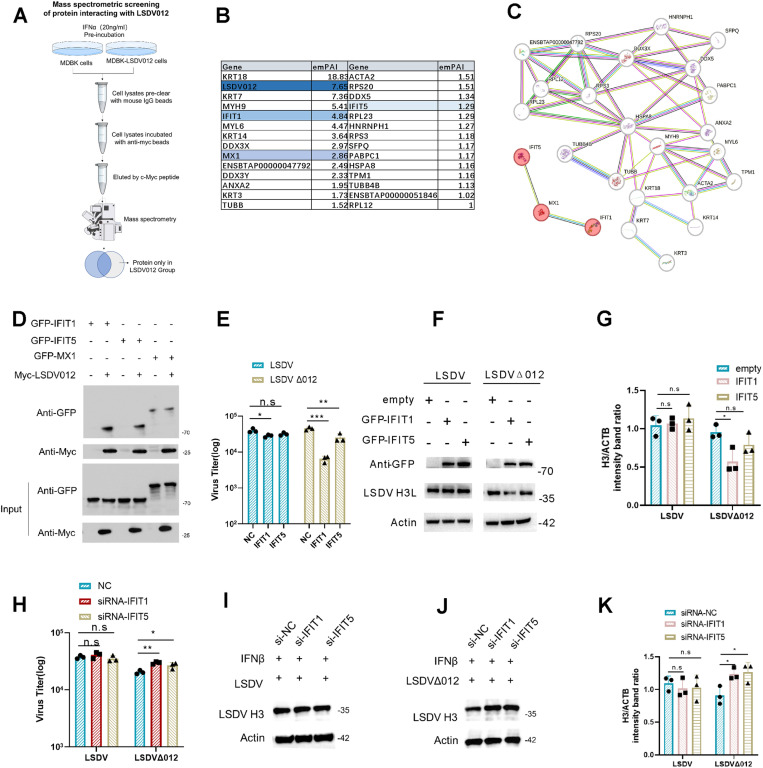
IFIT1/5 identified as proteins binding to LSDV012. Flowchart for LSDV012 co-immunoprecipitation and protein identification, sourced from SciDraw (https://scidraw.io/), a free repository of scientific illustrations (A). A total of 30 candidate proteins potentially interacting with LSDV012 were identified through mass spectrometry proteomics analysis (B). A schematic diagram displaying the candidate proteins involved in the interferon (IFN) signaling pathway was generated using the STRING database (C). To confirm the proteomics data, BHK-21 cells were co-transfected with IFIT1/5 or MX1 alongside LSDV012 for 24h. Co-immunoprecipitation was performed using myc magnetic beads (D). Impact of IFIT1/5 Overexpression on LSDV/LSDVΔ012 Replication. BHK-21 cells were transfected with IFIT1 or IFIT5, followed by infection with LSDV or LSDVΔ012 at an MOI of 0.01. After 48 hours, samples were collected to assess viral titers (E) and measure viral protein levels (F and G). Effect of IFIT1/5 Knockdown on LSDV/LSDVΔ012 Replication. A549 cells were transfected with siRNA targeting IFIT1 or IFIT5 for 12 hours, followed by treatment with IFNβ (5 ng/ml) for another 12 hours. The cells were then infected with LSDV or LSDVΔ012 at 0.1 pfu/cell for 48 hours, after which viral titers (H) and the levels of LSDV H3 protein (I-K) were measured. Significance Levels: *p < 0.05, ** p < 0.01, *** p < 0.001, n.s: non-significant.

To identify the cellular target of LSDV012, MDBK-012 cells, and the control MDBK cells were treated with IFNα (20 ng/ml) for 24h, and then total protein was harvested and subjected to immunoprecipitation using magnetic myc-beads. Proteins pulled down were then analyzed with mass spectrometry (illustrated in Fig 2A). A total of 30 candidate proteins (Fig 2B and S4 Table) were identified in the MDBK-LSDV012 group and a string analysis indicated that MX1, IFIT1, and IFIT5 were associated with the type I IFN signaling pathway (Fig 2C). The interactions between LSDV012 and MX1, IFIT1, or IFIT5 were verified via co-IP analysis in which cells were co-transfected with LSDV012 and the candidate interacting partners, and a positive association between LSDV012 and IFIT1 as well as IFIT5 was confirmed (Fig 2D). To validate that IFITs were the main factors that suppressed the replication of LSDVΔ012, bovine IFIT1 or IFIT5 were transfected into BHK-21 cells followed by infection with LSDV-eGFP or LSDVΔ012, and viral titers were determined by plaque assay described above. Ectopic expression of IFIT1 or IFIT5 did not exhibit any observable effect on the replication of LSDV-eGFP but significantly restrained the replication of LSDVΔ012 (Fig 2E), indicating IFIT1 was the primary target of LSDV012. Similarly, the synthesis of H3, a viral intermediate/late protein [36], was monitored in cells transfected with IFIT1 or IFIT5 and IFIT1 displayed a discernable inhibition on H3 synthesis in only LSDVΔ012-infected cells, but not in LSDV-eGFP-infected cells (Fig 2F and 2G). In addition, siRNA targeting IFIT1/5 (S2E Fig) was able to partially increase the replication of LSDVΔ012 (Fig 2H-K). A confocal microscopic analysis was then employed to analyze the subcellular localization of LSDV012 and IFIT1. Interestingly, while IFIT1 exhibited a diffuse pattern in the cytoplasm, co-transfection of LSDV012 dramatically altered the distribution of IFIT1, redirecting it to form a distinct perinuclear ring structure (S2A Fig). Taken together, these data indicated LSDV012 was associated with bovine IFIT1 and altered its subcellular localization.

### 2.3 LSDV012 prohibits the degradation of IFIT1 mediated by VACV C9L.

**Fig 3:**
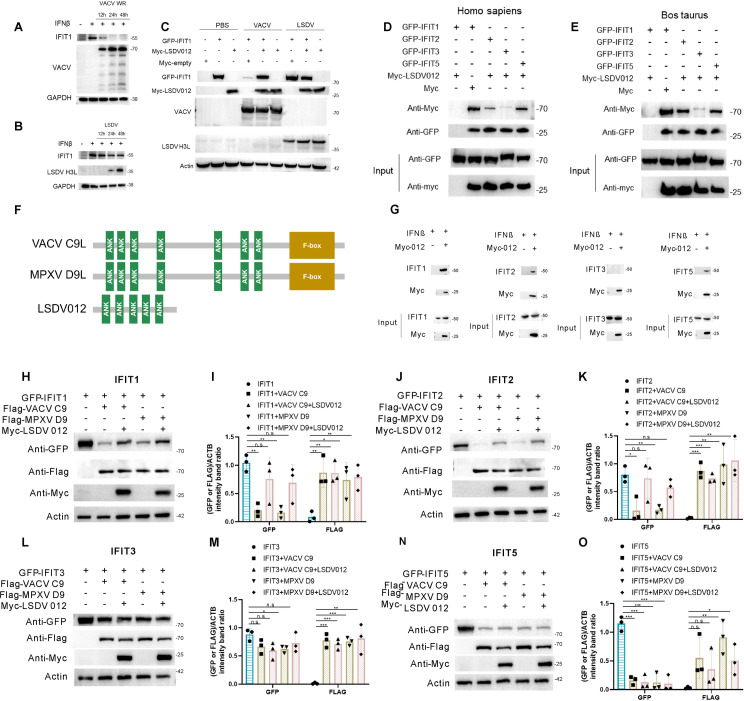
LSDV012 inhibits the degradation of IFIT1/2 by VACV C9L. Effect of VACV and LSDV Infections on IFIT1 Expression in Cells. A549 cells were pretreated with IFNβ (5 ng/ml) for 24 hours. The expression levels of IFIT1 were then measured 12, 24, and 48 hours post-infection with either VACV WR (A) or LSDV (B) at a multiplicity of infection (MOI) of 0.1 pfu/ml. Impact of LSDV012 on VACV-Mediated IFIT1 Degradation. BHK-21 cells were transfected with IFIT1 and/or LSDV012 for 24 hours, followed by infection with PBS, VACV WR, or LSDV for an additional 24 hours. GFP-IFIT1 and myc-LSDV012 were detected using GFP and Myc antibodies, respectively (C). Interaction Between LSDV012 and IFIT1/2/5 from Different Species. BHK-21 cells were transfected with human IFIT1/2/3/5 (D) or bovine IFIT1/2/3/5 (E), alongside LSDV012 for 24 hours. Co-immunoprecipitation was then performed using myc magnetic beads to detect interactions. Interaction Between LSDV012 and Endogenous IFIT1/2/5. A549 cells were transfected with myc-LSDV012. Four hours later, the medium was replaced with an IFNβ-containing culture medium, and cells were incubated for an additional 24 hours. Co-immunoprecipitation was conducted using myc beads (G). A schematic diagram illustrates the protein domains of LSDV012, VACV C9, and MPXV D9 (F). Inhibition of VACV C9 and MPXV D9-Mediated Degradation of IFIT Proteins by LSDV012. BHK-21 cells were co-transfected with myc-LSDV012, flag-VACV C9 or flag-MPXV D9, and human GFP-IFIT1 (H and I), GFP-IFIT2 (J and K), GFP-IFIT3 (L and M), or GFP-IFIT5 (N and O) for 24 hours. Western blotting was performed using GFP, Myc, and Flag antibodies to assess the impact of LSDV012 on the stability of IFIT1/2/3/5 in the presence of VACV C9 and MPXV D9. Significance Levels: p < 0.05, ** p < 0.01, *** p < 0.001, n.s: non-significant.

A previous report illustrated that VACV ANK protein C9 interacts with human IFIT1 recruits the Skp1-CUL1-F-box (SCF) components, and targets IFIT1 for proteasomal degradation [12,37]. To ascertain whether LSDV012 functions similarly, A549 cells pre-treated with human IFNβ were infected with VACV-WR or LSDV, and the cellular levels of IFIT1 were monitored via Western blotting analysis. In agreement with the referenced study, VACV-WR infection led to evident IFIT1 degradation (Figs 3A and S2B). Nevertheless, infection of LSDV did not result in the decrease of IFIT1 (Fig 3B and S2C). To determine if LSDV012 affected C9-mediated IFIT1 degradation, BHK-21 cells were co-transfected with bovine IFIT1, LSDV012 for 24 h, and then mock-infected or infected with VACV-WR, or LSDV at 0.1 pfu/cell. Total protein was harvested and the levels of IFIT1 were monitored by Western blotting analysis. As shown in Fig 3C, although VACV led to a dramatic decrease of IFIT1 as a result of C9-mediated degradation (Fig 3C lane 4), co-transfection of LSDV012 rescued the loss of IFIT1 (Fig 3C lane 5). In comparison, LSDV infection did not cause any detectable IFIT1 degradation (Fig 3C lane 7). In addition, LSDV012 exhibited a dose-dependent rescue of the degradation of IFIT1 mediated by VACV C9 (S2D Fig). These results indicated that LSDV012 may prevent the interaction between C9 and IFIT1 and inhibit the degradation of the latter. To verify the association between LSDV012 and IFITs, a co-IP analysis was conducted by pulling down with antibodies specific to myc-tagged LSDV012 in cells ectopically expressing human or bovine IFITs. LSDV012 was able to pull down both human (Fig 3D) and bovine (Fig 3E) IFIT1, IFIT2, and IFIT5, while much less IFIT3 was pulled down by LSDV012. In A549 cells, LSDV012 was also able to pull down endogenous IFITs (Fig 3G).

As LSDV infection is restricted to cattle and buffalo, we were surprised to observe its association with human IFITs and its capacity to inhibit C9-mediated IFIT degradation. To further elucidate this phenomenon, we examined the effect of LSDV012 on C9-promoted human IFITs (IFIT1, 2, 3, and 5) degradation and included the ortholog of C9 from MPXV, which exhibits 90.9% sequence identity to VACV C9 but only 14.9% to LSDV012. Both VACV C9 and MPXV D9 contained an F-box domain at their C-terminus, which is absent in LSDV012 (Fig 3F). Both VACV C9 and MPXV D9 were able to promote IFIT1 degradation, which was rescued by the presence of LSDV012 (Fig 3H and 3I). Similarly, IFIT2 was sensitive to both VACV C9 and MPXV D9-mediated degradation, which were ameliorated by LSDV012 (Fig 3J and 3K). In comparison, IFIT3 was not degraded by VACV C9 or MPXV D9 (Fig 3L and 3M). Interestingly and remarkably, IFIT5 was sensitive to both C9 and D9-mediated degradation, but LSDV012 failed to rescue this degradation (Fig 3N and 3O). Taken together, these data demonstrated that LSDV012 was able to bind both human and bovine IFIT1/2/5, and was able to revert the degradation of human IFIT1/2 promoted by VACV C9 or MPXV D9.

### 2.4 LSDV012 orthologs are conserved in only *capripoxviruses* and *cervidpoxviruses.*

**Fig 4:**
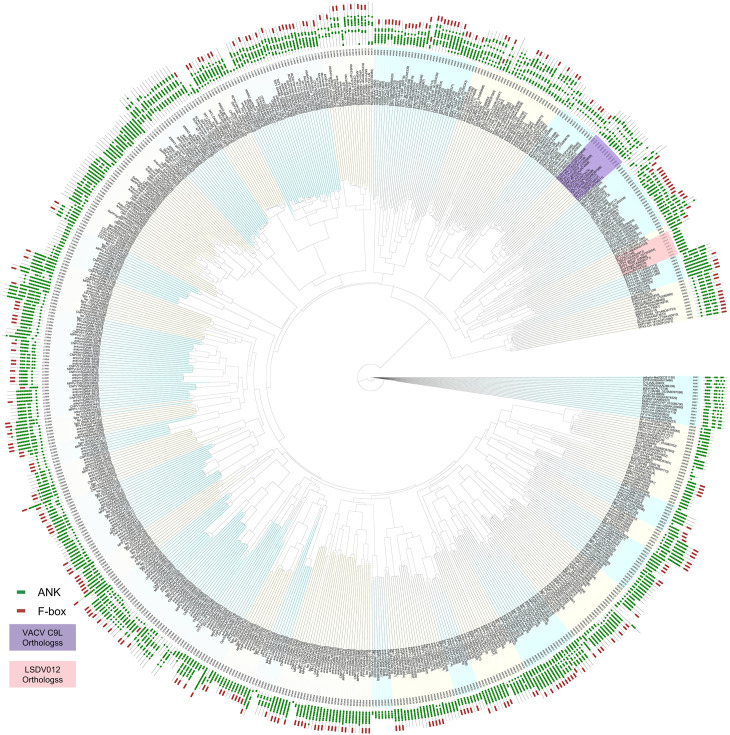
Phylogenetic analysis of 636 ankyrin from poxvirus.

As both LSDV012 and VACV C9 can bind IFIT1 and are both characterized as poxvirus ANK proteins, we performed a phylogenetic analysis of poxvirus ANK proteins and assessed their evolutionary correlation. In addition, by using the HMM model ANK_2 (PF12796) from the Pfam database, we re-identified proteins with ANK from 63 reference strains (Table 1 and S3A Fig) of various poxviruses, including 55 strains of *chordopoxvirinae* and 8 strains of *entomopoxvirinae*. A total of 636 proteins were identified to contain ANK and are thus considered ANK proteins (Fig 4 and S1 Table). Based on evolutionary relationships and protein domains, we further categorized all 636 proteins into 35 groups, designated as ANK1 to ANK35 (Fig 4 and S1 Table). VACV C9 (VACWR019) and MPXV D9 both belong to group ANK6, which includes ANK proteins primarily conserved in *orthopoxviruses* (S2 Table) and those found in Cotia virus (COTV023) and NY_014 poxvirus (NY_014-196). LSDV012, on the other hand, was categorized into group ANK4 (Table 2), which includes members conserved in SPPV, GTPV, and Deerpox virus. Canarypox virus possesses 51 ANK proteins, the highest number among all viruses examined, constituting a notable 15.54% (51 out of 328) of its total protein inventory. In contrast, members of the *entomopoxvirinae* do not contain any ANK. Similarly, several *chordopoxvriuses* (Saltwater crocodilepox virus, Nile crocodilepox virus, Western kangaroopox virus, Eastern grey kangaroopox virus, Molluscum contagiosum virus, Equine molluscum contagiosum-like virus, Salmon gill poxvirus, Squirrelpox virus) also lack ANK proteins.

**Table 1:** The count of ANK proteins across various poxviruses.

Poxviridae	Genus	Species	Strain	GenBank ID	Number
Chordopoxvirinae	Avipoxvirus	Canarypox virus	ATCC VR-111	AY318871.1	51
		Penguinpox virus	YEP-NZ	MW296038.1	48
		Magpiepox virus	62-11-06-2000-ANU	MW485973.1	40
		Flamingopox virus	FGPVKD09	MF678796.1	36
		Fowlpox virus		AF198100.1	31
		Pigeonpox virus	FeP2	KJ801920.1	26
		Turkeypox virus	TKPV-HU1124/2011	KP728110.2	15
		Albatrosspox virus	SAN97-0665NZ	MW365933.1	48
		Mudlarkpox virus	MLPV-AU2019	MT978051.1	46
	Capripoxvirus	Goatpox virus	Pellor	NC_004003.1	5
		Sheeppox virus	17077-99	NC_004002.1	5
		Lumpy skin disease virus	NI-2490	AF325528.1	5
	Centapoxvirus	NY_014 poxvirus	2013	MF001305.1	11
		Murmansk poxvirus	LEIV-11411	MF001304.1	9
		Yokapox virus	DakArB 4268	HQ849551.1	7
	Cervidpoxvirus	Moosepox virus GoldyGopher4	GoldyGopher14	MG751778.1	6
		White-tailed deer poxvirus	OV179	MF966153.1	6
		Deerpox virus	W-848-83	AY689436.1	6
	Crocodylidpoxvirus	Saltwater crocodilepox virus	J1	MK903858.1	0
		Nile crocodilepox virus		DQ356948.1	0
	Leporipoxvirus	Myxoma virus	Lausanne	AF170726.2	5
		Rabbit fibroma virus	Kasza	AF170722.1	4
	Macropopoxvirus	Western kangaroopox virus	Western Australia	MF467280.1	0
		Eastern grey kangaroopox virus	Sunshine Coast	MF467281.1	0
	Molluscipoxvirus	Molluscum contagiosum virus		U60315.1	0
		Equine molluscum contagiosum-like virus	Tanzania/2016	MN339351.1	0
	Mustelpoxvirus	Sea otter poxvirus	ELK	MH427217.1	1
	Orthopoxvirus	Akhmeta virus	Akhmeta_2013-88	MH607141.1	16
		Cowpox virus	Brighton Red	AF482758.2	16
		Raccoonpox virus		KP143769.1	15
		Abatino macacapox virus	Abatino	NC_055231.1	14
		Skunkpox virus	WA	KU749310.1	14
		Volepox virus	CA	KU749311.1	13
		Taterapox virus	Dahomey 1968	DQ437594.1	11
		Vaccinia virus	WR	AY243312.1	10
		Monkeypox virus	Zaire-96-I-16	AF380138.1	10
		Ectromelia virus	Moscow	AF012825.2	8
		Camelpox virus	M-96	AF438165.1	8
		Variola virus	India-1967	X69198.1	7
		Alaskapox virus	Alaska_2015	MN240300.1	13
	Oryzopoxvirus	Cotia virus	SPAn232	NC_016924.1	11
		BeAn 58058 virus	BeAn58058	KY094066.1	8
	parapoxvirus	Parapoxvirus red deer/HL953	HL953	KM502564.1	7
		Bovine papular stomatitis virus	BV-AR02	AY386265.1	7
		Pseudocowpox virus	VR634	GQ329670.1	7
		Orf virus	OV-SA00	AY386264.1	5
		Grey sealpox virus	AFK76s1	KY382358.2	4
		Equine parapoxvirus	EqPPV/A1	OR544017.1	1
	Pteropopoxvirus	Pteropox virus	Australia	KU980965.1	1
	Salmonpoxvirus	Salmon gill poxvirus		KT159937.1	0
	Sciuripoxvirus	Squirrelpox virus	Red squirrel UK	HE601899.1	0
	Suipoxvirus	Swinepox virus	17077-99	AF410153.1	4
	Vespertilionpoxvirus	Eptesipox virus	Washington	KY747497.1	7
	Yatapoxvirus	Tanapox virus		AJ293568.1	5
		Yaba monkey tumor virus		AY386371.1	3
Entomopoxvirinae	Alphaentomopoxvirus	Anomala cuprea entomopoxvirus	CV6M	AP013055.1	0
	Betaentomopoxvirus	Mythimna separata entomopoxvirus		HF679134.1	0
		Choristoneura biennis entomopoxvirus		HF679132.1	0
		Adoxophyes honmai entomopoxvirus		HF679131.1	0
		Choristoneura rosaceana entomopoxvirus		HF679133.1	0
		Amsacta moorei entomopoxvirus		AF250284.1	0
	Deltaentomopoxvirus	Melanoplus sanguinipes entomopoxvirus	Tucson	AF063866.1	0
	Entomopoxvirinae	Linepithema humile entomopoxvirus	11CAT08	MH213250.1	0

**Table 2:** The binding capacity of LSDV012 orthologs to IFIT1 proteins from various species.

Genus	Species	Gene	GenBank ID	Group	Natural host	IFIT1		
						Bos taurus	Homo sapiens	Horseshoe bat
Cervidpoxvirus	Moosepox virus GoldyGopher4	MPV_014	AYC44724	ANK4	Moose	+	–	–
	White-tailed deer poxvirus	DPV014	AUI80576	ANK4	White-tailed deer	+	–	–
	Deerpox virus	DpV83gp014	ABI99171	ANK4	mule deer	+	–	–
Capripoxvirus	Goatpox virus	GTPV_gp010	YP_001293204	ANK4	Goat	+	+	–
	Sheeppox virus	SPPV_010	NP_659586	ANK4	Sheep	+	+	–
	Lumpy skin disease virus	LSDV012	AAK84973	ANK4	cattle	+	+	–
Yatapoxvirus	Tanapox virus	8L	CAC21246	ANK4	primates	–	+	+
Vespertilionpoxvirus	Eptesipox virus	EPTV_WA-169	ASK51370	ANK4	Bat	–	–	–

+: Interacting pairs; -: Non-interacting pairs.

Phylogenetic trees were based on 636 ANK proteins from poxvirus with model JTT+F+R8. Bootstrapping was performed with 1,000 replicates. Protein domain data was obtained from the SMART database.

### 2.5 LSDV012 and its orthologs interact with IFIT1 in a host species-specific manner

**Fig 5:**
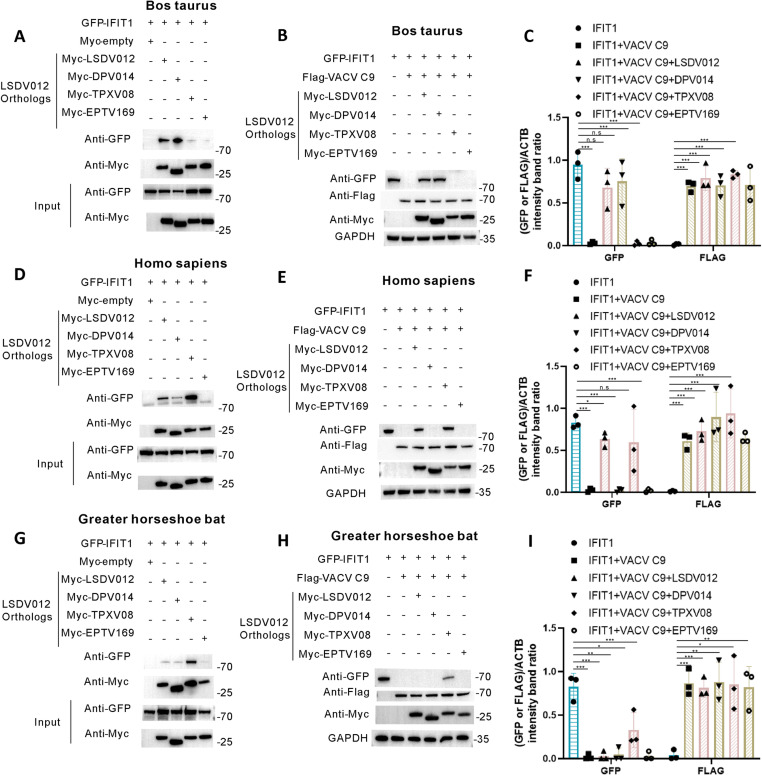
The interaction of LSDV012 orthologs with IFIT1 shows host specificity. Analysis of Binding Ability Between LSDV012 Orthologs and IFIT1 from Different Species. LSDV012 orthologs (myc-LSDV012, myc-DPV014, myc-TPXV08, myc-EPTV169) were co-transfected into 293T cells with bovine GFP-IFIT1 (A), human GFP-IFIT1 (D), and bat GFP-IFIT1 (G) for 24 hours. Following the incubation, co-immunoprecipitation was performed using Myc magnetic beads to assess the binding interactions. To evaluate the ability of LSDV012 orthologs to inhibit the degradation of IFIT1 by VACV C9 across different species, 293T cells were co-transfected with LSDV012 orthologs and Flag-VACV C9, along with bovine GFP-IFIT1 (B and C), human GFP-IFIT1 (E and F), and bat GFP-IFIT1 (H and I) for 24 hours. Western blot analysis was conducted using GFP, FLAG, and Myc antibodies. Significance Levels: p < 0.05, ** p < 0.01, *** p < 0.001, n.s: non-significant.

To further characterize the interaction between LSDV012, and its orthologs identified in the phylogenetic analysis with IFIT1, we cloned LSDV012 orthologs from white-tailed deer poxvirus (DPV014), tanapox virus (TANV 08), and eptesipox virus (EPTV169) and analyzed their interactions with IFIT1 from various host species, including those from the natural host of the above viruses (Table 2). LSDV and deerpox virus typically infect ungulates [38]. Both LSDV012 and DPV014 were able to pull down bovine IFIT1, while TPXV 08 and EPTV 169 exhibited a lower ability to pull down bovine IFIT1 (Fig 5A). The capability of LSDV012 orthologs to pull down IFIT1 also contributed to their ability to rescue C9-mediated IFIT1 degradation, as only LSDV012 and DPV014 were able to avert the degradation of bovine IFIT1 resulted from co-transfection of VACV C9 (Fig 5B and 5C). Tanapox virus (TPXV) is known to infect primates including humans [39]. Although LSDV012, DPV014, and TPXV 08 could all bind to human IFIT1 (Fig 5D), TPXV 08 was able to pull down the highest amount of human IFIT1, which correlates well with TPXV’s host specificity to infect mainly primates. In addition, both LSDV012 and TPXV08 were able to prohibit C9-mediated degradation of human IFIT1 (Fig 5E and 5F). Eptesipox virus (EPTV) is a newly discovered poxvirus isolated from bats [40, 41]. It remains uncertain whether the big brown bat serves as the reservoir species for the virus or if other host species are also involved. Our results revealed that EPTV169 does not interact with IFIT1 in any of the three species mentioned above, including bats (Fig 5). In contrast, TANV08 was able to bind to bat IFIT1 (Fig 5G) and inhibited its degradation by VACV C9 (Fig 5H and 5I). These data collectively demonstrated that LSDV012 and its orthologs from ANK4 exhibited varying abilities to interact with IFIT1 proteins from different host species. Furthermore, some poxvirus LSDV012 orthologs have evolved to interact with IFIT1 from their natural hosts with the greatest efficacy (Table 2).

### 2.6 LSDV012 inhibits IFIT1 by compromising its pppRNA-binding ability.

**Fig 6:**
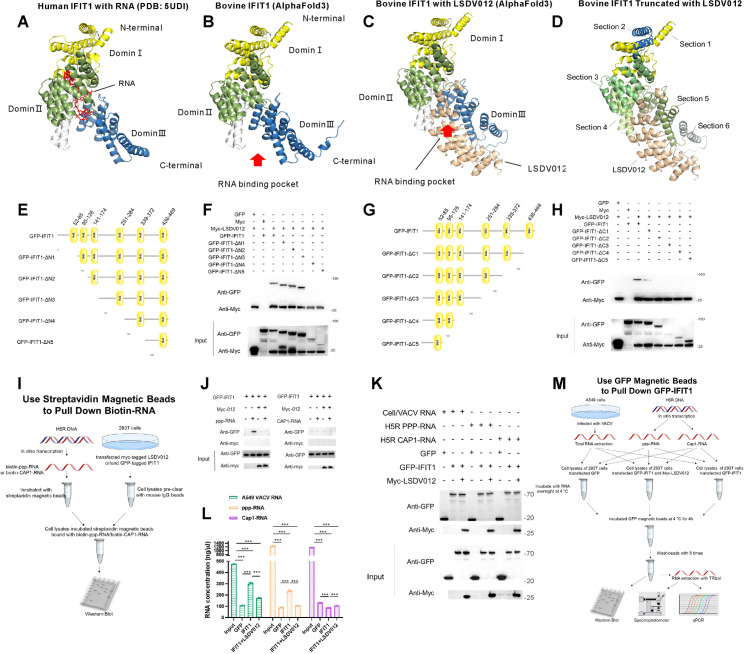
LSDV012 binds to the C-terminus of IFIT1, impeding its mRNA-binding capability. The structure of the human IFIT1 protein complex with RNA (PDB:5UDI) is shown in (A). The predicted structure of bovine IFIT1 by Alphafold3 is illustrated in (B), and the Alphafold3-predicted protein complex structure of IFIT1 with LSDV012 is depicted in (C). The interaction regions between LSDV012 and IFIT1 are segmented into section 1 (1–95 aa), section 2 (96–140 aa), section 3 (141–250 aa), section 4 (151–338 aa), section 5 (339–435 aa), and section 6 (436–478 aa) (D). Schematic representations of the N-terminal truncation (E) and C-terminal truncation (G) of Bovine IFIT1 are presented. LSDV012 validation of binding to different IFIT1 truncations. Myc-LSDV012 was co-transfected with the N-terminal truncation (F) and C-terminal truncation (H) of bovine IFIT1 into 293T cells for 24 hours, followed by co-immunoprecipitation using myc beads. A flow chart illustrating the use of Streptavidin Magnetic Beads to pull down Biotin-labeled RNA was sourced from SciDraw (J). Biotin-ppp-RNA and Biotin-Cap1-RNA (approximately 20 µg) were incubated with Streptavidin Magnetic Beads for 2 hours to prepare the RNA-bead complex. This mixture was then incubated with cell lysates from 293T cells, which were transfected with GFP-IFIT1 alone or in combination with LSDV012 for 24 hours. The incubation was conducted at 4°C for 4 hours. The protein content was analyzed using GFP and Myc antibodies. H5R ppp-RNA, Cap1-RNA, and A549/VACV RNA were incubated with 293T cell lysates at 4°C for 12 hours, which were transfected with GFP/GFP-IFIT1 alone or in combination with LSDV012 for 24 hours. GFP magnetic beads were then added for a 2-hour incubation. These GFP magnetic beads were used to pull down IFIT1, which may be bound to RNA. The protein content was detected using GFP and Myc antibodies (K). RNA was extracted using Trizol, and the concentration analysis was conducted using a spectrophotometer (L). Significant differences: * p < 0.05, ** p < 0.01, *** p < 0.001, n.s: non-significant.

Unlike VACV C9, LSDV012 did not result in the degradation of IFIT1, we next aim to investigate the mechanism by which LSDV012 inhibits IFIT1. Alphafold3 was employed to predict the structure of LSDV012/bovine IFIT1 complex in the presence of RNA using the reported structure of human IFIT1/RNA as the reference [31,42]. Similar to the protein structure of VACV K1 [11], the prediction indicated that LSDV012 comprises five intact ANK structures (A1-A5) and two partial ANK structures—one at the N-terminus and one at the C-terminus (S3B Fig). Each ANK motif predominantly consists of two α-helices, arranged in a repetitive helix-loop-helix-loop configuration. The α-helices are oriented in a reverse parallel manner, culminating in a tightly packed helical arrangement [2]. Furthermore, our structural predictions revealed that bovine IFIT1 closely resembles human IFIT1 (Fig 6A), featuring 23 α-helices, with 18 of these α-helices forming nine TPR motifs (Fig 6B). These motifs collectively organize into three subdomains, creating a single-stranded RNA (ssRNA) binding pocket capable of accommodating five nucleotides (cap + four RNA nucleotides) [31]. Importantly, in the predicted structure, LSDV012 interacted with domain III and positioned to shield the RNA binding pocket of IFIT1 (Fig 6C and S3 Table). We therefore hypothesized that LSDV012 may jeopardize IFIT1’s RNA binding capacity. To confirm this hypothesis, a series of truncated IFIT1 mutants were constructed by removing one or multiple tetratricopeptide repeat (TPR) domains predicted by the Protein SMART database [43]. Co-IP analysis was performed to evaluate the association between the IFIT1 mutants and LSDV012. Removal of the TPRs from the N-terminus of IFIT1 did not compromise its interaction with LSDV012 (Fig 6E and 6F). However, the removal of the C-terminal TPRs (the end predicted to interact with LSDV012) greatly crippled its interaction with IFIT1 (Fig 6G and 6H). These results agreed with the predicted structure, in which the C-terminus of IFIT1 is directly associated with LSDV012 (Fig 6D).

Next, to analyze if LSDV012 was able to inhibit IFIT1’s RNA binding ability. We employed two assays to assess IFIT1’s RNA binding capacity in the presence and absence of LSDV012 (Fig 6I and 6M). Given IFIT1’s non-sequence-specific RNA binding nature [31], we extracted total RNA from VACV-infected A549 cells (containing viral RNA) for experiments and performed *in vitro* transcription of VACV H5R to obtain H5 ppp-RNA and H5 Cap1-RNA (as shown in S3C and S3D Fig). Additionally, UTP in the transcription reaction was replaced with Biotin-16-UTP to produce H5 mRNA labeled with biotin (biotin-ppp-RNA and biotin-Cap1-RNA) [44]. These biotin-labeled RNAs were then incubated with cell lysates from 293T cells transfected with IFIT1, with or without LSDV012. Magnetic streptavidin beads were used to pull down the biotin RNA-protein complexes for subsequent Western blot analysis (Fig 6I). The presence of ppp-RNA was able to pull down IFIT1, indicating the interaction between IFIT1 and RNA was detectable by this method. Notably, in cells co-transfected with LSDV012, the association between IFIT1 and ppp-RNA was no longer observed (Fig 6J). Furthermore, the interaction between IFIT1 and ppp-RNA depends on the triphosphate structure, as Cap1-labeled RNA did not pull down IFIT1 (Fig 6J, right panel).

Next, we incubated ppp-RNA, Cap1-RNA, and A549-derived VACV mRNA with cell lysates from 293T cells transfected with IFIT1. GFP magnetic beads were then used to pull down IFIT1 and its bound RNA for Western blot analysis and RNA quantification (Fig 6M). The GFP beads specifically captured GFP-tagged IFIT1, which demonstrated the interaction of IFIT1 with Myc-LSDV012 (Fig 6K). Compared to the LSDV012 group, the total amount of ppp-RNA and A549 VACV RNA enriched in the IFIT1 group was significantly higher (Fig 6L). However, no significant difference was observed for Cap1 RNA. Previous studies have indicated that H5R is an early gene with transcripts containing Cap2, G8R is a mid-stage gene with transcripts also containing Cap2, and A17L is a late gene whose transcripts predominantly lacking Cap structures. Fluorescence PCR analysis of H5R, G8R, and A17L RNA before and after enrichment showed a similar pattern: more H5R ppp-RNA and A17L RNA were enriched in the presence of IFIT1 alone compared to IFIT1 co-expressed with LSDV012 (S3E-G Fig). Although there was a difference in RNA content, the variability observed in the fluorescence PCR results was less pronounced than anticipated. We speculated that the discrepancy might be attributed to the use of random primers for reverse transcription. Collectively, these data indicated that LSDV012 bound to the C-terminus of IFIT1 and sabotaged its binding capacity with ppp-RNA.

### 2.7 The loss of LSDV012 attenuates LSDV in a murine model

**Fig 7:**
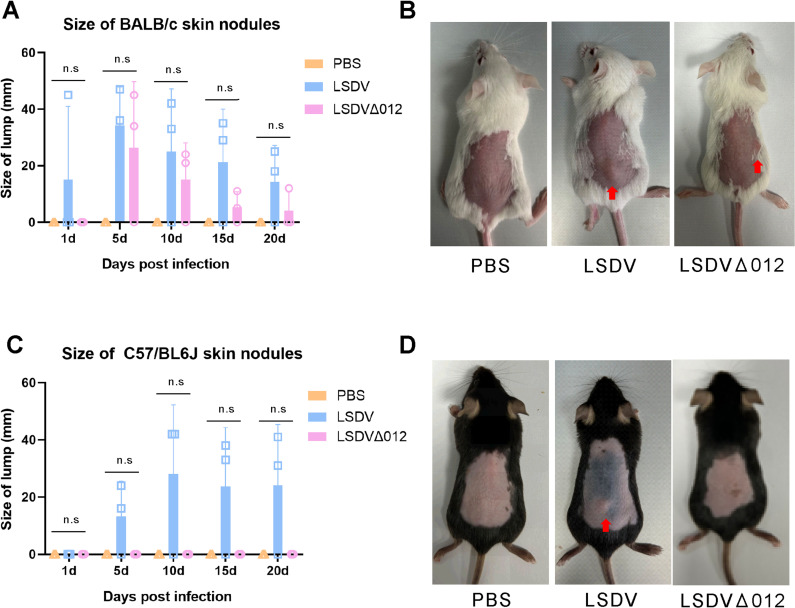
Evaluation of the pathogenicity of LSDV and LSDV Δ**012 in different mice models**. BALB/c (A and B) or C57/BL6J (C and D) mice were infected with LSDV or LSDVΔ012 via intradermal injection at an infectious dose of 10^7 pfu/ml. Photos (B and D) were taken by the authors.The nodule size at the injection site was measured at 1, 5, 10, 15, and 20 days post-infection.

As our data demonstrated that LSDV012 conferred resistance against type I IFN for LSDV by inhibiting cellular IFIT1, we further investigated if LSDV012 functions as a virulence factor using a murine model. In this model, intradermal injection of LSDV led to the formation of visible skin nodules (pox) in BALB/c and C57/BL6J mice. Five-week-old BALB/c or C57/BL6J mice were intradermally injected with 10^7 pfu of cushion-purified LSDV and LSDVΔ012. The formation and size of skin nodules were monitored and measured post-injection, with results plotted in Fig 7. Injection of both LSDV and LSDVΔ012 resulted in the formation of skin nodules from day 1 in BALB/c mice; however, the nodule sizes were significantly smaller in LSDVΔ012-injected mice. The difference was even more pronounced in C57/BL6J mice, where no skin nodules were observed in LSDVΔ012-injected mice. Despite this, only two-thirds of the mice exhibited nodule symptoms, and within-group differences rendered statistical analysis non-significant. This variability might be attributed to individual differences or the lower susceptibility of mice to the virus. In this model, LSDV infection did not lead to other pathological conditions in mice.

## 3 Discussion

LSDV encodes many unique viral proteins that are absent from VACV, and their biological functions remain largely unknown [23]. We demonstrated that LSDV012, an ankyrin protein encoded by a poxvirus, interacts with IFIT1/2/5. Liu et al. first reported VACV C9 as a type I IFN antagonist and demonstrated its working mechanism [12,37]. C9 contains an F-box motif at its C-terminus, which functions to recruit SCF components for proteasomal degradation. The amino acid sequence identity between VACV C9 and MPXV D9 is 90.9%, whereas it is only 14.9% between VACV C9 and LSDV012. Our phylogenetic analysis further corroborated these findings, showing that C9 and LSDV012 are positioned on distinct sister branches. Importantly, unlike VACV C9, LSDV012 does not contain an F-box domain in addition to the ankyrin repeats. Our analyses revealed that LSDV012 was unable to promote IFIT1 degradation as C9 could do. LSDV012 was able to bind to human and bovine IFIT1, IFIT2, and IFIT5, but not IFIT3. Interestingly, LSDV012 appeared to exhibit a stronger binding affinity than VACV C9, as ectopically expressed LSDV012 inhibited the degradation of IFIT1/2 promoted by VACV C9. These findings suggest that LSDV012 may share partial binding sites with VACV C9, allowing for competitive binding. To further characterize the association between IFIT1 and LSDV012, we selected and cloned more LSDV012 identified in our phylogenetic analysis to assess their binding capability with IFIT1 from various mammalian species, especially those that are natural hosts for the viruses that encode the corresponding LSDV012 orthologs. Orthologs of LSDV012 exist in many poxviruses such as goatpox virus (GTPV 010), deerpox virus (DPV014), tanapox virus (TPXV08), and eptesipox virus (EPTV169), and all these viruses demonstrate various host ranges. Rothenburg and colleagues made a series of discoveries showing that many poxviruses have evolved to optimally inhibit innate immune responses in their natural host [45–48]. This adaptation is typically achieved by the viral immune modulators evolving to interact effectively with host-specific antiviral factors [45]. Interestingly, we observed a very similar phenomenon in our analysis. Specifically, VACV C9 was able to interact with and led to the degradation of IFIT1 from human, bovine, and horseshoe bats. LSDV012 and DPV014, which are from poxviruses that typically infect ungulates, were able to bind to bovine IFIT1 and avert the degradation of the latter caused by VACV C9. In comparison, TPXV 08, an ortholog from the tanapox virus that naturally infects primates, exhibited the strongest interaction with human IFIT1, but with bovine and bat IFIT1 to a much lesser extent. Nevertheless, an exception was also found as the LSDV012 orthologs encoded by EPTV (EPTV169) failed to associate with bovine, human, or bat IFIT1. These demonstrated the complexity of host-poxvirus interaction and more than one factor would contribute to the host adaption during virus-host co-evolution.

Next, we aimed to explore the mechanism by which LSDV012 inhibits IFIT1 at a finer resolution. We first utilized Alphafold 3 to predict the interaction between IFIT1 and LSDV012 and found LSDV012 may interact with the C-terminus of IFIT1, which is essential for its RNA binding capability. Although molecular docking showed promising predictions, to validate experimentally, TPR domains annotated by the SMART database were serially truncated to assess their contribution to IFIT1-LSDV012 interaction. The biochemical analysis corroborated the association between the C-terminus of IFIT1 and LSDV012. To explore the importance of this association and to validate the hypothesis that LSDV012 shields the RNA binding pocket on IFIT1, an RNA pull-down assay was performed using mRNA transcribed in a cell-free system containing either triphosphate (ppp-RNA) at the 5’ end or cap1 (cap1-RNA). The RNA generated *in vitro* was only able to pull down IFIT1 when capped with triphosphate but not cap1. Importantly, the association between ppp-RNA and IFIT1 was inhibited by the presence of LSDV012, agreeing with our model that LSDV012 compromised IFIT1’s RNA binding ability.

IFIT1 exhibits two potential antiviral activities against VACV: (1) inhibition of cap 0 transcript translation (S2F and S2G Fig), and (2) suppression of genome replication (S2H and S2I Fig). Both Liu et al. [12]and our data indicate that poxviruses are sensitive to IFIT1, which selectively targets non-capped RNAs and mRNA lacking 2’ O-methylation. However, poxviruses possess capping enzymes and 2’-O-methytransferase. Therefore, how exactly IFIT1 attacks poxvirus mRNA is not fully understood. It has been reported that intermediate or late poxvirus transcripts contain poly-A leader sequences at the 5’ end of their transcripts [49]. The leader A structure facilitates viral protein synthesis [49]. Transcripts with leader A have been shown to lack a 5’ Cap [50], these might be detected and attacked by host IFIT1. Importantly, although IFITs affect poxvirus replication, they cannot completely prevent it. The titers of LSDVΔ012 caught up with WT-LSDV at a later time (72 hpi) of infection (Fig 1A-D), indicating that poxviruses may have other mechanisms to overcome type I IFN in the absence of LSDV012.

LSDV primarily infects cattle, and given the high cost of target animal experiments, exploring suitable small animal models remains an important and necessary endeavor. LSDV is also employed as a vaccine vector due to its ability to tolerate the insertion of large foreign DNA fragments and its capacity to efficiently express foreign antigens. It has been employed in vaccines against rabies virus [51], RVFV [52], and SARS-CoV-2 [53], demonstrating effective protection in experimental mouse models. Pivova, E. Y. et al. research showed no significant symptoms in mice infected intradermally at a dose of 1.8 × 10^5 TCID50 [54]. Our infection dose was over 2 logs higher, yet some mice still did not develop the disease. To a certain extent, our findings suggest that the deletion of LSDV012 attenuated LSDV in a murine model. Hamsters may be better suited to small animal models of LSDV [54, 55]. Nonetheless, further systematic evaluation in target animals is warranted.

## 4 Materials and methods

### Inclusion and Ethics Statement

The handling of live LSDV and animal experiments was conducted in a BSL-3 facility at the Lanzhou Veterinary Research Institute, Chinese Academy of Agricultural Sciences. All experimental procedures strictly followed animal welfare and ethical guidelines and were carried out in accordance with the regulations established by the Laboratory Animal Ethics Committee of the Lanzhou Veterinary Research Institute, Chinese Academy of Agricultural Sciences. Animal Ethics Review Number: LVRIAEC-2024-033.

### Cell culture

MDBK (#CL-0153), BHK-21 (#CL-0034), A549 (#MZ-0015), and 293T (#MZ-0005) cells were purchased from NingboMingZhoubioCO., Ltd. and cultured in DMEM (Solarbio, #11995) containing 10% FBS (Biological Industries, #04-001-1ACS), supplemented with 100 units/mL Penicillin-Streptomycin Liquid (Solarbio, #P1400). Cells were incubated in a cell incubator at 37˚C and supplemented with 5% CO2.

### Plasmids and transfection

Expression plasmids for pCMV-Myc-LSDV012, pCMV-FLAG-C9, pCMV-FLAG-D9, pCMV-eGFP-MX1, and pCMV-eGFP-IFIT1/2/3/5 were obtained from Tsingke Biotech. The IFIT1 truncated mutant expression plasmid was generated using PrimeSTAR HS (Takara, #R040A) for amplification and subsequently ligated into the pCMV-eGFP vector. All constructs were confirmed by DNA sequencing. Plasmid transfection was carried out using jetPRIME reagent (Polyplus, #101000046) according to the manufacturer’s instructions. Briefly, DNA was mixed with jetPRIME reagent and incubated for 10 min at room temperature. The transfection mix was then added to monolayers of cells drop-wise and incubated for 4 h at 37˚C in an incubator. Medium was replaced with fresh cell culture medium 6 h after transfection.

### siRNA transfection

siRNAs were designed by Andreas Pichlmair et al [25]. The sequences included siIFIT1_1 (CTCCTTGGGTCGTTCTACAAA), siIFIT1_2 (TACATGGGAGTTATCCATTGA), siIFIT5_1 (TACGTAAACCTTTGATACCTA), and siIFIT5_2 (CAGCCTTACGTCCTTCGTTAT), which were synthesized by Sangon Biotech (Shanghai) Co., Ltd. siRNA (50 pmol per well) was diluted in transfection buffer provided with the transfection reagent, then mixed with jetPRIME (Polyplus, #101000046) transfection reagent and incubated for 10 minutes at room temperature. The resulting siRNA-transfection reagent mixture was added to a monolayer of cells and incubated for 12 hours before viral infection.

### Construction of recombinant virus

The recombinant virus LSDVΔ012 was constructed by replacing the ORF012 with eGFP-ORF under the control of a synthesized poxvirus early/late promoter P11 (TTTCATTTTTTTTTTTTCTATGCTATAA) [56]. For the generation of the recombinant plasmid, primers 012-1 (caggtcgactctagaggatccAATTAAGCAAAAACATATCCTGTATTGTT), 012-2 (acaaaatgaaaAAAAGTTTTTTAAAAAAAATAAATGATGATAC), 012-3 (cgagctgtacaagtaatagTAGTAATTGATGATGGCAAACTAGCTT), and 012-4 (gagctcggtacccggggatccAGTGATGAAGGAGTATATTTATGTTCCAT) were used to amplify the upstream and downstream homology arms of LSDV012 locus. Additionally, primers 012-5 (aaaacttttTTTCATTTTGTTTTTTTCTATGCTATAAA) and 012-6 (aCTATTACTTGTACAGCTCGTGCATGC) were employed to amplify the GFP sequence containing the P11 promoter. The recombinant plasmid was then constructed using the ClonExpress Ultra One Step Cloning Kit V2 (Vazyme, cat#C116-01). The LSDV-eGFP virus was generated by inserting the P11-eGFP sequence between LSDV049 and LSDV050 [22]. Recombinant plasmid transfection and virus infection were performed using BHK cells. After 48 hours, the virus was harvested, and the recombinant virus was picked and plaque-purified using MDBK cells. The purification of viruses was performed using MDBK cells cultured in DMEM medium (Solarbio, cat#11995) containing 0.5% carboxymethylcellulose sodium (CMC, Solarbio, cat#C8621) and 2.5% FBS (Biological Industries, #04-001-1ACS). The resulting virus was clonally purified at least 4 times, and the successful generation of recombinant virus was confirmed by PCR and Sanger sequencing.

### Virus infection and plaque assay

Bovine IFNα (Cloud-Clone, #RPA033Bo01) and human IFNβ (GenScript, #Z03109) were used to pretreat MDBK or A549 cells, respectively. Various cell lines were infected with a multiplicity of infection (MOI) of 0.01, 0.1, or 3, and viral samples were harvested at different time points indicated in the figures. The collected samples were frozen and thawed three times and stored at -80˚C until use. Ultrasound treatment was applied at 80W for three cycles with 15 seconds on and 15 seconds off. Virus titers were determined by immunofluorescence using LSDV074 (H3L) antibody and fluorescent plaques were counted and recorded. Briefly, serially diluted viruses were prepared and used to infect MDBK cells for 2 hours. The medium was then replaced with DMEM containing 0.5% CMC and the cells were incubated for 72 h. The cells were then fixed (4% PFA for 15 min), permeabilized with 0.5% TritonX-100 for 15 min, and blocked with 3% bovine serum albumin (BSA) in PBS for 30 min. All statistical analyses were performed by two-sided Student’s t-test with GraphPad Prism 8. All data represent mean ± standard error of measurement (SEM).

### Construction of the MDBK 2×Myc-LSDV012 cell line

MDBK cells expressing the 2×Myc-tagged LSDV012 protein were generated through retroviral transduction. A eukaryotic codon-optimized LSDV012 ORF with an N-terminal 2×Myc tag (2×Myc-LSDV012) was cloned into the pLV-EF1a-IRES-Puro vector to generate pLV-EF1a-IRES-Puro-LSDV012. Retrovirus particles were produced by co-transfecting 293T cells with pLV-EF1a-IRES-Puro-LSDV012, pMD2.G, and psPAX2 using jetPRIME (Polyplus, #101000046). Cell medium containing retroviruses were harvested 48 hours after transfection and filtered (0.45µm). MDBK cells were infected with the retroviruses in the presence of 5 μg/ml Polybrene (Solarbio, #H8761) by spinoculation at 450 × g for 30 min at room temperature. The cells were then sub-cultured and passaged multiple times in a selection medium containing 2 μg/ml puromycin (Beyotime, #ST551) to establish stable expression. The synthesis of LSDV012 protein was confirmed by Western blotting using an HRP-conjugated anti-Myc antibody.

### RNA extraction, transcription, purification, and concentration determination

A549 cells were infected at a multiplicity of infection (MOI) of 0.1 with VACV-WR for 24 hours. RNA was extracted using the RNA extraction kit (Yeasen, cat#19231ES08). Trizol reagent (Yeasen, cat#19201ES60) was utilized for RNA pull-down product extraction. The RNA was subsequently reverse-transcribed using a cDNA Synthesis kit (Yeasen, cat#11156ES10). H5R ppp-mRNA and Cap1 mRNA were synthesized *in vitro*, and prepared for subsequent RNA pull-down assays. A T7 promoter (TAATACGACTCACTATAGGG) was incorporated at the 5’ end of the upstream primer during PCR amplification, and the linearized H5R DNA containing T7 promoter was amplified using VACV H5R as a template. For ppp-RNA transcription, the transcription system was prepared using the Novoprotein kit (Novoprotein, #E131) and included the following components: 10× Transcription Buffer (2 μl), ATP (100 mM, 1.5 μl), GTP (100 mM, 1.5 μl), CTP (100 mM, 1.5 μl), UTP (100 mM, 1.5 μl), Template DNA (5 μl), Enzyme Mix (1 μl), and RNase-Free Water to reach a final volume of 20 μl. For Cap1 RNA transcription, an additional 1.5 μl of Cap1 GAG (100 mM, Novoprotein, #S201) was included in the reaction mixture. For the synthesis of biotinylated ppp-RNA and Cap1 RNA, Biotin-16-UTP (50 mM, Roche, #11388908910) was used to replace UTP (50 mM) in the reaction. After mixing, the solution was briefly centrifuged to collect the contents at the bottom of the tube and incubated at 37ºC for 3 hours. Subsequently, 80 μl of Nuclease-free Water was added to the reaction mix, followed by the addition of 1 μl of DNase I. The mixture was then incubated at 37ºC for 15 minutes to remove the DNA template.

RNA purification was performed using the RNA clean kit (aidlab, Cat#RN62), and RNA electrophoresis was carried out using the Urea-PAGE Preparation Kit for RNA (Beyotime, R0218S). RNA concentration was measured using the NanoDrop 2000c Spectrophotometer. For consistency, we controlled the loading amounts of RNA at 20 µg.

### Fluorescence quantitative PCR

The levels of IFNα/β in MDBK, A549, and BHK-21 cells before and after LSDV infection were measured using real-time PCR with primers designed for IFNα or IFNβ. The ISG expression levels in cells stimulated with 2 ng/ml IFNα were also assessed, as well as the expression levels of H5R, G8R, and A17L before and after RNA pull-down. PCR amplification was performed using Yeasen (cat#11201ES03). Fluorescence quantitative PCR was performed in triplicates for each sample and statistical analysis using a one-way variance method. The primers used in this study are listed below:

**Table:** 

Primer name	Sequence 5’-3’	Target Gene
MDBK-IFNα-F	GTGAGGAAATACTTCCACAGACTCACT	XM_027535645.1
MDBK-IFNα-R	TGAGGAAGAGAAGGCTCTCATGA	
MDBK-IFNβ-F	CCTGTCCTGATTTCATCATGA	XM_027549142.1
MDBK-IFNβ-R	GCAAGCTGTAGCTCCTGGAAAG	
A549-IFNα-F	ATTCTGCACCGAACTCTACCAG	NM_024013.3
A549-IFNα-R	ATGGAGTCCGCATTCATCAGG	
A549-IFNβ-F	GATTCCTACAAAGAAGCAGCAA	NM_002176.4
A549-IFNβ-R	CAAAGTTCATCCTGTCCTTGAG	
BHK-IFNα-F	AGTGATTCATCTGCTCCTTGGG	XM_040757442.1
BHK-IFNα-R	CAGACAGGCTTGCAGGTTATTG	
BHK-IFNβ-F	AGAGACACTGCCTTCATCATCC	XM_040757177.1
BHK-IFNβ-R	TGGTCTTATTCCACCCAGTGC	
VACV-H5R-F	AGCCACTACTCCTCGTAAACC	
VACV-H5R-R	GGCCACGCTTTCTACAATGTC	
VACV-G8R-F	ACCTCTCAGTGCATCAGTGTTC	
VACV-G8R-R	CACGATTTTACCTTGGACACAGG	
VACV-A17L-F	GGTTTGATTTTGTTCGTCTTGGC	
VACV-A17L-R	GTTGCCATTACCTCCACGATAC	
ISG15-F	TGCAGAACTGCATCTCCATC	NM_174366.1
ISG15-R	TTCATGAGGCCGTATTCCTC	
GAPDH-F	TCACTGCCACCCAGAAGA	
GAPDH-R	CTCAGGGATGACCTTGCC	
LSDV011-F	CCAAACCACCATACTAAGTACAATTCCA	
LSDV011-R	ACCTAGCTGTAGTTCACCCAGT	
LSDV011-Probe	FAM-TCAATGCCGATAAGGA-MGB	
LSDV012-F	ACGCCATTGTTTTCGTGTGT	
LSDV012-R	ACTATTTCAGGTTCGACATTCTTGT	
LSDV013-F	GCTTTGGTTGTCTACAATCCGT	
LSDV013-R	CACAGGCTATGCCCAACAGA	
LSDV094-F	GGGATGAGTGCTCTTAACAGAC	
LSDV094-R	CAAGACATGCTCACAATAATGCG	

### Western blotting analysis and antibodies

Cells were lysed using a Cell Lysis Buffer for Western and IP (Beyotime, P0013), followed by centrifugation to remove cell debris. The lysates were then mixed with SDS-PAGE Sample Loading Buffer (Beyotime, P0015) and heated at 100°C for 5 minutes to denature the proteins. SDS-PAGE gels (12.5%) were prepared using the SDS-PAGE Gel Quick Preparation Kit (Beyotime, P0012AC). Protein transfer was conducted using the Turbo Transfer System (BIO-RAD). The following antibodies were used: IFIT1 Polyclonal Antibody (Proteintech, #23247-1-AP), IFIT2 Polyclonal Antibody (Proteintech, #12604-1-AP), IFIT3 Polyclonal Antibody (Proteintech, # 15201-1-AP), IFIT5 Polyclonal Antibody (Proteintech, # 13378-1-AP), GFP Tag Monoclonal Antibody (Proteintech, #66002-1-Ig), Myc Tag Mouse Monoclonal Antibody (Beyotime, #AF0033), Flag Tag Mouse Monoclonal Antibody (Beyotime, #AF2852), GAPDH Mouse Monoclonal Antibody (Beyotime, #AF0006), and Actin Mouse Monoclonal Antibody (Beyotime, #AA128). LSDV074 (H3L) Mouse Monoclonal Antibody, an intermediate/late protein commonly used for capripoxvirus detection [36,57], was generously provided by Dr. Chunsheng Yin (China Institute of Veterinary Drug Control, IVDC). Rabbit anti-LSDV serum was prepared in our laboratory using LSDV virus inactivated at 65°C and mixed with an immune adjuvant (Biodragon, cat#KX0210045). Western blotting analyses were repeated three times and band densities were measured and analyzed using ImageJ software, with GAPDH or Actin serving as the normalization reference.

### Confocal microscopy

BHK-21 cells seeded on coverslips were transfected with pCMV-Myc-LSDV012 and pCMV-eGFP-IFIT1, either individually or together. Twenty-four hours post-transfection, cells were fixed with 4% paraformaldehyde for 20 minutes and permeabilized with 0.1% Triton X-100 for 20 minutes at room temperature. Cells were blocked with 3% BSA-PBS for 1 hour at room temperature. Primary antibodies, diluted in 3% BSA-PBS, were incubated with cells overnight at 4°C. The samples were then washed with PBS four times and incubated with secondary antibodies conjugated with Alexa Fluor 488 or Alexa Fluor 594. Nuclei were stained with DAPI, and cells were washed multiple times before mounting on slides using ProLong Diamond Antifade reagent (ThermoFisher, cat#P36971). Images were captured using a Leica SP8 confocal microscope and processed with the Leica software (Leica Biosystems).

### Alignment of Ankyrin genes and protein structure prediction

The number of ankyrin genes across 63 strains from different poxvirus genera was determined using the HMM model ANK_2 (Pfam: PF12796) from the Pfam database. Strain-specific information is detailed in Table 1. Ankyrin gene counts were visualized using ggplot2 [58]. For protein domain analysis, the interaction of IFIT1 (XP_027384736.1) and LSDV012 (AAK84973.1) was examined using the SMART database [43]. Protein structures were predicted using AlphaFold3 [42]. InterfaceResidues was utilized to identify potential interaction sites between these two proteins by PyMOL.

### Immunoprecipitation and mass spectrometry

For most co-immunoprecipitation experiments in this study, Myc magnetic beads were used. 293T or BHK-21 cells were transfected with plasmids for 24 h. Cell proteins were collected using a Cell Lysis Buffer for Western and IP (Beyotime, cat#P0013), and mouse IgG magnetic beads were pretreated for 2 h for pre-clearing. Co-incubation was performed with myc beads for 4 h and beads were washed 5 times using TBS. Add protein loading buffer boil for 5 min, and let stand for 30 s using a magnetic stand to remove the remaining magnetic beads. Protein content was detected using Western blotting analysis.

For the identification of the interacting partners of LSDV012, MDBK cells, and MDBK-LSDV012 cells were stimulated with IFNα (20ng/ml) for 24 h. Cells were lysed using Cell Lysis Buffer (Beyotime, cat#P0013), centrifuged at 12,000 rpm at 4°C for 10 minutes, and the supernatant was collected. An appropriate amount of Anti-Myc magnetic beads was taken and washed five times with TBS. The magnetic beads were then mixed with the cell lysate supernatant, placed on a rotary mixer, and incubated overnight at 4°C. After incubation, the beads were placed on a magnetic stand for 10 seconds to remove the supernatant. The beads were washed three times with TBS and then 100 μl of 1× SDS-PAGE loading buffer was added. The mixture was heated at 95°C for 5 minutes, placed on a magnetic stand for 10 seconds, and the supernatant was collected for SDS-PAGE electrophoresis. Protein identification was conducted using a Q-Exactive high-resolution mass spectrometer (Thermo Fisher Scientific, Waltham, MA, USA) coupled to a Nano-Acquity High-Performance Liquid Chromatography (HPLC) system (Waters, Milford, MA, USA) at the mass spectrometry laboratory of the Chinese Agricultural University. Peptides were initially loaded onto a trap column (Acclaim PepMap, 75 µm × 2 cm, 3 µm, C18, 100 Å; Thermo Fisher Scientific) and subsequently separated on an analytical column (Aqua, 100 µm × 15 cm, 3 µm, C18, 125 Å; Phenomenex, Los Angeles, CA, USA) at a flow rate of 400 nL/min. The peptide elution was achieved using a 125-minute gradient with mobile phase A consisting of 0.1% formic acid (FA) in water and mobile phase B comprising 0.1% FA in acetonitrile. Mass spectrometry (MS) survey scans were acquired in the m/z range of 300 to 1800 with a resolution of 70,000. The instrument targeted the 10 most intense peptide signals from each full-scan spectrum for higher-energy collisional dissociation (HCD), maintaining a dynamic exclusion time of 20 seconds to reduce redundancy.

Mass spectrometry (MS) data were processed using MASCOT (version 2.4; Matrix Science, London, UK) and validated with Scaffold software (version 3.6.5; Proteome Software, Portland, USA). To ensure high data quality, proteins identified as common contaminants were excluded. The remaining protein sequences were matched against the UniProt Bovine database and a custom poxvirus database to identify host and viral proteins, respectively. Additionally, a subset of proteins observed in the negative control group was removed during data analysis to minimize false-positive identifications. The mass spectrometry proteomics data have been deposited in the ProteomeXchange Consortium (https://proteomecentral.proteomexchange.org) via the iProX partner repository [59, 60], with the dataset identifier PXD056306. During data analysis, the subset observed in the negative control group was excluded.

To examine the impact of LSDV012 on the RNA binding capacity of IFIT, an RNA Pull-Down kit (Fitgene, #FI8702) was employed. An equal amount of Biotin-ppp-RNA or biotin-Cap1-RNA (20µg, measured with NanoDrop), was denatured at 95°C for 3 minutes, followed by cooling on ice for 1 minute. The RNA was then mixed with 50 μl of RNA structure buffer and incubated at room temperature for 30 minutes. The RNA mixture was then combined with streptavidin magnetic beads, placed on a mixer, and incubated at room temperature for 2 h. After incubation, the beads were placed on a magnetic stand for 1 minute to remove the supernatant, and the beads were washed twice with PBS. The sample protein lysate (containing appropriate amounts of protease inhibitors and RNase inhibitors) was added and incubated at 4°C on a mixer for 4 hours. The beads were washed three times with rinse solution, placed on a magnetic stand for 1 minute, and the supernatant was discarded. Finally, SDS-PAGE loading buffer was added to the beads, heated at 95°C for 3 minutes, and placed on a magnetic stand for 1 minute, and the supernatant was collected for Western blotting detection.

For the pull-down of GFP/GFP-IFIT1, we employed the RNA Immunoprecipitation Kit (Bersinbio, #Bes5101). Approximately 20 µg of A549 VACV RNA, ppp-RNA, or Cap1-RNA was incubated with cell lysates, which included protease and RNase inhibitors, at 4°C for 12 hours. GFP magnetic beads were subsequently introduced and incubated for an additional 2 hours. Next, the beads were placed on a magnetic stand for 1 minute to separate the supernatant, followed by five thorough washes with rinse solution (provided in the kit). SDS-PAGE loading buffer was then added to the sample, heated to 95°C for 3 minutes, and placed back on the magnetic stand to collect the supernatant for Western blot analysis. For RNA examination, a fraction of the sample was treated with Trizol, and an equal volume of RNase-free water was added. The RNA concentration was measured using a spectrophotometer, followed by reverse transcription and quantification of nucleic acid content through fluorescence qPCR.

### Mouse infection experiments

Purified LSDV or LSDVΔ012 virus was diluted in PBS and (100 μl with 107 pfu) an equal amount of viruses were used to inject 5-week-old BALB/c and C57/BL6J mice intradermally. The nodule size at the injection site was measured at 1, 5, 10, 15, and 20 days post-infection.

## Supporting information

S1 Fig Construction of LSDVΔ012 virus and its effect on IFNα/β mRNA expression in different cell lines . Construction and purification processes for LSDVΔ012 were conducted and validated (A). PCR was performed to detect modifications in LSDV and LSDVΔ012 (B). MDBK cells were treated with varying concentrations of IFNα. After 24 hours, they were infected with LSDV at an MOI of 0.01, and viral titers were measured 48 hours post-infection (C). A549, MDBK, and BHK-21 cells were infected with LSDV at an MOI of 0.1. IFNα/β mRNA expression levels were measured 24 hours post-infection (D) ΔCT values represent the difference in threshold cycle (ct) values between the target gene and a reference gene (GAPDH). The lower the Δct value, the higher the expression of the target gene relative to the reference gene. A diagram illustrating the construction of the MDBK-012 cell line stably expressing Myc-LSDV012. Additionally, the screening process for MDBK cells stably expressing LSDV012 is shown (E). MDBK cells were treated with 2 ng/ml IFNα for 24 hours, and ISG15 mRNA expression levels were assessed (F). White light photographs were captured from MDBK cells pre-treated with 2 ng/ml IFNα for 24 hours and infected with LSDV or LSDVΔ012 at 0.01 MOI for 48h (G). MDBK cells were pre-treated with 2 ng/ml IFNα for 24 hours, followed by infection with LSDV or LSDVΔ012 at an MOI of 3. Viral titers were measured after 48 hours (H), and the expression level of viral H3 protein was evaluated (I and J). Significance Levels: *p < 0.05, ** p < 0.01, *** p < 0.001, n.s: non-significant.
(DOCX)

S2 Fig LSDV012 Redirects IFIT1 to the Perinuclear Region. Myc-LSDV012 and GFP-IFIT1 were co-transfected or separately transfected into BHK-21 cells for 24 hours. Cells were fixed on coverslips and fluorescent images were captured using confocal microscopy (A). BHK-21 cells were transfected with IFIT1/2/3/5 for 24h, followed by infection with VACV WR (B) or LSDV (C) for 24 hours. The impact of the virus on the expression levels of IFIT1, IFIT2, IFIT3, and IFIT5 was then assessed. BHK-21 cells were transfected with the same dose of IFIT1 and VACV C9, and different doses of LSDV012 (0.01 ng, 0.1 ng, 0.5 ng, 1 ng) for 24 h, and the samples were harvested to detect the content of different proteins (D).A549 cells were transfected with siRNA targeting IFIT1 or IFIT5 for 12 hours, followed by treatment with IFNβ for 12 hours, then continued to incubate for 48h. The expression levels of IFIT1 and IFIT5 proteins were then measured (E and F). BHK-21 cells were infected with LSDV at 0.1 moi for 24 h, and rabbit anti-LSDV serum was incubated with infected and uninfected BHK-21 cell proteins to evaluate the effect of antiserum (G). BHK-21 cells were transfected with GFP-IFIT1 for 24 h, LSDV/LSDVΔ012 was infected with 0.01 moi for 48 h, and the viral protein content was detected with rabbit anti-LSDV serum (H). A standard curve between the copy number of the plasmid and the Ct value was constructed using real-time PCR (probe) for LSDV011 (I). BHK-21 cells were transfected with GFP-IFIT1 for 24h, LSDV/LSDVΔ012 was infected with 0.1 moi for 24h, and the viral DNA copy number was determined by real-time PCR (probe) for LSDV011 (J). Significance Levels: *p < 0.05, ** p < 0.01, *** p < 0.001, n.s: non-significant.
(DOCX)

S3 Fig Analysis of Poxvirus-Ankyrin Proteins and RNA Detection . The number of ankyrin proteins encoded by various poxvirus species is illustrated. The size of the circles represents the quantity, while the color indicates the specific species (A). The structural prediction of LSDV012 was performed using Alphafold3, displaying its predicted protein structure (B). The in vitro transcribed VACV H5R ppp-RNA, Cap1-RNA, biotin-ppp-RNA, and biotin-Cap1-RNA were detected using RNA PAGE (C). RNA concentration was measured using a spectrophotometer at a concentration of approximately 1234.67±28.6 ng/μl for ppp-RNA, 1186.53±4.12 ng/μl for Cap1-RNA, 1237.833±20.04 ng/μl for biotin-ppp-RNA, and 1267.7±39.92 ng/μl for biotin-Cap1-RNA. For consistency, we controlled the loading amounts of all RNA at 20 µg (D). The RNA levels of H5 (E), A17 (F), and G8 (G) were detected using fluorescent quantitative PCR before and after the pull-down with GFP magnetic beads. ΔCT values represent the difference in threshold cycle (ct) values between the target gene and a reference gene (GAPDH). The lower the ΔCT value, the higher the expression of the target gene relative to the reference gene. Significance Levels: *p < 0.05, ** p < 0.01, *** p < 0.001, n.s: non-significant.
(DOCX)

S1 Table The group of 636 ankyrin proteins encoded by the poxvirus.
(DOCX)

S2 Table VACV C9L homologs in different poxvirus.
(DOCX)

S3 Table LSDV012 interacts with IFIT1 molecule to predict amino acid sites.
(DOCX)

S4 Table The mass spectrometry proteomics data by Myc beads CoIP.
(XLSX)

S5 Table The dataset used to build images in this article.
(XLSX)

S1 Data The raw data of western blot and IF in this study.
(PDF)

S2 Data The sequence of IFN DNA in different cells.
(ZIP)
