# Supplementary material for: A poxvirus ankyrin protein LSDV012 inhibits IFIT1 in a host-species-specific manner by compromising its RNA binding ability
Source: PLoS Pathog. 2025 Mar 17;21(3):e1012994. doi: 10.1371/journal.ppat.1012994 (PMC11957390; doi:10.1371/journal.ppat.1012994)
Supplement: S1 Fig — . Construction and purification processes for LSDVΔ012 were conducted and validated (A). PCR was performed to detect modifications in LSDV and LSDVΔ012 (B). MDBK cells were treated with varying concentrations of IFNα. After 24 hours, they were infected with LSDV at an MOI of 0.01, and viral titers were measured 48 hours post-infection (C). A549, MDBK, and BHK-21 cells were infected with LSDV at an MOI of 0.1. IFNα/β mRNA expression levels were measured 24 hours post-infection (D) ΔCT values represent the difference in threshold cycle (ct) values between the target gene and a reference gene (GAPDH). The lower the Δct value, the higher the expression of the target gene relative to the reference gene. A diagram illustrating the construction of the MDBK-012 cell line stably expressing Myc-LSDV012. Additionally, the screening process for MDBK cells stably expressing LSDV012 is shown (E). MDBK cells were treated with 2 ng/ml IFNα for 24 hours, and ISG15 mRNA expression levels were assessed (F). White light photographs were captured from MDBK cells pre-treated with 2 ng/ml IFNα for 24 hours and infected with LSDV or LSDVΔ012 at 0.01 MOI for 48h (G). MDBK cells were pre-treated with 2 ng/ml IFNα for 24 hours, followed by infection with LSDV or LSDVΔ012 at an MOI of 3. Viral titers were measured after 48 hours (H), and the expression level of viral H3 protein was evaluated (I and J). Significance Levels: *p < 0.05, ** p < 0.01, *** p < 0.001, n.s: non-significant. (DOCX) [file ppat.1012994.s001.docx]

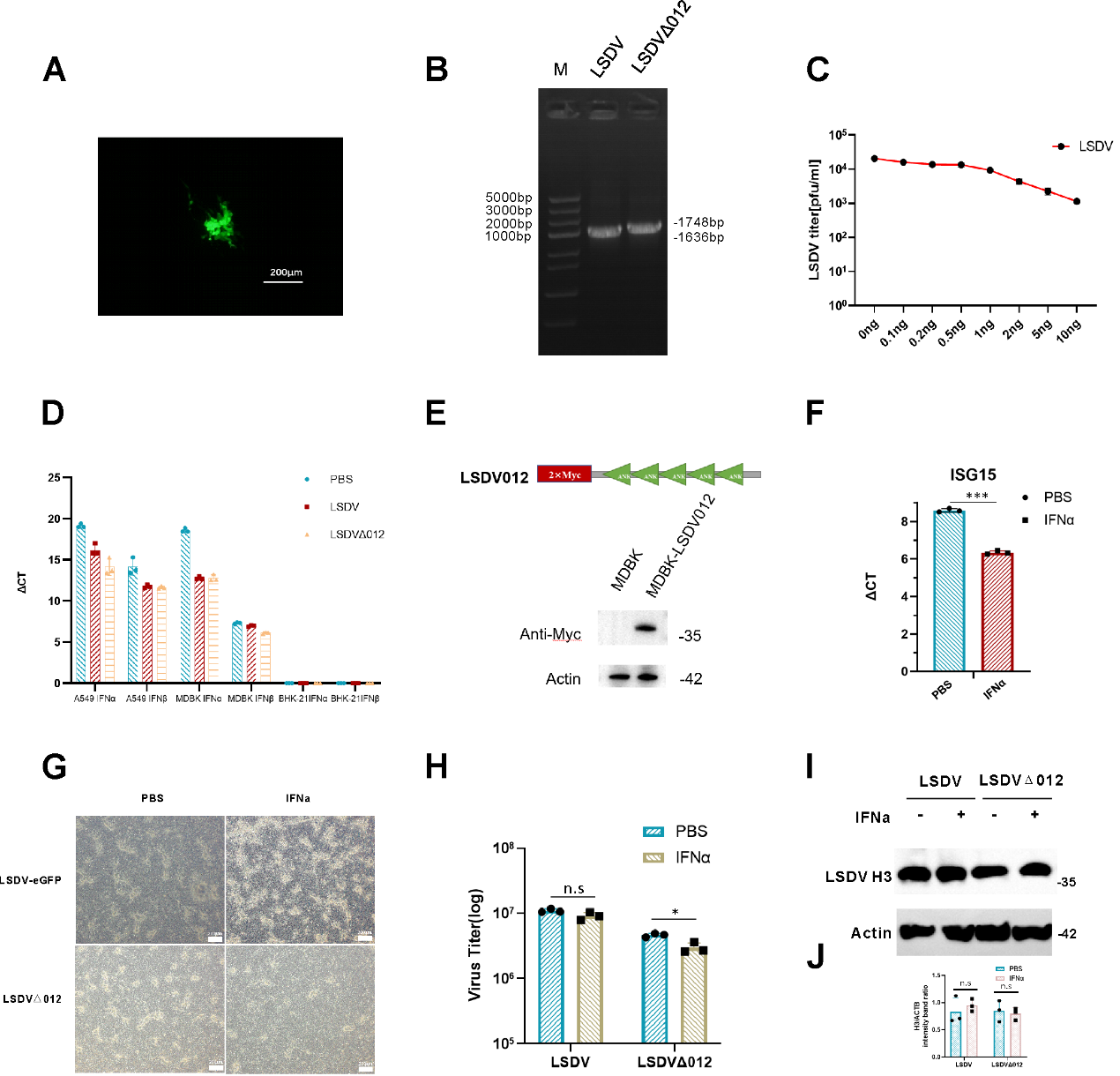


**S1 Fig. Construction of LSDVΔ012 virus and its effect on IFNα/β mRNA expression in different cell lines.**

Construction and purification processes for LSDVΔ012 were conducted and validated (A). PCR was performed to detect modifications in LSDV and LSDVΔ012 (B). MDBK cells were treated with varying concentrations of IFNα. After 24 hours, they were infected with LSDV at an MOI of 0.01, and viral titers were measured 48 hours post-infection (C). A549, MDBK, and BHK-21 cells were infected with LSDV at an MOI of 0.1. IFNα/β mRNA expression levels were measured 24 hours post-infection (D) ΔCT values represent the difference in threshold cycle (ct) values between the target gene and a reference gene (GAPDH). The lower the Δct value, the higher the expression of the target gene relative to the reference gene. A diagram illustrating the construction of the MDBK-012 cell line stably expressing Myc-LSDV012. Additionally, the screening process for MDBK cells stably expressing LSDV012 is shown (E). MDBK cells were treated with 2 ng/ml IFNα for 24 hours, and ISG15 mRNA expression levels were assessed (F). White light photographs were captured from MDBK cells pre-treated with 2 ng/ml IFNα for 24 hours and infected with LSDV or LSDVΔ012 at 0.01 MOI for 48h (G). MDBK cells were pre-treated with 2 ng/ml IFNα for 24 hours, followed by infection with LSDV or LSDVΔ012 at an MOI of 3. Viral titers were measured after 48 hours (H), and the expression level of viral H3 protein was evaluated (I and J). Significance Levels: *p < 0.05, ** p < 0.01, *** p < 0.001, n.s: non-significant.
