# Supplementary material for: A poxvirus ankyrin protein LSDV012 inhibits IFIT1 in a host-species-specific manner by compromising its RNA binding ability
Source: PLoS Pathog. 2025 Mar 17;21(3):e1012994. doi: 10.1371/journal.ppat.1012994 (PMC11957390; doi:10.1371/journal.ppat.1012994)
Supplement: S2 Fig — Myc-LSDV012 and GFP-IFIT1 were co-transfected or separately transfected into BHK-21 cells for 24 hours. Cells were fixed on coverslips and fluorescent images were captured using confocal microscopy (A). BHK-21 cells were transfected with IFIT1/2/3/5 for 24h, followed by infection with VACV WR (B) or LSDV (C) for 24 hours. The impact of the virus on the expression levels of IFIT1, IFIT2, IFIT3, and IFIT5 was then assessed. BHK-21 cells were transfected with the same dose of IFIT1 and VACV C9, and different doses of LSDV012 (0.01 ng, 0.1 ng, 0.5 ng, 1 ng) for 24 h, and the samples were harvested to detect the content of different proteins (D).A549 cells were transfected with siRNA targeting IFIT1 or IFIT5 for 12 hours, followed by treatment with IFNβ for 12 hours, then continued to incubate for 48h. The expression levels of IFIT1 and IFIT5 proteins were then measured (E and F). BHK-21 cells were infected with LSDV at 0.1 moi for 24 h, and rabbit anti-LSDV serum was incubated with infected and uninfected BHK-21 cell proteins to evaluate the effect of antiserum (G). BHK-21 cells were transfected with GFP-IFIT1 for 24 h, LSDV/LSDVΔ012 was infected with 0.01 moi for 48 h, and the viral protein content was detected with rabbit anti-LSDV serum (H). A standard curve between the copy number of the plasmid and the Ct value was constructed using real-time PCR (probe) for LSDV011 (I). BHK-21 cells were transfected with GFP-IFIT1 for 24h, LSDV/LSDVΔ012 was infected with 0.1 moi for 24h, and the viral DNA copy number was determined by real-time PCR (probe) for LSDV011 (J). Significance Levels: *p < 0.05, ** p < 0.01, *** p < 0.001, n.s: non-significant. (DOCX) [file ppat.1012994.s002.docx]

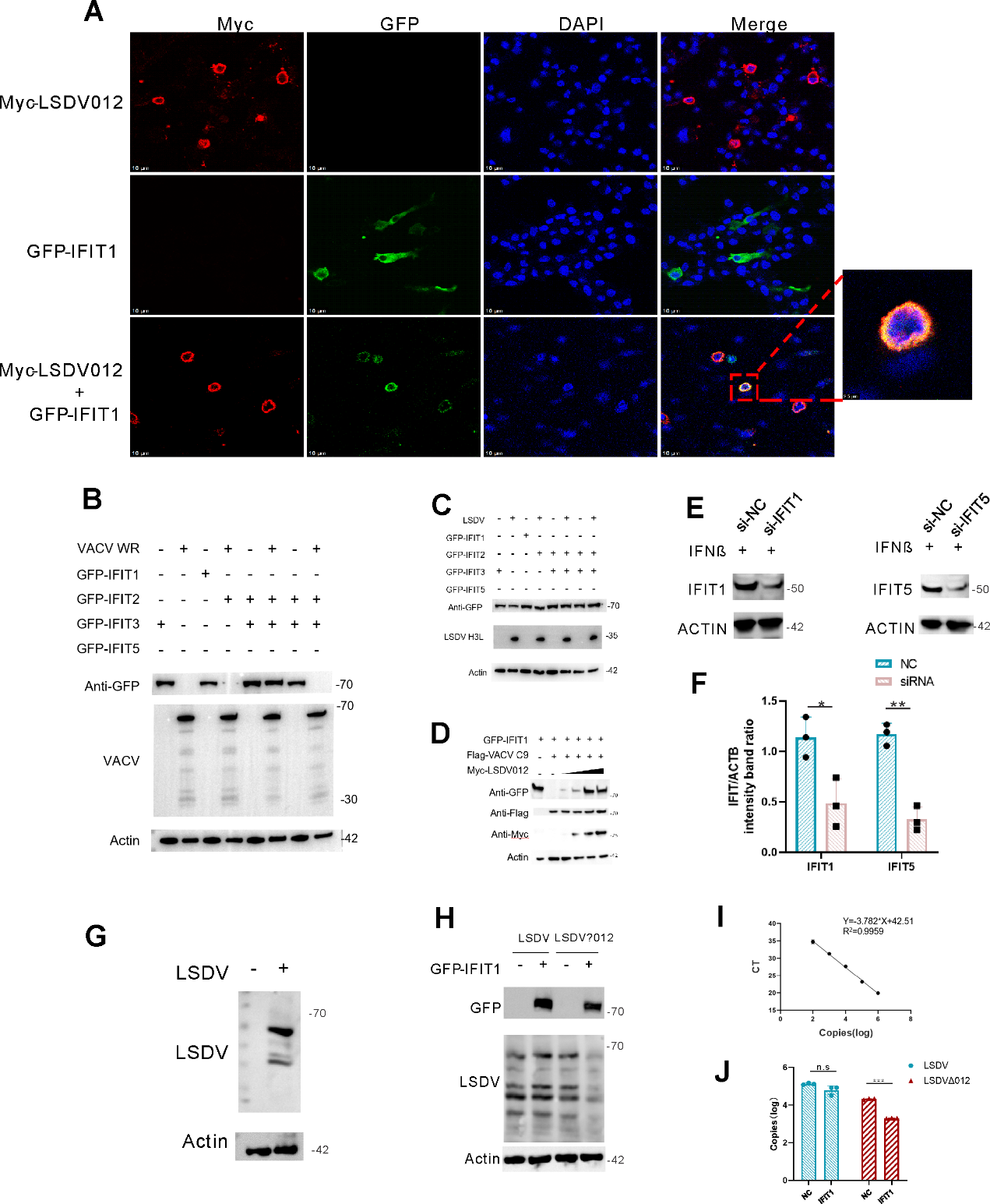


**S2 Fig. LSDV012 Redirects IFIT1 to the Perinuclear Region.**

Myc-LSDV012 and GFP-IFIT1 were co-transfected or separately transfected into BHK-21 cells for 24 hours. Cells were fixed on coverslips and fluorescent images were captured using confocal microscopy (A). BHK-21 cells were transfected with IFIT1/2/3/5 for 24h, followed by infection with VACV WR (B) or LSDV (C) for 24 hours. The impact of the virus on the expression levels of IFIT1, IFIT2, IFIT3, and IFIT5 was then assessed. BHK-21 cells were transfected with the same dose of IFIT1 and VACV C9, and different doses of LSDV012 (0.01 ng, 0.1 ng, 0.5 ng, 1 ng) for 24 h, and the samples were harvested to detect the content of different proteins (D).

A549 cells were transfected with siRNA targeting IFIT1 or IFIT5 for 12 hours, followed by treatment with IFNβ for 12 hours, then continued to incubate for 48h. The expression levels of IFIT1 and IFIT5 proteins were then measured (E and F). BHK-21 cells were infected with LSDV at 0.1 moi for 24 h, and rabbit anti-LSDV serum was incubated with infected and uninfected BHK-21 cell proteins to evaluate the effect of antiserum (G). BHK-21 cells were transfected with GFP-IFIT1 for 24 h, LSDV/LSDVΔ012 was infected with 0.01 moi for 48 h, and the viral protein content was detected with rabbit anti-LSDV serum (H). A standard curve between the copy number of the plasmid and the Ct value was constructed using real-time PCR (probe) for LSDV011 (I). BHK-21 cells were transfected with GFP-IFIT1 for 24h, LSDV/LSDVΔ012 was infected with 0.1 moi for 24h, and the viral DNA copy number was determined by real-time PCR (probe) for LSDV011 (J). Significance Levels: *p < 0.05, ** p < 0.01, *** p < 0.001, n.s: non-significant.
