# Supplementary material for: A poxvirus ankyrin protein LSDV012 inhibits IFIT1 in a host-species-specific manner by compromising its RNA binding ability
Source: PLoS Pathog. 2025 Mar 17;21(3):e1012994. doi: 10.1371/journal.ppat.1012994 (PMC11957390; doi:10.1371/journal.ppat.1012994)
Supplement: S3 Fig — . The number of ankyrin proteins encoded by various poxvirus species is illustrated. The size of the circles represents the quantity, while the color indicates the specific species (A). The structural prediction of LSDV012 was performed using Alphafold3, displaying its predicted protein structure (B). The in vitro transcribed VACV H5R ppp-RNA, Cap1-RNA, biotin-ppp-RNA, and biotin-Cap1-RNA were detected using RNA PAGE (C). RNA concentration was measured using a spectrophotometer at a concentration of approximately 1234.67±28.6 ng/μl for ppp-RNA, 1186.53±4.12 ng/μl for Cap1-RNA, 1237.833±20.04 ng/μl for biotin-ppp-RNA, and 1267.7±39.92 ng/μl for biotin-Cap1-RNA. For consistency, we controlled the loading amounts of all RNA at 20 µg (D). The RNA levels of H5 (E), A17 (F), and G8 (G) were detected using fluorescent quantitative PCR before and after the pull-down with GFP magnetic beads. ΔCT values represent the difference in threshold cycle (ct) values between the target gene and a reference gene (GAPDH). The lower the ΔCT value, the higher the expression of the target gene relative to the reference gene. Significance Levels: *p < 0.05, ** p < 0.01, *** p < 0.001, n.s: non-significant. (DOCX) [file ppat.1012994.s003.docx]

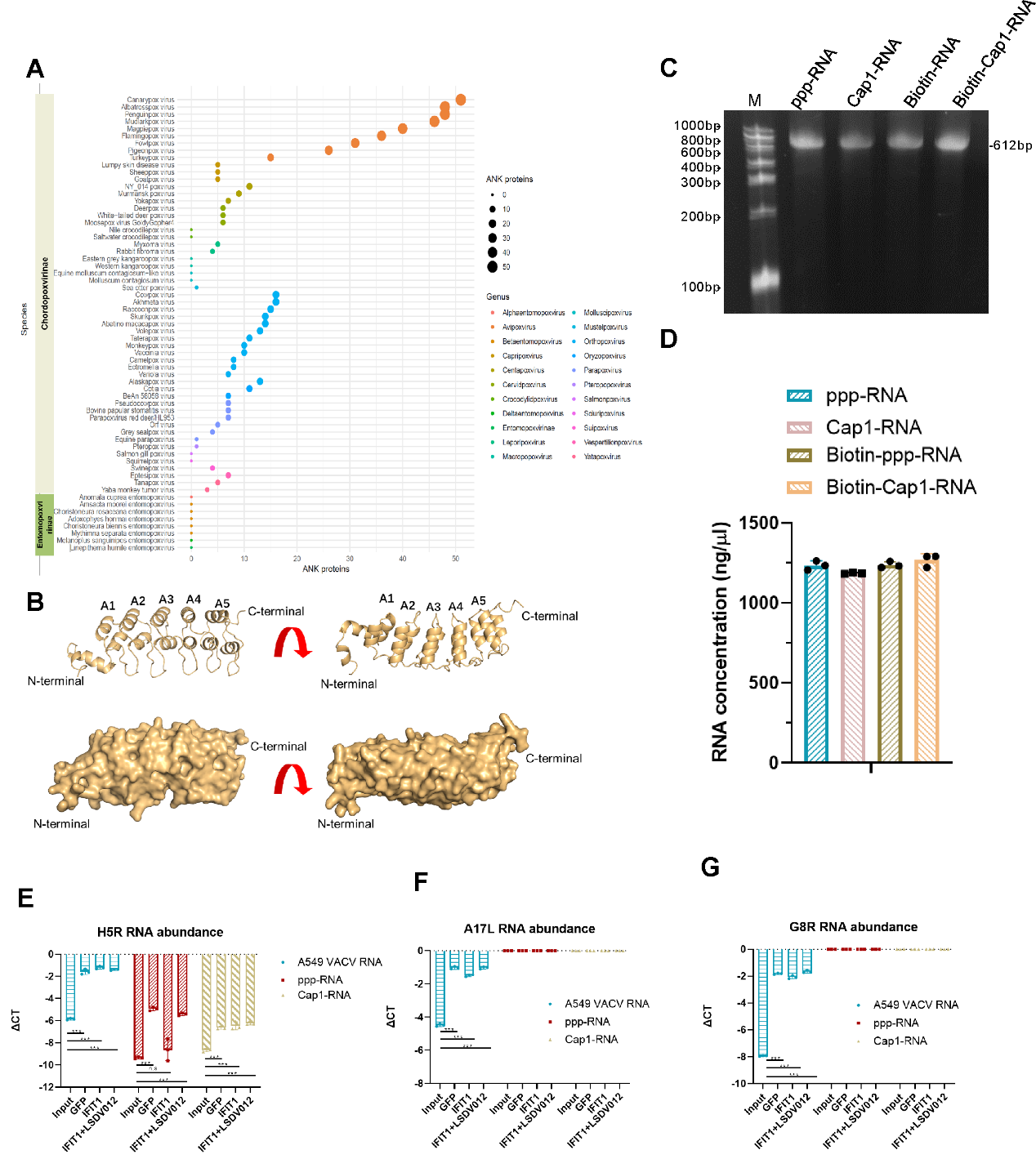


**S3 Fig. Analysis of Poxvirus-Ankyrin Proteins and RNA Detection.**

The number of ankyrin proteins encoded by various poxvirus species is illustrated. The size of the circles represents the quantity, while the color indicates the specific species (A). The structural prediction of LSDV012 was performed using Alphafold3, displaying its predicted protein structure (B). The in vitro transcribed VACV H5R ppp-RNA, Cap1-RNA, biotin-ppp-RNA, and biotin-Cap1-RNA were detected using RNA PAGE (C). RNA concentration was measured using a spectrophotometer at a concentration of approximately 1234.67±28.6 ng/μl for ppp-RNA, 1186.53±4.12 ng/μl for Cap1-RNA, 1237.833±20.04 ng/μl for biotin-ppp-RNA, and 1267.7±39.92 ng/μl for biotin-Cap1-RNA. For consistency, we controlled the loading amounts of all RNA at 20 µg (D). The RNA levels of H5 (E), A17 (F), and G8 (G) were detected using fluorescent quantitative PCR before and after the pull-down with GFP magnetic beads. ΔCT values represent the difference in threshold cycle (ct) values between the target gene and a reference gene (GAPDH). The lower the ΔCT value, the higher the expression of the target gene relative to the reference gene. Significance Levels: *p < 0.05, ** p < 0.01, *** p < 0.001, n.s: non-significant.
