# Supplementary material for: A poxvirus ankyrin protein LSDV012 inhibits IFIT1 in a host-species-specific manner by compromising its RNA binding ability
Source: PLoS Pathog. 2025 Mar 17;21(3):e1012994. doi: 10.1371/journal.ppat.1012994 (PMC11957390; doi:10.1371/journal.ppat.1012994)
Supplement: S1 Table — (DOCX) [file ppat.1012994.s004.docx]

S1 Table. The group of 636 ankyrin proteins encoded by the poxvirus.

| Virus | Gene | ID | Group | Virus | Gene | ID | Group |
| --- | --- | --- | --- | --- | --- | --- | --- |
| Ectromelia virus | EVM021 | AAM92327 | ANK1 | Mudlarkpox virus | ankyrin | QRM15595 | ANK17 |
| Monkeypox virus | O1L | AAL40483 | ANK1 | Penguinpox virus | ankyrin | QRM15640 | ANK17 |
| Camelpox virus | ankyrin | AAL73735 | ANK1 | Penguinpox virus | ankyrin | QRM15650 | ANK17 |
| Cowpox virus | CPXV039 | AAM13486 | ANK1 | Penguinpox virus | ankyrin | QRM15777 | ANK17 |
| Vaccinia virus | VACWR030 | AAO89309 | ANK1 | Penguinpox virus | ankyrin | QRM15923 | ANK17 |
| Taterapox virus | TATV_DAH68_032 | ABD97598 | ANK1 | Penguinpox virus | ankyrin | QRM15942 | ANK17 |
| Yokapox virus | YKV015c | AEN03604 | ANK1 | Penguinpox virus | ankyrin | QRM15948 | ANK17 |
| Raccoonpox virus | RCNV-Herman-026 | AKJ93660 | ANK1 | Penguinpox virus | ankyrin | QRM15951 | ANK17 |
| Skunkpox virus | SKPV-WA-029 | AOP31508 | ANK1 | Albatrosspox virus | ankyrin | QRM15970 | ANK17 |
| Volepox virus | VPXV-CA-029 | AOP31719 | ANK1 | Albatrosspox virus | ankyrin | QRM15980 | ANK17 |
| Murmansk poxvirus | Murmansk-028 | AST09223 | ANK1 | Albatrosspox virus | ankyrin | QRM16109 | ANK17 |
| NY_014 poxvirus | NY_014-024 | AST09425 | ANK1 | Albatrosspox virus | ankyrin | QRM16260 | ANK17 |
| Akhmeta virus | AKMV-88-035 | AXN74820 | ANK1 | Albatrosspox virus | ankyrin | QRM16279 | ANK17 |
| Alaskapox virus | ankyrin-like | QED21136 | ANK1 | Albatrosspox virus | ankyrin | QRM16285 | ANK17 |
| Abatino macacapox virus | KM543_gp032 | YP_010085700 | ANK1 | Albatrosspox virus | ankyrin | QRM16288 | ANK17 |
| Variola virus | O1L | CAA48959 | ANK1 | Magpiepox virus | MPPV-011 | QZW33278 | ANK17 |
| Ectromelia virus | EVM154 | AAM92459 | ANK2 | Magpiepox virus | MPPV-152 | QZW33447 | ANK17 |
| Monkeypox virus | B5R | AAL40624 | ANK2 | Magpiepox virus | MPPV-335 | QZW33664 | ANK17 |
| Camelpox virus | ankyrin | AAL73887 | ANK2 | Magpiepox virus | MPPV-341 | QZW33670 | ANK17 |
| Cowpox virus | CPXV198 | AAM13637 | ANK2 | Fowlpox virus | FPV244 | AAF44588 | ANK18 |
| Vaccinia virus | VACWR186 | AAO89465 | ANK2 | Canarypox virus | CNPV009 | AAR83355 | ANK18 |
| Taterapox virus | TATV_DAH68_187 | ABD97753 | ANK2 | Pigeonpox virus | fep_010 | AID46526 | ANK18 |
| Raccoonpox virus | RCNV-Herman-177 | AKJ93810 | ANK2 | Pigeonpox virus | fep_249 | AID46737 | ANK18 |
| Skunkpox virus | SKPV-WA-181 | AOP31660 | ANK2 | Flamingopox virus | fgpv_268 | AUD40353 | ANK18 |
| Volepox virus | VPXV-CA-181 | AOP31871 | ANK2 | Mudlarkpox virus | ankyrin | QRM15286 | ANK18 |
| Akhmeta virus | AKMV-88-193 | AXN74978 | ANK2 | Penguinpox virus | ankyrin | QRM15638 | ANK18 |
| Alaskapox virus | ankyrin | QED21126 | ANK2 | Albatrosspox virus | ankyrin | QRM15968 | ANK18 |
| Abatino macacapox virus | KM543_gp182 | YP_010085850 | ANK2 | Canarypox virus | CNPV026 | AAR83372 | ANK19 |
| Variola virus | B6R | CAA49115 | ANK2 | Pigeonpox virus | fep_013 | AID46529 | ANK19 |
| Tanapox virus | 11L | CAC21249 | ANK3 | Flamingopox virus | fgpv_002 | AUD40109 | ANK19 |
| Yaba monkey tumor virus | 11L | AAR07370 | ANK3 | Flamingopox virus | fgpv_005 | AUD40112 | ANK19 |
| Eptesipox virus | EPTV-WA-011 | ASK51212 | ANK3 | Mudlarkpox virus | ankyrin | QRM15302 | ANK19 |
| Eptesipox virus | EPTV-WA-168 | ASK51369 | ANK3 | Penguinpox virus | ankyrin | QRM15655 | ANK19 |
| Eptesipox virus | EPTV-WA-179 | ASK51380 | ANK3 | Albatrosspox virus | ankyrin | QRM15985 | ANK19 |
| Eptesipox virus | EPTV-WA-181 | ASK51382 | ANK3 | Magpiepox virus | MPPV-028 | QZW33301 | ANK19 |
| White-tailed deer poxvirus | DPV019 | AUI80581 | ANK3 | Canarypox virus | CNPV004 | AAR83350 | ANK20 |
| Moosepox virus GoldyGopher4 | ankyrin | AYC44729 | ANK3 | Canarypox virus | CNPV233 | AAR83579 | ANK20 |
| Lumpy skin disease virus | LSDV012 | AAK84973 | ANK4 | Canarypox virus | CNPV234 | AAR83580 | ANK20 |
| Tanapox virus | 8L | CAC21246 | ANK4 | Canarypox virus | CNPV325 | AAR83671 | ANK20 |
| Deerpox virus | DpV83gp014 | ABI99171 | ANK4 | Flamingopox virus | fgpv_117 | AUD40219 | ANK20 |
| Pteropox virus | PTPV-Aus-008 | ANS71092 | ANK4 | Mudlarkpox virus | ankyrin | QRM15512 | ANK20 |
| Eptesipox virus | EPTV-WA-169 | ASK51370 | ANK4 | Mudlarkpox virus | ankyrin | QRM15513 | ANK20 |
| White-tailed deer poxvirus | DPV014 | AUI80576 | ANK4 | Penguinpox virus | ankyrin | QRM15865 | ANK20 |
| Moosepox virus GoldyGopher4 | ankyrin | AYC44724 | ANK4 | Penguinpox virus | ankyrin | QRM15866 | ANK20 |
| Sea otter poxvirus | SOPV-ELK-006 | AWU47051 | ANK4 | Albatrosspox virus | ankyrin | QRM16202 | ANK20 |
| Sheeppox virus | SPPV_10 | NP_659586 | ANK4 | Albatrosspox virus | ankyrin | QRM16203 | ANK20 |
| Goatpox virus | GTPV_gp010 | YP_001293204 | ANK4 | Magpiepox virus | MPPV-242 | QZW33555 | ANK20 |
| Rabbit fibroma virus | s149R | AAF18027 | ANK5 | Fowlpox virus | FPV023 | AAF44367 | ANK21 |
| Myxoma virus | m149R | AAF15036 | ANK5 | Fowlpox virus | FPV233 | AAF44577 | ANK21 |
| Myxoma virus | m150R | AAF15037 | ANK5 | Canarypox virus | CNPV041 | AAR83387 | ANK21 |
| Lumpy skin disease virus | LSDV147 | AAK85108 | ANK5 | Canarypox virus | CNPV301 | AAR83647 | ANK21 |
| Swinepox virus | SPV142 | AAL69881 | ANK5 | Canarypox virus | CNPV304 | AAR83650 | ANK21 |
| Swinepox virus | SPV144 | AAL69883 | ANK5 | Pigeonpox virus | fep_027 | AID46543 | ANK21 |
| Tanapox virus | 146R | CAC21384 | ANK5 | Pigeonpox virus | fep_239 | AID46730 | ANK21 |
| Tanapox virus | 148R | CAC21386 | ANK5 | Flamingopox virus | fgpv_020 | AUD40125 | ANK21 |
| Yaba monkey tumor virus | 146R | AAR07498 | ANK5 | Flamingopox virus | fgpv_252 | AUD40340 | ANK21 |
| Yokapox virus | YKV176 | AEN03765 | ANK5 | Mudlarkpox virus | ankyrin | QRM15317 | ANK21 |
| Yokapox virus | YKV183c | AEN03772 | ANK5 | Mudlarkpox virus | ankyrin | QRM15580 | ANK21 |
| Yokapox virus | YKV184 | AEN03773 | ANK5 | Mudlarkpox virus | ankyrin | QRM15583 | ANK21 |
| BeAn 58058 virus | BAV00015 | APG58205 | ANK5 | Penguinpox virus | ankyrin | QRM15671 | ANK21 |
| BeAn 58058 virus | BAV00023 | APG58212 | ANK5 | Penguinpox virus | ankyrin | QRM15933 | ANK21 |
| BeAn 58058 virus | BAV00038 | APG58226 | ANK5 | Penguinpox virus | ankyrin | QRM15936 | ANK21 |
| BeAn 58058 virus | BAV00200 | APG58376 | ANK5 | Albatrosspox virus | ankyrin | QRM16001 | ANK21 |
| Mudlarkpox virus | Ig-like | QRM15600 | ANK5 | Albatrosspox virus | ankyrin | QRM16270 | ANK21 |
| Magpiepox virus | MPPV-008 | QZW33275 | ANK5 | Albatrosspox virus | ankyrin | QRM16273 | ANK21 |
| Sheeppox virus | SPPV_140 | NP_659717 | ANK5 | Magpiepox virus | MPPV-044 | QZW33321 | ANK21 |
| Goatpox virus | GTPV_gp140 | YP_001293338 | ANK5 | Magpiepox virus | MPPV-324 | QZW33654 | ANK21 |
| Cotia virus | COTV016 | YP_005296204 | ANK5 | Magpiepox virus | MPPV-327 | QZW33658 | ANK21 |
| Cotia virus | COTV019 | YP_005296207 | ANK5 | Fowlpox virus | FPV034 | AAF44378 | ANK22 |
| Cotia virus | COTV162 | YP_005296350 | ANK5 | Fowlpox virus | FPV232 | AAF44576 | ANK22 |
| Cotia virus | COTV164 | YP_005296352 | ANK5 | Pigeonpox virus | fep_235 | AID46727 | ANK22 |
| Monkeypox virus | D9L | AAL40470 | ANK6 | Pigeonpox virus | fep_238 | AID46729 | ANK22 |
| Cowpox virus | CPXV027 | AAM13474 | ANK6 | Turkeypox virus | ankyrin | ALA62477 | ANK22 |
| Vaccinia virus | VACWR019 | AAO89298 | ANK6 | Turkeypox virus | ankyrin | ALA62538 | ANK22 |
| Taterapox virus | TATV_DAH68_020 | ABD97586 | ANK6 | Flamingopox virus | fgpv_033 | AUD40138 | ANK22 |
| Raccoonpox virus | RCNV-Herman-015 | AKJ93649 | ANK6 | Flamingopox virus | fgpv_247 | AUD40335 | ANK22 |
| Skunkpox virus | SKPV-WA-018 | AOP31497 | ANK6 | Flamingopox virus | fgpv_251 | AUD40339 | ANK22 |
| Volepox virus | VPXV-CA-018 | AOP31708 | ANK6 | Fowlpox virus | FPV018 | AAF44362 | ANK23 |
| NY_014 poxvirus | NY_014-196 | AST09597 | ANK6 | Fowlpox virus | FPV242 | AAF44586 | ANK23 |
| Akhmeta virus | AKMV-88-023 | AXN74808 | ANK6 | Fowlpox virus | FPV243 | AAF44587 | ANK23 |
| Alaskapox virus | ankyrin-like | QED21117 | ANK6 | Canarypox virus | CNPV007 | AAR83353 | ANK23 |
| Cotia virus | COTV023 | YP_005296211 | ANK6 | Canarypox virus | CNPV027 | AAR83373 | ANK23 |
| Abatino macacapox virus | KM543_gp020 | YP_010085688 | ANK6 | Canarypox virus | CNPV034 | AAR83380 | ANK23 |
| Monkeypox virus | D1L | AAL40462 | ANK7 | Canarypox virus | CNPV314 | AAR83660 | ANK23 |
| Monkeypox virus | N4R | AAL40646 | ANK7 | Canarypox virus | CNPV322 | AAR83668 | ANK23 |
| Cowpox virus | CPXV017 | AAM13464 | ANK7 | Pigeonpox virus | fep_021 | AID46537 | ANK23 |
| Yokapox virus | YKV172 | AEN03761 | ANK7 | Pigeonpox virus | fep_246 | AID46735 | ANK23 |
| Raccoonpox virus | RCNV-Herman-012 | AKJ93646 | ANK7 | Flamingopox virus | fgpv_013 | AUD40120 | ANK23 |
| Raccoonpox virus | RCNV-Herman-197 | AKJ93830 | ANK7 | Flamingopox virus | fgpv_265 | AUD40350 | ANK23 |
| Skunkpox virus | SKPV-WA-201 | AOP31680 | ANK7 | Mudlarkpox virus | ankyrin | QRM15303 | ANK23 |
| Volepox virus | VPXV-CA-009 | AOP31699 | ANK7 | Mudlarkpox virus | ankyrin | QRM15310 | ANK23 |
| Murmansk poxvirus | Murmansk-194 | AST09389 | ANK7 | Mudlarkpox virus | ankyrin | QRM15593 | ANK23 |
| NY_014 poxvirus | NY_014-006 | AST09407 | ANK7 | Mudlarkpox virus | ankyrin | QRM15601 | ANK23 |
| Akhmeta virus | AKMV-88-013 | AXN74798 | ANK7 | Penguinpox virus | ankyrin | QRM15636 | ANK23 |
| Alaskapox virus | ankyrin-like | QED21124 | ANK7 | Penguinpox virus | ankyrin | QRM15656 | ANK23 |
| Alaskapox virus | ankyrin | QED21138 | ANK7 | Penguinpox virus | ankyrin | QRM15664 | ANK23 |
| Abatino macacapox virus | KM543_gp010 | YP_010085678 | ANK7 | Penguinpox virus | ankyrin | QRM15946 | ANK23 |
| Monkeypox virus | D7L | AAL40468 | ANK8 | Penguinpox virus | ankyrin | QRM15954 | ANK23 |
| Camelpox virus | ankyrin | AAL73711 | ANK8 | Albatrosspox virus | c-type | QRM15966 | ANK23 |
| Camelpox virus | ankyrin | AAL73915 | ANK8 | Albatrosspox virus | ankyrin | QRM15986 | ANK23 |
| Cowpox virus | CPXV008 | AAM13454 | ANK8 | Albatrosspox virus | ankyrin | QRM15994 | ANK23 |
| Cowpox virus | VHR1 | AAM13472 | ANK8 | Albatrosspox virus | ankyrin | QRM16283 | ANK23 |
| Cowpox virus | CPXV223 | AAM13661 | ANK8 | Albatrosspox virus | ankyrin | QRM16291 | ANK23 |
| Vaccinia virus | VACWR014 | AAO89293 | ANK8 | Magpiepox virus | MPPV-029 | QZW33302 | ANK23 |
| Taterapox virus | TATV_DAH68_018 | ABD97584 | ANK8 | Magpiepox virus | MPPV-339 | QZW33668 | ANK23 |
| Raccoonpox virus | RCNV-Herman-004 | AKJ93638 | ANK8 | Fowlpox virus | FPV031 | AAF44375 | ANK24 |
| Raccoonpox virus | RCNV-Herman-013 | AKJ93647 | ANK8 | Canarypox virus | CNPV050 | AAR83396 | ANK24 |
| Raccoonpox virus | RCNV-Herman-205 | AKJ93838 | ANK8 | Pigeonpox virus | fep_036 | AID46550 | ANK24 |
| Skunkpox virus | SKPV-WA-005 | AOP31484 | ANK8 | Pigeonpox virus | fep_247 | AID46736 | ANK24 |
| Skunkpox virus | SKPV-WA-016 | AOP31495 | ANK8 | Turkeypox virus | ankyrin | ALA62387 | ANK24 |
| Skunkpox virus | SKPV-WA-208 | AOP31687 | ANK8 | Flamingopox virus | fgpv_030 | AUD40135 | ANK24 |
| Volepox virus | VPXV-CA-005 | AOP31695 | ANK8 | Flamingopox virus | fgpv_266 | AUD40351 | ANK24 |
| Volepox virus | VPXV-CA-016 | AOP31706 | ANK8 | Mudlarkpox virus | ankyrin | QRM15327 | ANK24 |
| Volepox virus | VPXV-CA-203 | AOP31893 | ANK8 | Penguinpox virus | ankyrin | QRM15680 | ANK24 |
| Eptesipox virus | EPTV-WA-010 | ASK51211 | ANK8 | Albatrosspox virus | ankyrin | QRM16010 | ANK24 |
| Eptesipox virus | EPTV-WA-182 | ASK51383 | ANK8 | Magpiepox virus | MPPV-055 | QZW33333 | ANK24 |
| Murmansk poxvirus | Murmansk-017 | AST09212 | ANK8 | Fowlpox virus | FPV115 | AAF44459 | ANK25 |
| Murmansk poxvirus | Murmansk-197 | AST09392 | ANK8 | Fowlpox virus | FPV223 | AAF44567 | ANK25 |
| NY_014 poxvirus | NY_014-187 | AST09588 | ANK8 | Fowlpox virus | FPV224 | AAF44568 | ANK25 |
| Akhmeta virus | AKMV-88-005 | AXN74790 | ANK8 | Fowlpox virus | FPV228 | AAF44572 | ANK25 |
| Akhmeta virus | AKMV-88-021 | AXN74806 | ANK8 | Fowlpox virus | FPV234 | AAF44578 | ANK25 |
| Akhmeta virus | AKMV-88-216 | AXN75001 | ANK8 | Canarypox virus | CNPV237 | AAR83583 | ANK25 |
| Alaskapox virus | ankyrin | QED21110 | ANK8 | Canarypox virus | CNPV298 | AAR83644 | ANK25 |
| Alaskapox virus | ankyrin | QED21111 | ANK8 | Deerpox virus | DpV83gp019 | ABI99176 | ANK25 |
| Abatino macacapox virus | KM543_gp018 | YP_010085686 | ANK8 | Yokapox virus | YKV169 | AEN03758 | ANK25 |
| Abatino macacapox virus | KM543_gp205 | YP_010085873 | ANK8 | Turkeypox virus | ankyrin | ALA62378 | ANK25 |
| Variola virus | D6L | CAA48947 | ANK8 | Turkeypox virus | ankyrin | ALA62382 | ANK25 |
| Rabbit fibroma virus | s005L | AAF17888 | ANK9 | NY_014 poxvirus | NY_014-184 | AST09585 | ANK25 |
| Rabbit fibroma virus | s148R | AAF18026 | ANK9 | Flamingopox virus | fgpv_253 | AUD40341 | ANK25 |
| Rabbit fibroma virus | s005R | AAF18037 | ANK9 | Mudlarkpox virus | ankyrin | QRM15516 | ANK25 |
| Myxoma virus | m005R | AAF14892 | ANK9 | Mudlarkpox virus | ankyrin | QRM15577 | ANK25 |
| Myxoma virus | m148R | AAF15035 | ANK9 | Penguinpox virus | ankyrin | QRM15869 | ANK25 |
| Myxoma virus | m005L | AAF15051 | ANK9 | Penguinpox virus | ankyrin | QRM15930 | ANK25 |
| Lumpy skin disease virus | LSDV145 | AAK85106 | ANK9 | Albatrosspox virus | ankyrin | QRM16206 | ANK25 |
| Lumpy skin disease virus | LSDV148 | AAK85109 | ANK9 | Albatrosspox virus | ankyrin | QRM16267 | ANK25 |
| Lumpy skin disease virus | LSDV152 | AAK85113 | ANK9 | Magpiepox virus | MPPV-246 | QZW33565 | ANK25 |
| Swinepox virus | SPV141 | AAL69880 | ANK9 | Magpiepox virus | MPPV-310 | QZW33636 | ANK25 |
| Swinepox virus | SPV143 | AAL69882 | ANK9 | Magpiepox virus | MPPV-316 | QZW33645 | ANK25 |
| Tanapox virus | 147R | CAC21385 | ANK9 | Cotia virus | COTV031 | YP_005296219 | ANK25 |
| Yaba monkey tumor virus | 147R | AAR07499 | ANK9 | Fowlpox virus | FPV014 | AAF44358 | ANK26 |
| Deerpox virus | DpV83gp161 | ABI99316 | ANK9 | Fowlpox virus | FPV219 | AAF44563 | ANK26 |
| Deerpox virus | DpV83gp164 | ABI99319 | ANK9 | Canarypox virus | CNPV017 | AAR83363 | ANK26 |
| Deerpox virus | DpV83gp165 | ABI99320 | ANK9 | Canarypox virus | CNPV019 | AAR83365 | ANK26 |
| Deerpox virus | DpV83gp166 | ABI99321 | ANK9 | Canarypox virus | CNPV229 | AAR83575 | ANK26 |
| BeAn 58058 virus | BAV00191 | APG58367 | ANK9 | Canarypox virus | CNPV296 | AAR83642 | ANK26 |
| White-tailed deer poxvirus | DPV161 | AUI80722 | ANK9 | Pigeonpox virus | fep_017 | AID46533 | ANK26 |
| White-tailed deer poxvirus | DPV164 | AUI80724 | ANK9 | Pigeonpox virus | fep_226 | AID46722 | ANK26 |
| White-tailed deer poxvirus | DPV165 | AUI80725 | ANK9 | Turkeypox virus | ankyrin | ALA62535 | ANK26 |
| White-tailed deer poxvirus | DPV166 | AUI80726 | ANK9 | Turkeypox virus | ankyrin | ALA62540 | ANK26 |
| Moosepox virus GoldyGopher4 | ankyrin | AYC44700 | ANK9 | Flamingopox virus | fgpv_001 | AUD40108 | ANK26 |
| Moosepox virus GoldyGopher4 | ankyrin | AYC44702 | ANK9 | Flamingopox virus | fgpv_009 | AUD40116 | ANK26 |
| Moosepox virus GoldyGopher4 | ankyrin | AYC44703 | ANK9 | Flamingopox virus | fgpv_238 | AUD40329 | ANK26 |
| Moosepox virus GoldyGopher4 | ankyrin | AYC44704 | ANK9 | Mudlarkpox virus | ankyrin | QRM15293 | ANK26 |
| Sheeppox virus | SPPV_138 | NP_659715 | ANK9 | Mudlarkpox virus | ankyrin | QRM15295 | ANK26 |
| Sheeppox virus | SPPV_141 | NP_659718 | ANK9 | Mudlarkpox virus | ankyrin | QRM15508 | ANK26 |
| Sheeppox virus | SPPV_145 | NP_659722 | ANK9 | Mudlarkpox virus | ankyrin | QRM15575 | ANK26 |
| Goatpox virus | GTPV_gp138 | YP_001293336 | ANK9 | Penguinpox virus | ankyrin | QRM15646 | ANK26 |
| Goatpox virus | GTPV_gp141.2 | YP_001293339 | ANK9 | Penguinpox virus | ankyrin | QRM15648 | ANK26 |
| Goatpox virus | GTPV_gp145 | YP_001293343 | ANK9 | Penguinpox virus | ankyrin | QRM15861 | ANK26 |
| Cotia virus | COTV158 | YP_005296346 | ANK9 | Penguinpox virus | ankyrin | QRM15928 | ANK26 |
| Ectromelia virus | EVM005 | AAM92311 | ANK10 | Albatrosspox virus | ankyrin | QRM15976 | ANK26 |
| Monkeypox virus | B17R | AAL40636 | ANK10 | Albatrosspox virus | ankyrin | QRM15978 | ANK26 |
| Camelpox virus | ankyrin | AAL73909 | ANK10 | Albatrosspox virus | ankyrin | QRM16198 | ANK26 |
| Cowpox virus | CPXV011 | AAM13458 | ANK10 | Albatrosspox virus | ankyrin | QRM16265 | ANK26 |
| Cowpox virus | CPXV213 | AAM13652 | ANK10 | Magpiepox virus | MPPV-017 | QZW33286 | ANK26 |
| Vaccinia virus | VACWR203 | AAO89482 | ANK10 | Magpiepox virus | MPPV-020 | QZW33288 | ANK26 |
| Raccoonpox virus | RCNV-Herman-192 | AKJ93825 | ANK10 | Magpiepox virus | MPPV-158 | QZW33454 | ANK26 |
| Skunkpox virus | SKPV-WA-196 | AOP31675 | ANK10 | Magpiepox virus | MPPV-239 | QZW33549 | ANK26 |
| Volepox virus | VPXV-CA-196 | AOP31886 | ANK10 | Magpiepox virus | MPPV-243 | QZW33558 | ANK26 |
| Murmansk poxvirus | Murmansk-190 | AST09385 | ANK10 | Magpiepox virus | MPPV-308 | QZW33634 | ANK26 |
| NY_014 poxvirus | NY_014-182 | AST09583 | ANK10 | Fowlpox virus | FPV241 | AAF44585 | ANK27 |
| Akhmeta virus | AKMV-88-008 | AXN74793 | ANK10 | Canarypox virus | CNPV309 | AAR83655 | ANK27 |
| Akhmeta virus | AKMV-88-208 | AXN74993 | ANK10 | Flamingopox virus | fgpv_260 | AUD40348 | ANK27 |
| Alaskapox virus | ankyrin-like | QED21104 | ANK10 | Mudlarkpox virus | ankyrin | QRM15588 | ANK27 |
| Abatino macacapox virus | KM543_gp004 | YP_010085672 | ANK10 | Penguinpox virus | ankyrin | QRM15941 | ANK27 |
| Abatino macacapox virus | KM543_gp197 | YP_010085865 | ANK10 | Albatrosspox virus | ankyrin | QRM16278 | ANK27 |
| Variola virus | B21R | CAA49130 | ANK10 | Magpiepox virus | MPPV-334 | QZW33663 | ANK27 |
| Ectromelia virus | K1R | AAC99576 | ANK11 | Fowlpox virus | FPV240 | AAF44584 | ANK28 |
| Ectromelia virus | EVM002 | AAM92308 | ANK11 | Canarypox virus | CNPV028 | AAR83374 | ANK28 |
| Monkeypox virus | J3L | AAL40461 | ANK11 | Canarypox virus | CNPV151 | AAR83497 | ANK28 |
| Monkeypox virus | J1R | AAL40647 | ANK11 | Canarypox virus | CNPV308 | AAR83654 | ANK28 |
| Camelpox virus | ankyrin | AAL73916 | ANK11 | Pigeonpox virus | fep_016 | AID46532 | ANK28 |
| Camelpox virus | ankyrin | AAL73921 | ANK11 | Pigeonpox virus | fep_244 | AID46733 | ANK28 |
| Cowpox virus | CPXV006 | AAM13452 | ANK11 | Flamingopox virus | fgpv_008 | AUD40115 | ANK28 |
| Cowpox virus | CPXV220 | AAM13658 | ANK11 | Flamingopox virus | fgpv_259 | AUD40347 | ANK28 |
| Cowpox virus | CPXV225 | AAM13662 | ANK11 | Mudlarkpox virus | ankyrin | QRM15304 | ANK28 |
| Vaccinia virus | VACWR008 | AAO89287 | ANK11 | Mudlarkpox virus | ankyrin | QRM15428 | ANK28 |
| Vaccinia virus | VACWR211 | AAO89490 | ANK11 | Mudlarkpox virus | ankyrin | QRM15587 | ANK28 |
| Taterapox virus | TATV_DAH68_006 | ABD97572 | ANK11 | Penguinpox virus | ankyrin | QRM15657 | ANK28 |
| Taterapox virus | TATV_DAH68_220 | ABD97786 | ANK11 | Penguinpox virus | ankyrin | QRM15784 | ANK28 |
| Yokapox virus | YKV179 | AEN03768 | ANK11 | Penguinpox virus | ankyrin | QRM15940 | ANK28 |
| Raccoonpox virus | RCNV-Herman-003 | AKJ93637 | ANK11 | Albatrosspox virus | ankyrin | QRM15987 | ANK28 |
| Raccoonpox virus | RCNV-Herman-206 | AKJ93839 | ANK11 | Albatrosspox virus | ankyrin | QRM16116 | ANK28 |
| Skunkpox virus | SKPV-WA-209 | AOP31688 | ANK11 | Albatrosspox virus | ankyrin | QRM16277 | ANK28 |
| Volepox virus | VPXV-CA-204 | AOP31894 | ANK11 | Magpiepox virus | MPPV-030 | QZW33303 | ANK28 |
| Murmansk poxvirus | Murmansk-003 | AST09198 | ANK11 | Magpiepox virus | MPPV-160 | QZW33459 | ANK28 |
| Murmansk poxvirus | Murmansk-009 | AST09204 | ANK11 | Magpiepox virus | MPPV-332 | QZW33662 | ANK28 |
| Murmansk poxvirus | Murmansk-204 | AST09399 | ANK11 | Fowlpox virus | FPV012 | AAF44356 | ANK29 |
| NY_014 poxvirus | NY_014-002 | AST09403 | ANK11 | Canarypox virus | CNPV030 | AAR83376 | ANK29 |
| NY_014 poxvirus | NY_014-009 | AST09410 | ANK11 | Pigeonpox virus | fep_015 | AID46531 | ANK29 |
| NY_014 poxvirus | NY_014-193 | AST09594 | ANK11 | Flamingopox virus | fgpv_007 | AUD40114 | ANK29 |
| Akhmeta virus | AKMV-88-003 | AXN74788 | ANK11 | Mudlarkpox virus | ankyrin | QRM15306 | ANK29 |
| Akhmeta virus | AKMV-88-213 | AXN74998 | ANK11 | Penguinpox virus | ankyrin | QRM15659 | ANK29 |
| Akhmeta virus | AKMV-88-218 | AXN75003 | ANK11 | Albatrosspox virus | ankyrin | QRM15989 | ANK29 |
| Alaskapox virus | ankyrin-like | QED21119 | ANK11 | Magpiepox virus | MPPV-032 | QZW33306 | ANK29 |
| Abatino macacapox virus | KM543_gp202 | YP_010085870 | ANK11 | Canarypox virus | CNPV044 | AAR83390 | ANK30 |
| Abatino macacapox virus | KM543_gp206 | YP_010085874 | ANK11 | Canarypox virus | CNPV046 | AAR83392 | ANK30 |
| Variola virus | G3R | CAA49136 | ANK11 | Pigeonpox virus | fep_030 | AID46546 | ANK30 |
| Ectromelia virus | C11R | AAC99570 | ANK12 | Pigeonpox virus | fep_033 | AID46547 | ANK30 |
| Camelpox virus | truncated | AAL73906 | ANK12 | Turkeypox virus | ankyrin | ALA62537 | ANK30 |
| Cowpox virus | CPXV211 | AAM13650 | ANK12 | Flamingopox virus | fgpv_023 | AUD40128 | ANK30 |
| Vaccinia virus | VACWR199 | AAO89478 | ANK12 | Flamingopox virus | fgpv_027 | AUD40132 | ANK30 |
| Taterapox virus | TATV_DAH68_205 | ABD97771 | ANK12 | Mudlarkpox virus | ankyrin | QRM15320 | ANK30 |
| Taterapox virus | TATV_DAH68_208 | ABD97774 | ANK12 | Mudlarkpox virus | ankyrin | QRM15323 | ANK30 |
| Raccoonpox virus | RCNV-Herman-190 | AKJ93823 | ANK12 | Penguinpox virus | ankyrin | QRM15674 | ANK30 |
| Skunkpox virus | SKPV-WA-194 | AOP31673 | ANK12 | Penguinpox virus | ankyrin | QRM15676 | ANK30 |
| Volepox virus | VPXV-CA-194 | AOP31884 | ANK12 | Albatrosspox virus | ankyrin | QRM16004 | ANK30 |
| Akhmeta virus | AKMV-88-206 | AXN74991 | ANK12 | Albatrosspox virus | ankyrin | QRM16006 | ANK30 |
| Alaskapox virus | ankyrin | QED21120 | ANK12 | Magpiepox virus | MPPV-047 | QZW33324 | ANK30 |
| Abatino macacapox virus | KM543_gp195 | YP_010085863 | ANK12 | Magpiepox virus | MPPV-049 | QZW33327 | ANK30 |
| Variola virus | B19R | CAA49128 | ANK12 | Magpiepox virus | MPPV-243 | QZW33557 | ANK30 |
| Ectromelia virus | EVM010 | AAM92316 | ANK13 | Fowlpox virus | FPV022 | AAF44366 | ANK31 |
| Cowpox virus | CPXV016 | AAM13463 | ANK13 | Fowlpox virus | FPV024 | AAF44368 | ANK31 |
| Cowpox virus | CPXV019 | AAM13466 | ANK13 | Canarypox virus | CNPV040 | AAR83386 | ANK31 |
| Raccoonpox virus | RCNV-Herman-011 | AKJ93645 | ANK13 | Canarypox virus | CNPV042 | AAR83388 | ANK31 |
| Raccoonpox virus | RCNV-Herman-198 | AKJ93831 | ANK13 | Pigeonpox virus | fep_026 | AID46542 | ANK31 |
| Skunkpox virus | SKPV-WA-009 | AOP31488 | ANK13 | Pigeonpox virus | fep_028 | AID46544 | ANK31 |
| Skunkpox virus | SKPV-WA-011 | AOP31490 | ANK13 | Turkeypox virus | ankyrin | ALA62381 | ANK31 |
| Skunkpox virus | SKPV-WA-202 | AOP31681 | ANK13 | Turkeypox virus | ankyrin | ALA62383 | ANK31 |
| Volepox virus | VPXV-CA-008 | AOP31698 | ANK13 | Flamingopox virus | fgpv_019 | AUD40124 | ANK31 |
| Volepox virus | VPXV-CA-011 | AOP31701 | ANK13 | Flamingopox virus | fgpv_021 | AUD40126 | ANK31 |
| NY_014 poxvirus | NY_014-005 | AST09406 | ANK13 | Mudlarkpox virus | ankyrin | QRM15316 | ANK31 |
| Akhmeta virus | AKMV-88-012 | AXN74797 | ANK13 | Mudlarkpox virus | ankyrin | QRM15318 | ANK31 |
| Akhmeta virus | AKMV-88-015 | AXN74800 | ANK13 | Penguinpox virus | ankyrin | QRM15670 | ANK31 |
| Alaskapox virus | ankyrin | QED21106 | ANK13 | Penguinpox virus | ankyrin | QRM15672 | ANK31 |
| Alaskapox virus | ankyrin | QED21123 | ANK13 | Albatrosspox virus | ankyrin | QRM16000 | ANK31 |
| Abatino macacapox virus | KM543_gp009 | YP_010085677 | ANK13 | Albatrosspox virus | ankyrin | QRM16002 | ANK31 |
| Abatino macacapox virus | KM543_gp012 | YP_010085680 | ANK13 | Magpiepox virus | MPPV-043 | QZW33319 | ANK31 |
| Orf virus | ORF008 | AAR98233 | ANK14 | Magpiepox virus | MPPV-045 | QZW33322 | ANK31 |
| Orf virus | ORF123 | AAR98348 | ANK14 | Fowlpox virus | FPV230 | AAF44574 | ANK32 |
| Orf virus | ORF126 | AAR98351 | ANK14 | Fowlpox virus | FPV231 | AAF44575 | ANK32 |
| Orf virus | ORF128 | AAR98353 | ANK14 | Canarypox virus | CNPV010 | AAR83356 | ANK32 |
| Orf virus | ORF129 | AAR98354 | ANK14 | Canarypox virus | CNPV015 | AAR83361 | ANK32 |
| Bovine papular stomatitis virus | ORF003 | AAR98360 | ANK14 | Canarypox virus | CNPV303 | AAR83649 | ANK32 |
| Bovine papular stomatitis virus | ORF004 | AAR98361 | ANK14 | Canarypox virus | CNPV318 | AAR83664 | ANK32 |
| Bovine papular stomatitis virus | ORF008 | AAR98365 | ANK14 | Pigeonpox virus | fep_008 | AID46525 | ANK32 |
| Bovine papular stomatitis virus | ORF123 | AAR98479 | ANK14 | Flamingopox virus | fgpv_250 | AUD40338 | ANK32 |
| Bovine papular stomatitis virus | ORF126 | AAR98482 | ANK14 | Flamingopox virus | fgpv_282 | AUD40360 | ANK32 |
| Bovine papular stomatitis virus | ORF128 | AAR98484 | ANK14 | Mudlarkpox virus | ankyrin | QRM15291 | ANK32 |
| Bovine papular stomatitis virus | ORF129 | AAR98485 | ANK14 | Mudlarkpox virus | ankyrin | QRM15582 | ANK32 |
| Pseudocowpox virus | Ankyrin/F-box | ADC53905 | ANK14 | Mudlarkpox virus | ankyrin | QRM15597 | ANK32 |
| Pseudocowpox virus | Ankyrin/F-box | ADC53906 | ANK14 | Penguinpox virus | ankyrin | QRM15639 | ANK32 |
| Pseudocowpox virus | Ankyrin/F-box | ADC53907 | ANK14 | Penguinpox virus | ankyrin | QRM15644 | ANK32 |
| Pseudocowpox virus | Ankyrin/F-box | ADC54023 | ANK14 | Penguinpox virus | ankyrin | QRM15935 | ANK32 |
| Pseudocowpox virus | Ankyrin/F-box | ADC54026 | ANK14 | Penguinpox virus | ankyrin | QRM15950 | ANK32 |
| Pseudocowpox virus | Ankyrin/F-box | ADC54027 | ANK14 | Albatrosspox virus | ankyrin | QRM15969 | ANK32 |
| Pseudocowpox virus | Ankyrin/F-box | ADC54028 | ANK14 | Albatrosspox virus | ankyrin | QRM15974 | ANK32 |
| Parapoxvirus red deer/HL953 | putative | AIZ77254 | ANK14 | Albatrosspox virus | ankyrin | QRM16272 | ANK32 |
| Parapoxvirus red deer/HL953 | putative | AIZ77255 | ANK14 | Albatrosspox virus | ankyrin | QRM16287 | ANK32 |
| Parapoxvirus red deer/HL953 | putative | AIZ77258 | ANK14 | Magpiepox virus | MPPV-015 | QZW33284 | ANK32 |
| Parapoxvirus red deer/HL953 | putative | AIZ77374 | ANK14 | Magpiepox virus | MPPV-326 | QZW33657 | ANK32 |
| Parapoxvirus red deer/HL953 | putative | AIZ77377 | ANK14 | Fowlpox virus | FPV218 | AAF44562 | ANK33 |
| Parapoxvirus red deer/HL953 | putative | AIZ77379 | ANK14 | Fowlpox virus | FPV245 | AAF44589 | ANK33 |
| Parapoxvirus red deer/HL953 | putative | AIZ77380 | ANK14 | Canarypox virus | CNPV020 | AAR83366 | ANK33 |
| BeAn 58058 virus | BAV00005 | APG58195 | ANK14 | Canarypox virus | CNPV295 | AAR83641 | ANK33 |
| BeAn 58058 virus | BAV00227 | APG58399 | ANK14 | Turkeypox virus | ankyrin | ALA62531 | ANK33 |
| Grey sealpox virus | SePPVgORF119 | ASC55520 | ANK14 | Flamingopox virus | fgpv_237 | AUD40328 | ANK33 |
| Grey sealpox virus | SePPVgORF111 | ASC55527 | ANK14 | Flamingopox virus | fgpv_269 | AUD40354 | ANK33 |
| Grey sealpox virus | SePPVgORF115 | ASC55621 | ANK14 | Mudlarkpox virus | ankyrin | QRM15296 | ANK33 |
| Grey sealpox virus | SePPVgORF002 | ASC55623 | ANK14 | Mudlarkpox virus | ankyrin | QRM15574 | ANK33 |
| Cotia virus | COTV002 | YP_005296190 | ANK14 | Penguinpox virus | ankyrin | QRM15649 | ANK33 |
| Cotia virus | COTV005 | YP_005296193 | ANK14 | Penguinpox virus | ankyrin | QRM15927 | ANK33 |
| Cotia virus | COTV181 | YP_005296369 | ANK14 | Albatrosspox virus | ankyrin | QRM15979 | ANK33 |
| Cotia virus | COTV184 | YP_005296372 | ANK14 | Albatrosspox virus | ankyrin | QRM16264 | ANK33 |
| Equine parapoxvirus | ORF008 | WOC29259 | ANK14 | Magpiepox virus | MPPV-021 | QZW33290 | ANK33 |
| Fowlpox virus | FPV216 | AAF44560 | ANK15 | Magpiepox virus | MPPV-307 | QZW33633 | ANK33 |
| Canarypox virus | CNPV185 | AAR83531 | ANK15 | Fowlpox virus | FPV222 | AAF44566 | ANK34 |
| Canarypox virus | CNPV293 | AAR83639 | ANK15 | Canarypox virus | CNPV297 | AAR83643 | ANK34 |
| Pigeonpox virus | fep_222 | AID46720 | ANK15 | Pigeonpox virus | fep_228 | AID46724 | ANK34 |
| Flamingopox virus | fgpv_233 | AUD40326 | ANK15 | Flamingopox virus | fgpv_241 | AUD40332 | ANK34 |
| Mudlarkpox virus | ankyrin | QRM15461 | ANK15 | Mudlarkpox virus | ankyrin | QRM15576 | ANK34 |
| Mudlarkpox virus | ankyrin | QRM15572 | ANK15 | Penguinpox virus | ankyrin | QRM15929 | ANK34 |
| Penguinpox virus | ankyrin | QRM15816 | ANK15 | Albatrosspox virus | ankyrin | QRM16266 | ANK34 |
| Penguinpox virus | ankyrin | QRM15925 | ANK15 | Fowlpox virus | FPV026 | AAF44370 | ANK35 |
| Albatrosspox virus | ankyrin | QRM16151 | ANK15 | Vaccinia virus | VACWR017 | AAO89296 | ANK35 |
| Albatrosspox virus | ankyrin | QRM16262 | ANK15 | Canarypox virus | CNPV294 | AAR83640 | ANK35 |
| Magpiepox virus | MPPV-304 | QZW33630 | ANK15 | Turkeypox virus | ankyrin | ALA62384 | ANK35 |
| Fowlpox virus | FPV162 | AAF44506 | ANK16 | Flamingopox virus | fgpv_024 | AUD40129 | ANK35 |
| Canarypox virus | CNPV143 | AAR83489 | ANK16 | Mudlarkpox virus | ankyrin | QRM15573 | ANK35 |
| Canarypox virus | CNPV223 | AAR83569 | ANK16 | Penguinpox virus | ankyrin | QRM15926 | ANK35 |
| Pigeonpox virus | fep_165 | AID46669 | ANK16 | Albatrosspox virus | ankyrin | QRM16263 | ANK35 |
| Turkeypox virus | ankyrin | ALA62389 | ANK16 | Magpiepox virus | MPPV-305 | QZW33631 | ANK35 |
| Turkeypox virus | ankyrin | ALA62494 | ANK16 | Ectromelia virus | EVM022 | AAM92328 | ANK36 |
| Flamingopox virus | fgpv_170 | AUD40267 | ANK16 | Fowlpox virus | FPV227 | AAF44571 | ANK36 |
| Flamingopox virus | fgpv_172 | AUD40269 | ANK16 | Monkeypox virus | C1L | AAL40485 | ANK36 |
| Mudlarkpox virus | ankyrin | QRM15421 | ANK16 | Cowpox virus | CPXV041 | AAM13488 | ANK36 |
| Mudlarkpox virus | ankyrin | QRM15503 | ANK16 | Vaccinia virus | VACWR032 | AAO89311 | ANK36 |
| Penguinpox virus | ankyrin | QRM15776 | ANK16 | Canarypox virus | CNPV300 | AAR83646 | ANK36 |
| Penguinpox virus | ankyrin | QRM15858 | ANK16 | Taterapox virus | TATV_DAH68_034 | ABD97600 | ANK36 |
| Albatrosspox virus | ankyrin | QRM16108 | ANK16 | Taterapox virus | TATV_DAH68_035 | ABD97601 | ANK36 |
| Albatrosspox virus | ankyrin | QRM16194 | ANK16 | Taterapox virus | TATV_DAH68_206 | ABD97772 | ANK36 |
| Magpiepox virus | MPPV-151 | QZW33446 | ANK16 | Pigeonpox virus | fep_232 | AID46726 | ANK36 |
| Fowlpox virus | FPV246 | AAF44590 | ANK17 | Raccoonpox virus | RCNV-Herman-028 | AKJ93662 | ANK36 |
| Canarypox virus | CNPV011 | AAR83357 | ANK17 | Turkeypox virus | ankyrin | ALA62536 | ANK36 |
| Canarypox virus | CNPV021 | AAR83367 | ANK17 | Skunkpox virus | SKPV-WA-031 | AOP31510 | ANK36 |
| Canarypox virus | CNPV144 | AAR83490 | ANK17 | Volepox virus | VPXV-CA-031 | AOP31721 | ANK36 |
| Canarypox virus | CNPV150 | AAR83496 | ANK17 | BeAn 58058 virus | BAV00031 | APG58219 | ANK36 |
| Canarypox virus | CNPV291 | AAR83637 | ANK17 | Murmansk poxvirus | Murmansk-029 | AST09224 | ANK36 |
| Canarypox virus | CNPV310 | AAR83656 | ANK17 | NY_014 poxvirus | NY_014-025 | AST09426 | ANK36 |
| Canarypox virus | CNPV316 | AAR83662 | ANK17 | Flamingopox virus | fgpv_243 | AUD40334 | ANK36 |
| Canarypox virus | CNPV319 | AAR83665 | ANK17 | Akhmeta virus | AKMV-88-037 | AXN74822 | ANK36 |
| Pigeonpox virus | fep_252 | AID46738 | ANK17 | Alaskapox virus | ankyrin-like | QED21177 | ANK36 |
| Flamingopox virus | fgpv_272 | AUD40355 | ANK17 | Mudlarkpox virus | ankyrin | QRM15579 | ANK36 |
| Mudlarkpox virus | ankyrin | QRM15287 | ANK17 | Penguinpox virus | ankyrin | QRM15932 | ANK36 |
| Mudlarkpox virus | ankyrin | QRM15297 | ANK17 | Albatrosspox virus | ankyrin | QRM16269 | ANK36 |
| Mudlarkpox virus | ankyrin | QRM15422 | ANK17 | Magpiepox virus | MPPV-319 | QZW33651 | ANK36 |
| Mudlarkpox virus | ankyrin | QRM15570 | ANK17 | Abatino macacapox virus | KM543_gp034 | YP_010085702 | ANK36 |
| Mudlarkpox virus | ankyrin | QRM15589 | ANK17 | Variola virus | C1L | CAA48962 | ANK36 |
