# Supplementary material for: A poxvirus ankyrin protein LSDV012 inhibits IFIT1 in a host-species-specific manner by compromising its RNA binding ability
Source: PLoS Pathog. 2025 Mar 17;21(3):e1012994. doi: 10.1371/journal.ppat.1012994 (PMC11957390; doi:10.1371/journal.ppat.1012994)
Supplement: S2 Table — (DOCX) [file ppat.1012994.s005.docx]

S2 Table. VACV C9L homologs in different poxvirus

| Genus | Virus | Gene | ID | Group |
| --- | --- | --- | --- | --- |
| Orthopoxvirus | Abatino macacapox virus | KM543_gp020 | YP_010085688 | ANK6 |
|  | Akhmeta virus | AKMV-88-023 | AXN74808 | ANK6 |
|  | Alaskapox virus | ankyrin-like | QED21117 | ANK6 |
|  | Cowpox virus | CPXV027 | AAM13474 | ANK6 |
|  | Monkeypox virus | D9L | AAL40470 | ANK6 |
|  | Raccoonpox virus | RCNV-Herman-015 | AKJ93649 | ANK6 |
|  | Skunkpox virus | SKPV-WA-018 | AOP31497 | ANK6 |
|  | Taterapox virus | TATV_DAH68_020 | ABD97586 | ANK6 |
|  | Vaccinia virus | VACWR019 | AAO89298 | ANK6 |
|  | Volepox virus | VPXV-CA-018 | AOP31708 | ANK6 |
| Oryzopoxvirus | Cotia virus | COTV023 | YP_005296211 | ANK6 |
| Centapoxvirus | NY_014 poxvirus | NY_014-196 | AST09597 | ANK6 |
