# Supplementary material for: A poxvirus ankyrin protein LSDV012 inhibits IFIT1 in a host-species-specific manner by compromising its RNA binding ability
Source: PLoS Pathog. 2025 Mar 17;21(3):e1012994. doi: 10.1371/journal.ppat.1012994 (PMC11957390; doi:10.1371/journal.ppat.1012994)
Supplement: S3 Table — (DOCX) [file ppat.1012994.s006.docx]

S3 Table. LSDV012 interact with IFIT1 molecule to predict amino acid sites

| IFIT1 | | | | LSDV012 | | | |
| --- | --- | --- | --- | --- | --- | --- | --- |
| Site | Amino acid | Site | Amino acid | Site | Amino acid | Site | Amino acid |
| 147 | Trp | 340 | Leu | 2 | Glu | 81 | Asn |
| 150 | Leu | 344 | Ser | 3 | Lys | 82 | Phe |
| 151 | Lys | 347 | Glu | 4 | Glu | 83 | Lys |
| 153 | Gly | 348 | Met | 5 | Lys | 85 | Arg |
| 154 | Gly | 351 | Glu | 6 | Leu | 86 | Asp |
| 155 | Lys | 370 | Ala | 7 | Cys | 87 | Asn |
| 157 | Tyr | 372 | Asp | 8 | Ser | 88 | Asn |
| 183 | Ile | 373 | Lys | 9 | Asp | 94 | His |
| 186 | Tyr | 374 | Glu | 10 | Tyr | 97 | Ser |
| 187 | Arg | 375 | Val | 11 | Asp | 98 | Phe |
| 189 | Glu | 378 | His | 12 | Asn | 100 | Lys |
| 190 | Gly | 381 | Leu | 13 | Asp | 122 | Glu |
| 191 | Phe | 382 | His | 14 | Phe | 123 | Asn |
| 192 | Asn | 385 | Arg | 15 | Thr | 125 | Lys |
| 193 | Lys | 389 | Phe | 16 | Asp | 130 | Met |
| 217 | Tyr | 390 | His | 17 | Tyr | 133 | Cys |
| 227 | Gln | 409 | Ser | 19 | Phe | 135 | Phe |
| 228 | Asp | 411 | Ser | 20 | Tyr | 158 | Asp |
| 230 | Glu | 412 | Thr | 21 | Arg | 164 | Tyr |
| 232 | Glu | 413 | Asp | 22 | Tyr | 168 | Leu |
| 251 | Tyr | 415 | Asp | 23 | Cys | 169 | Phe |
| 254 | Arg | 416 | Lys | 27 | Phe | 190 | Asn |
| 255 | Tyr | 419 | Asn | 28 | Tyr | 192 | Ser |
| 258 | Lys | 420 | Ser | 31 | Glu | 194 | Tyr |
| 261 | Arg | 422 | Thr | 32 | Gln | 199 | Leu |
| 262 | Arg | 423 | Lys | 36 | Asn | 202 | Phe |
| 263 | Lys | 426 | Ser | 39 | Lys | 203 | Arg |
| 264 | Gly | 427 | Lys | 40 | Lys |  |  |
| 285 | Phe | 429 | Leu | 41 | Trp |  |  |
| 288 | His | 430 | Arg | 43 | Lys |  |  |
| 289 | Gln | 433 | Pro | 44 | Phe |  |  |
| 292 | Leu | 442 | Leu | 46 | Asn |  |  |
| 295 | Lys | 446 | His | 47 | Asp |  |  |
| 296 | Ser | 449 | Lys | 48 | Cys |  |  |
| 298 | Ile | 451 | Glu | 49 | Asn |  |  |
| 299 | Ser | 458 | Tyr | 50 | Asp |  |  |
| 300 | Gln | 461 | Gln | 51 | Leu |  |  |
| 302 | Lys | 465 | Leu | 52 | Tyr |  |  |
| 303 | Phe | 473 | Ala | 53 | Glu |  |  |
| 306 | Asn | 474 | Val | 54 | Thr |  |  |
| 307 | Cys | 475 | Ile | 58 | Ser |  |  |
| 308 | Gln | 476 | Pro | 61 | Glu |  |  |
| 335 | Lys | 478 | His | 63 | Asp |  |  |
| 338 | Phe |  |  | 79 | Asp |  |  |
