# Supplementary material for: A poxvirus ankyrin protein LSDV012 inhibits IFIT1 in a host-species-specific manner by compromising its RNA binding ability
Source: PLoS Pathog. 2025 Mar 17;21(3):e1012994. doi: 10.1371/journal.ppat.1012994 (PMC11957390; doi:10.1371/journal.ppat.1012994)
Supplement: S1 Data — (PDF) [file ppat.1012994.s009.pdf]

Original Images for Blots and IF

A poxvirus ankyrin protein LSDV012 inhibits IFIT1 in a host-species-specific manner by compromising its RNA binding ability

Shijie Xie, Yongxiang Fang, Zhiyi Liao,Xiaojing Hao, Lianxin Cui, Kang Niu, Shuning Ren, Junda Zhu, Wenxue Wu, Zhizhong Jing, Chen Peng

Figure 1K

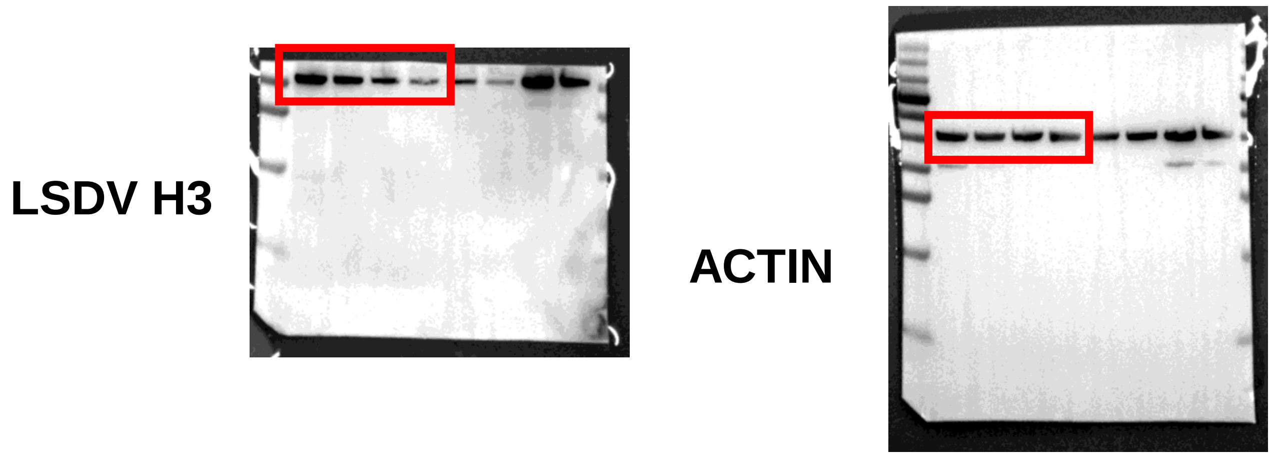

Figure 2D

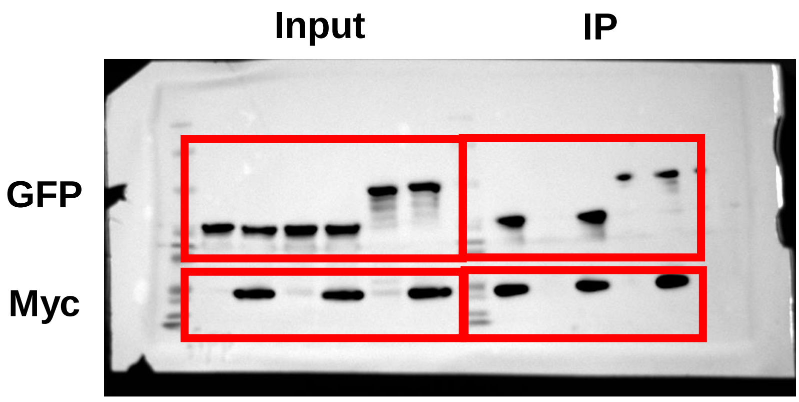

Figure 2F

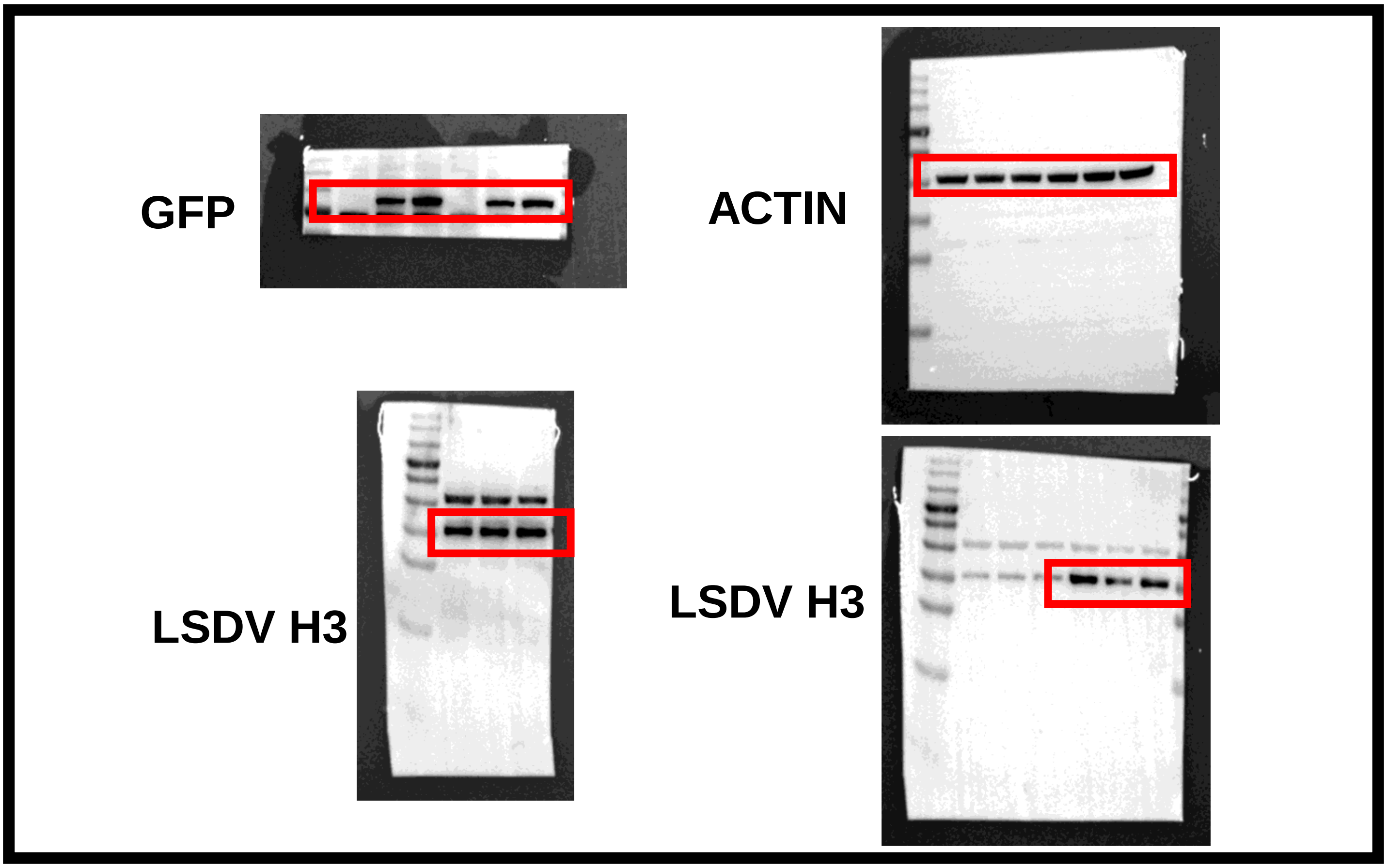

Figure 2I

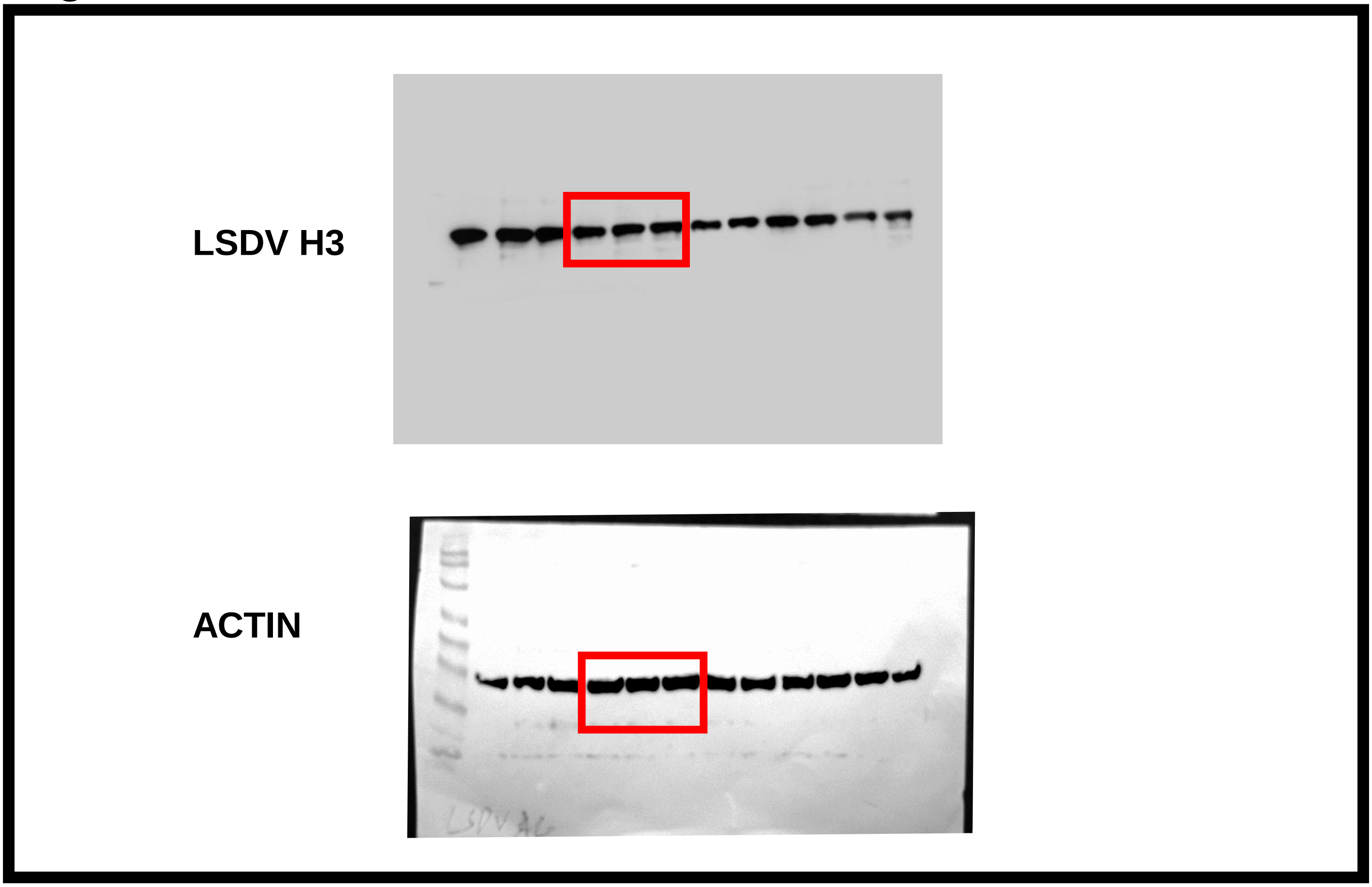

Figure 2J

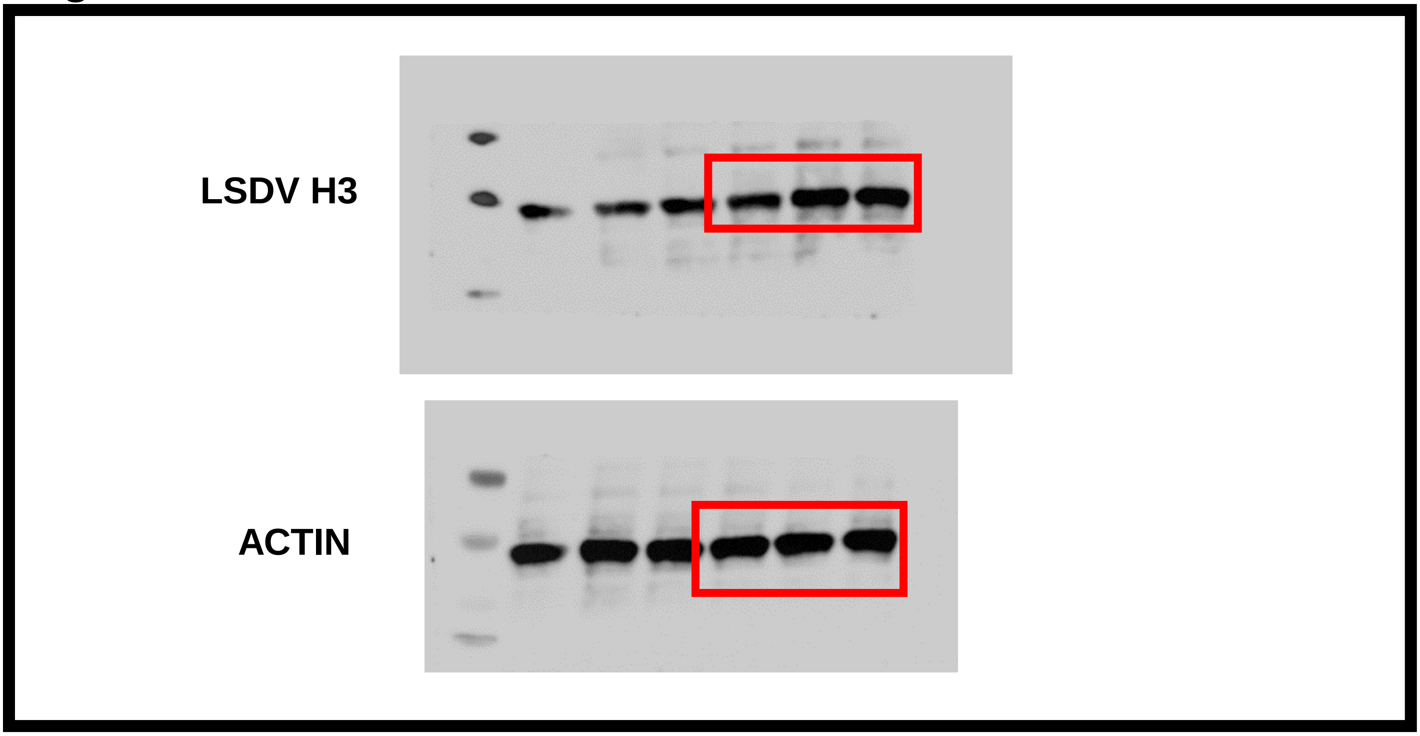

Figure 3A

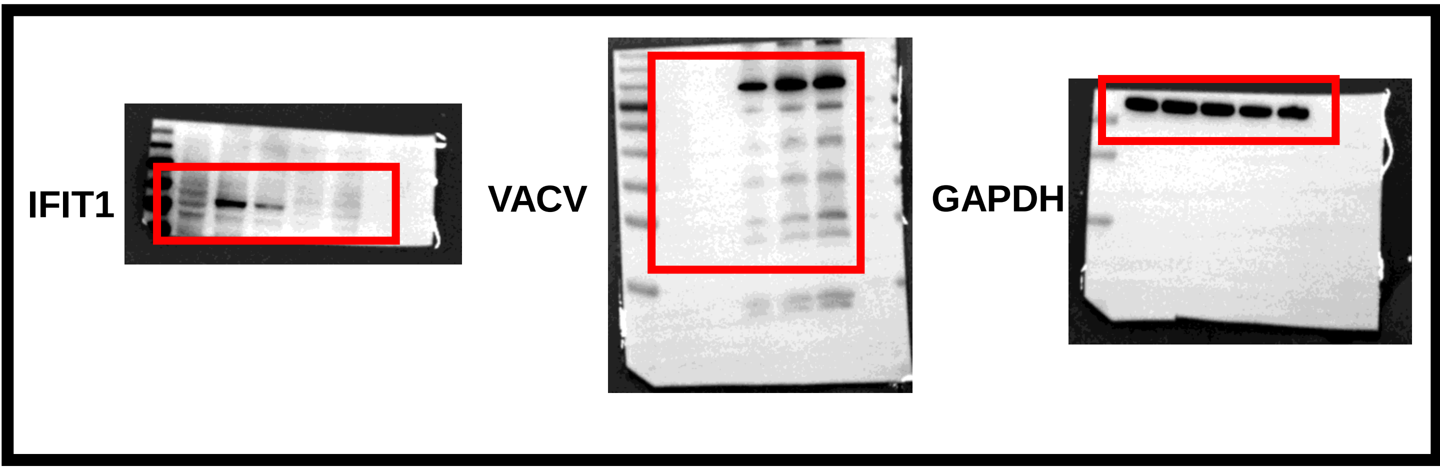

Figure 3B

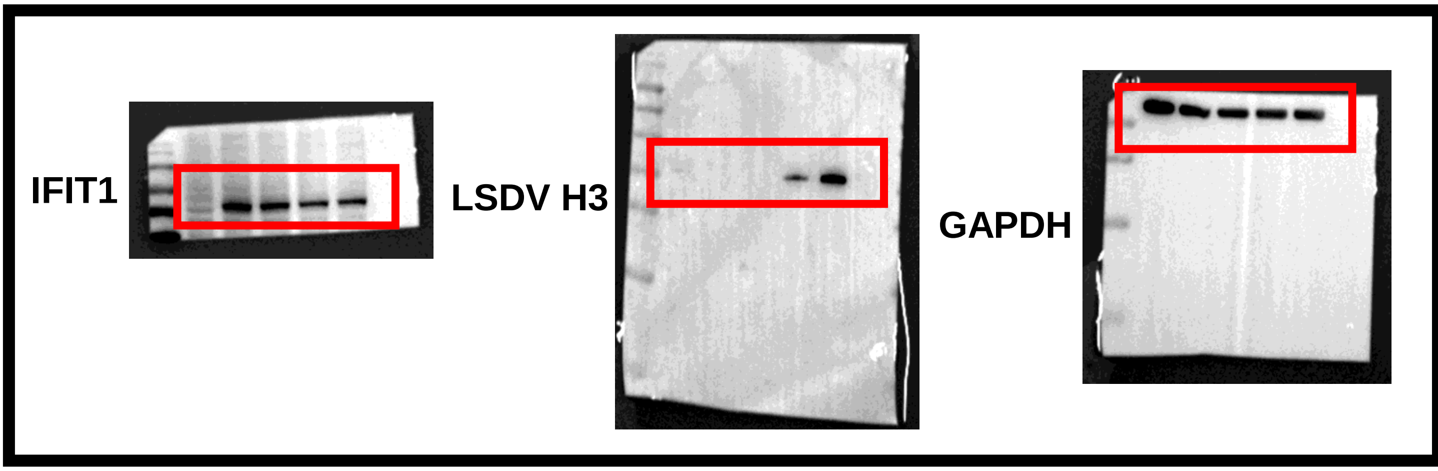

Figure 3C

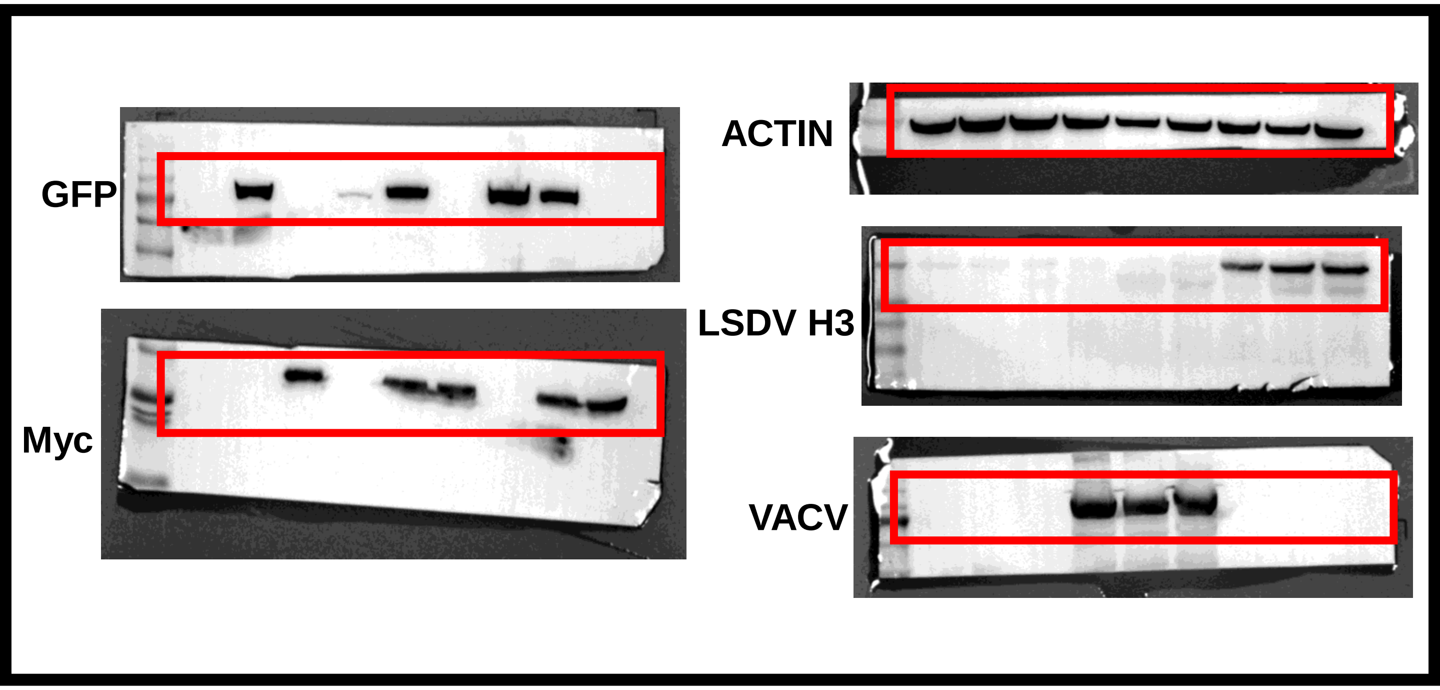

Figure 3D and 3E

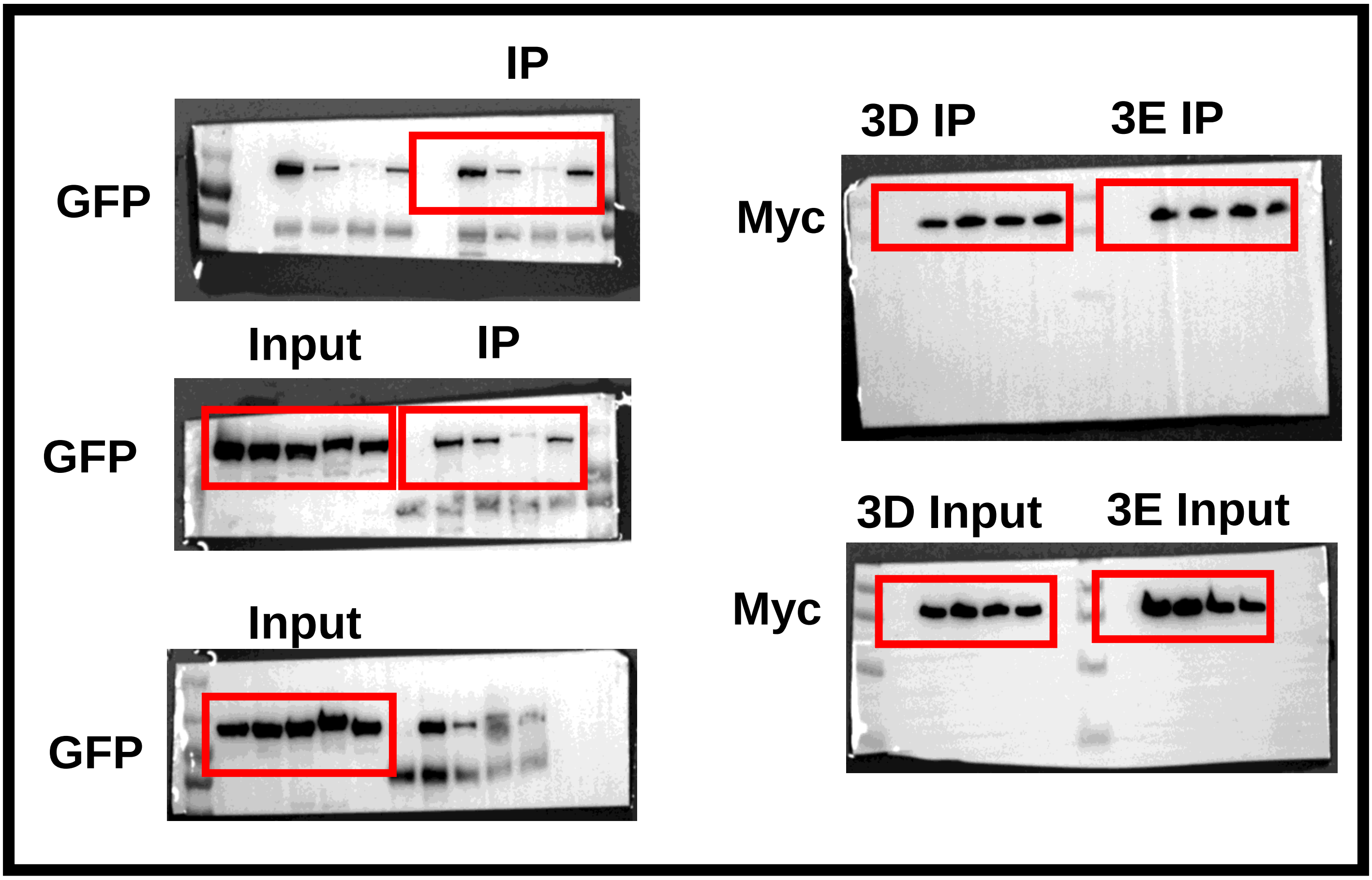

Figure 3G

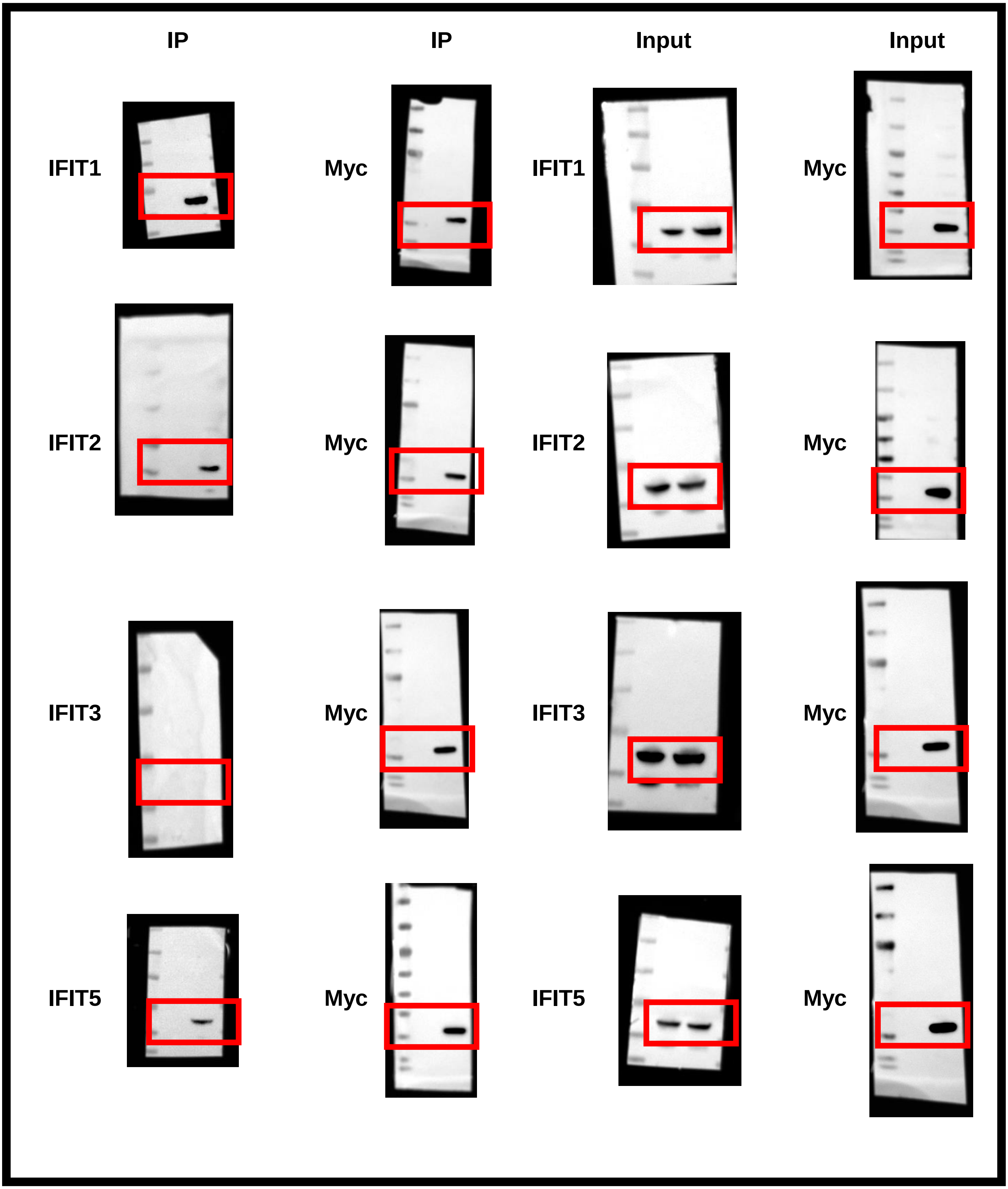
IP
IP
Input
Input
IFIT1
Myc
IFIT1
Myc
IFIT2
Myc
IFIT2
Myc
IFIT3
Myc
IFIT3
Myc
IFIT5
Myc
IFIT5
Myc

Figure 3H and 3J

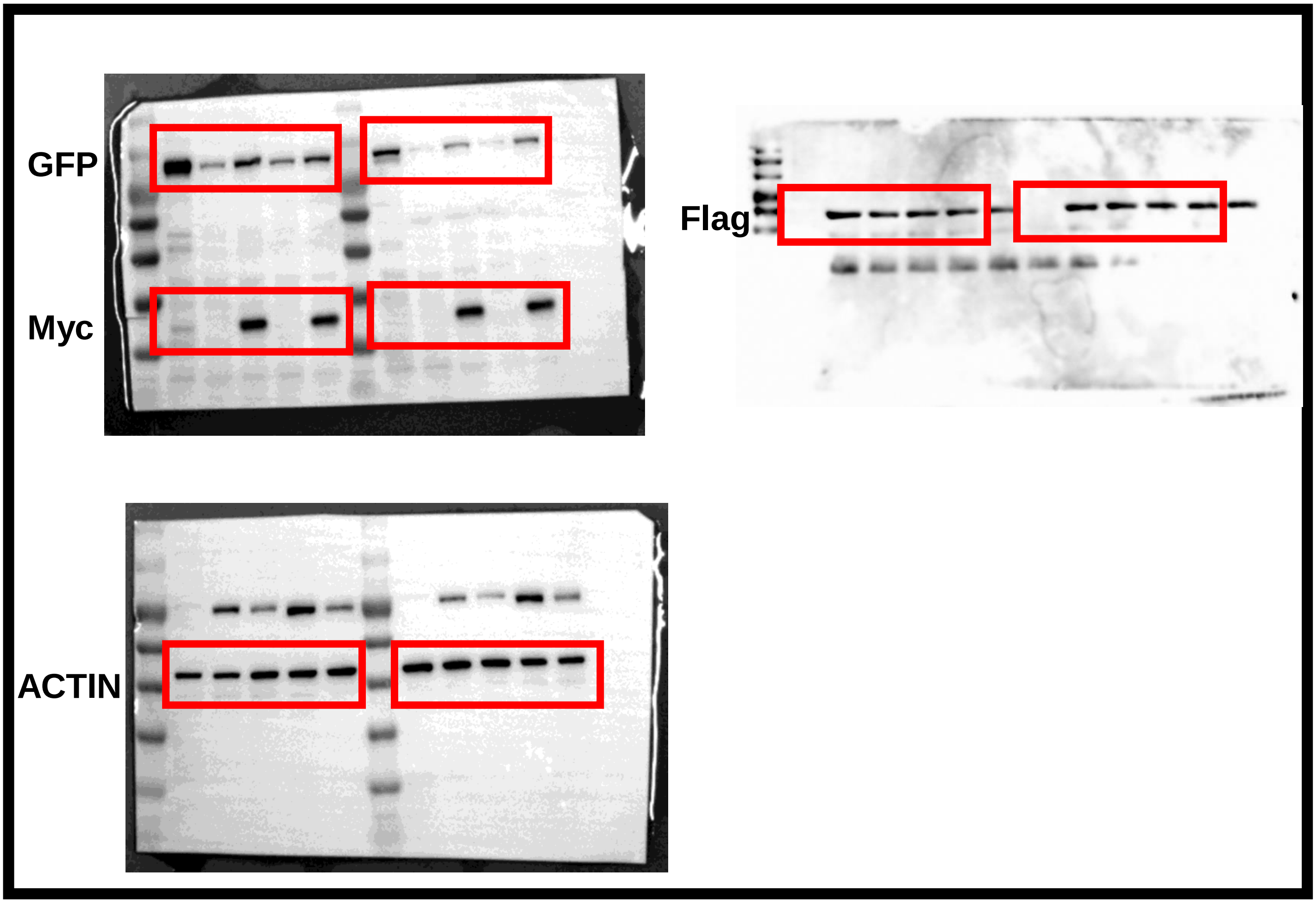

Figure 3L and 3N

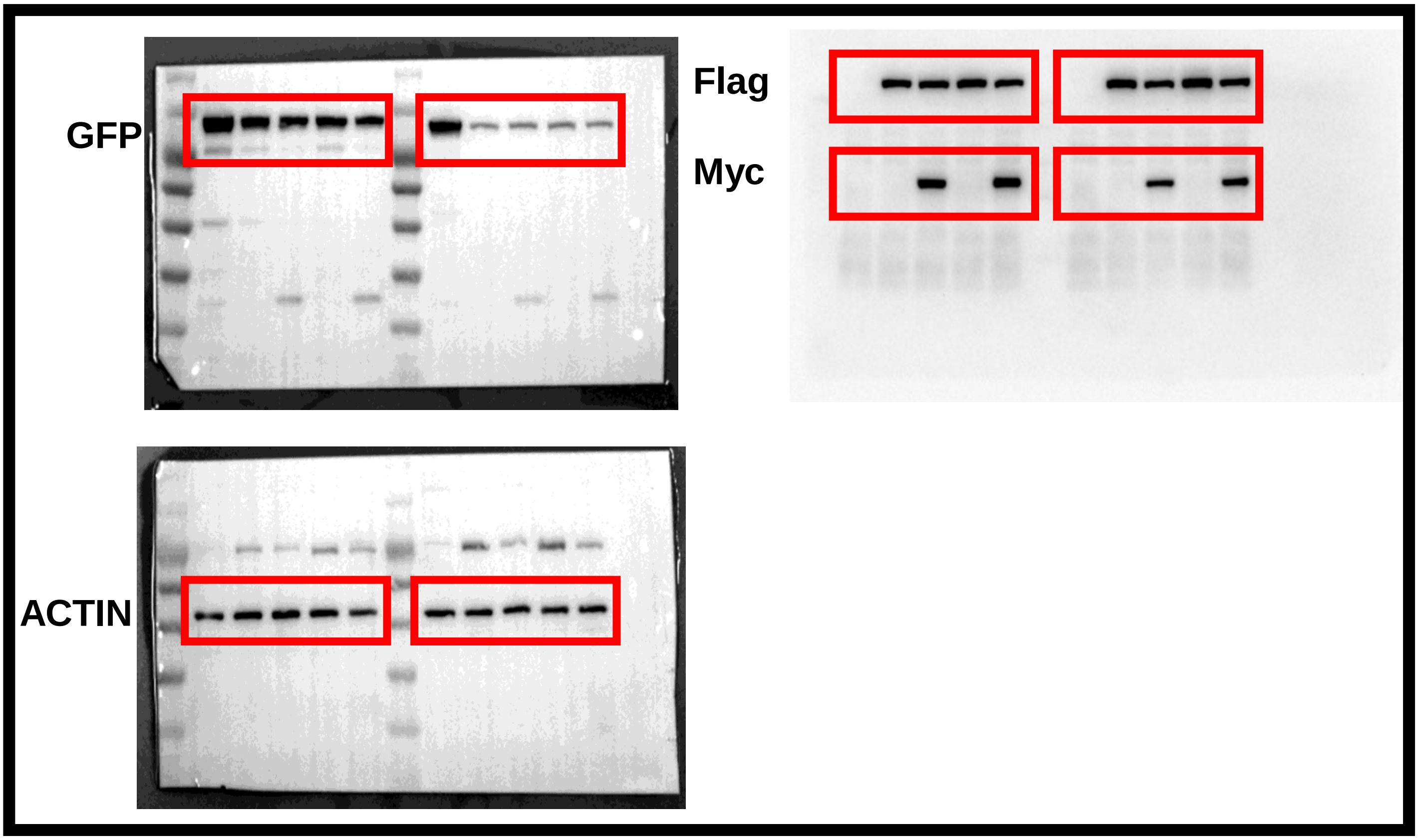

Figure 5A

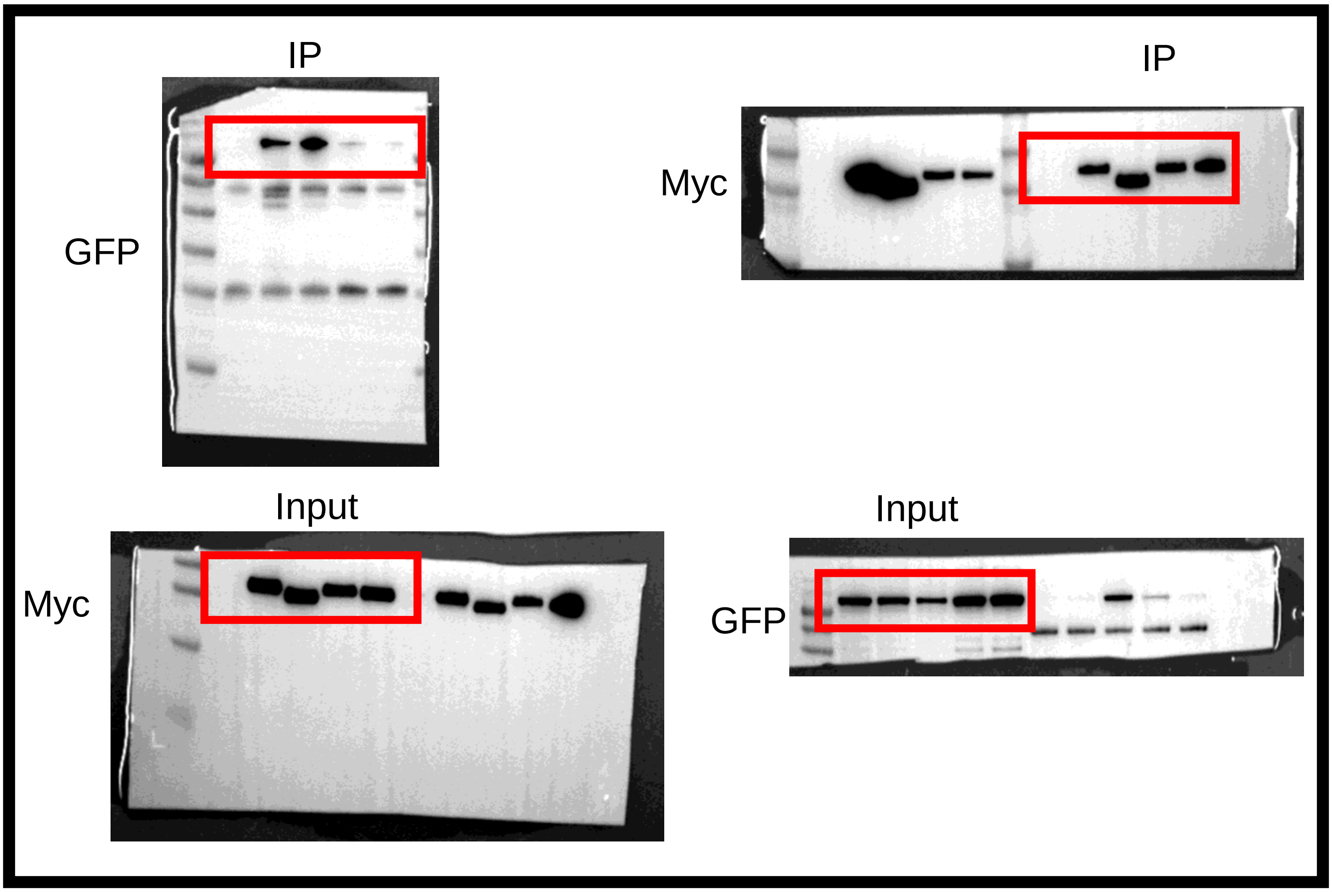

Figure 5B

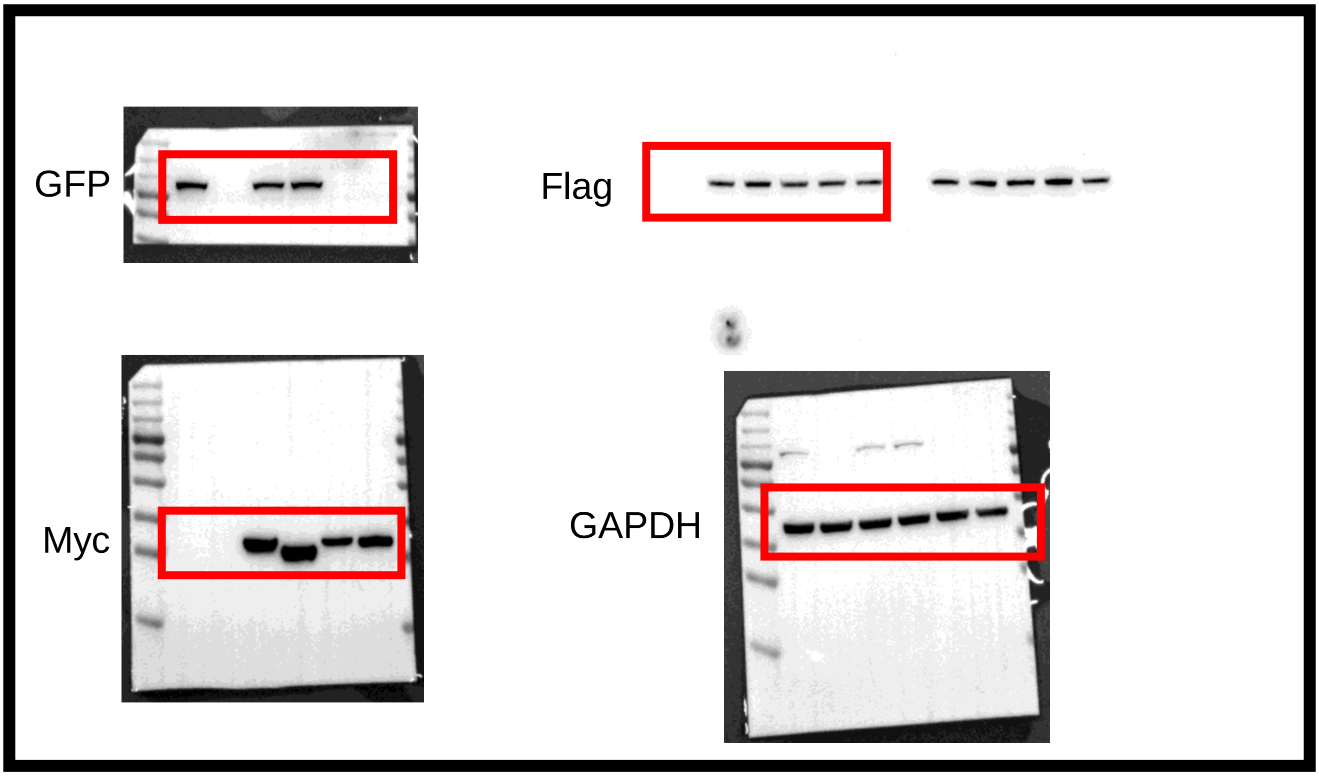

Figure 5D

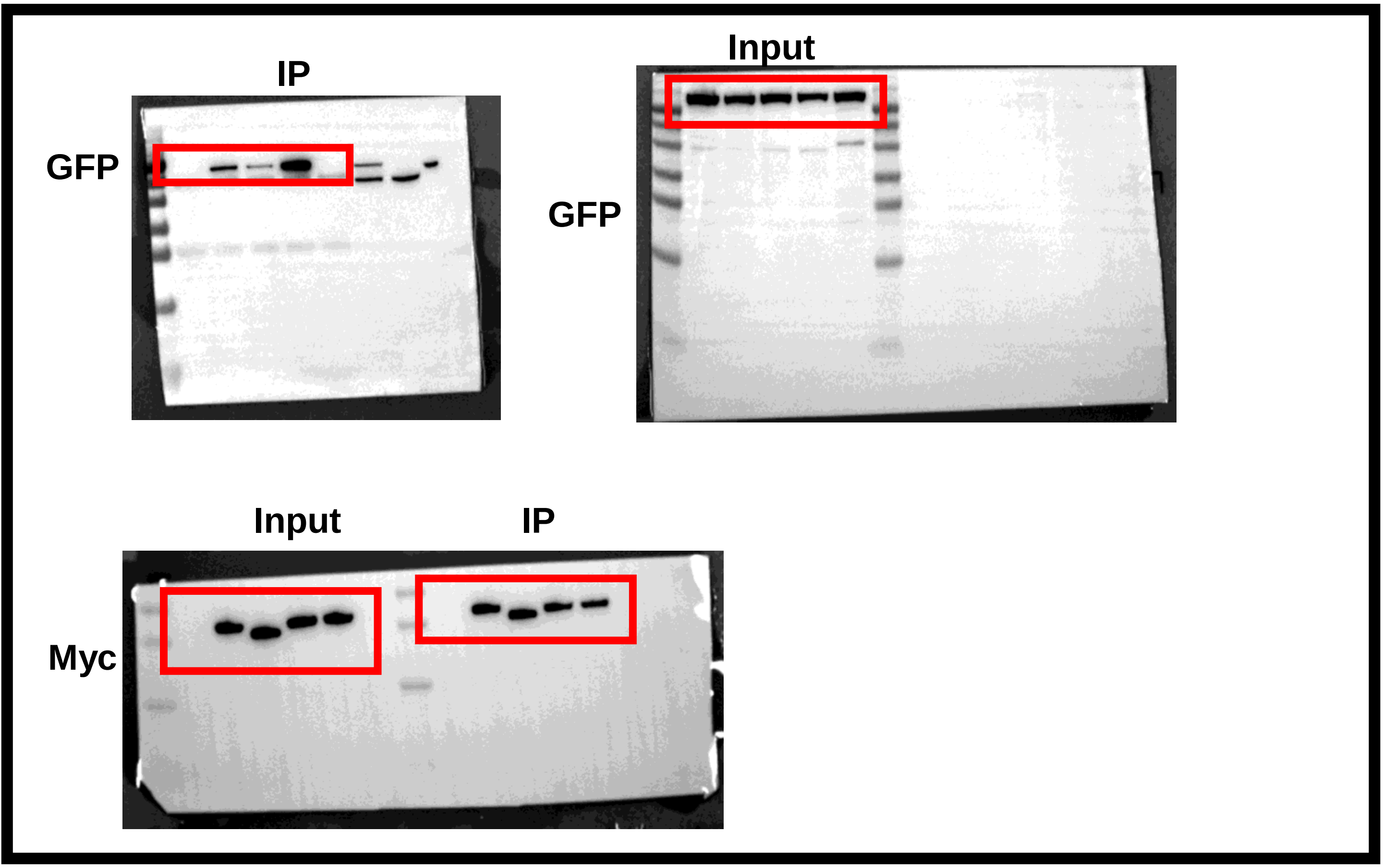

Figure 5E

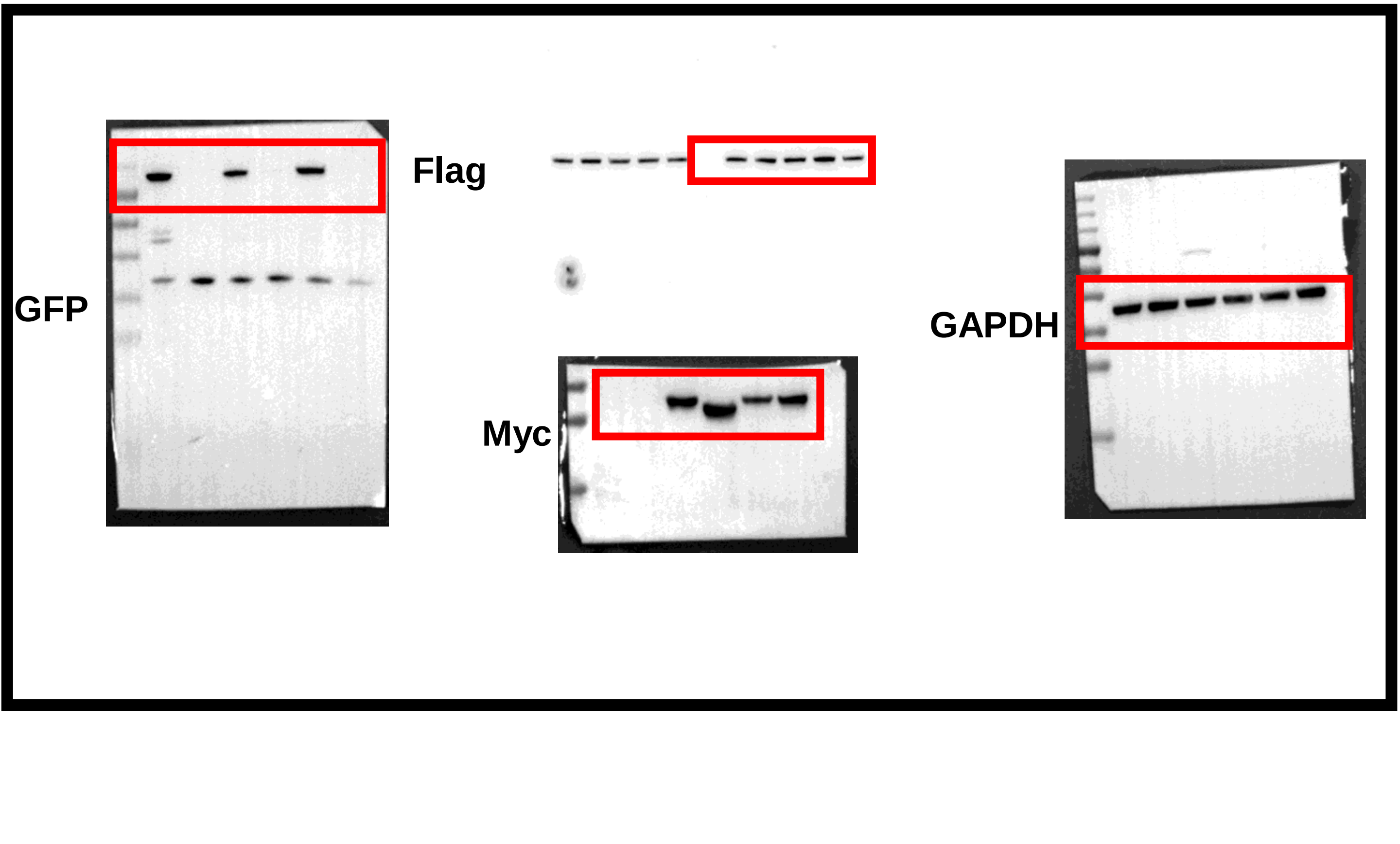

Figure 5G

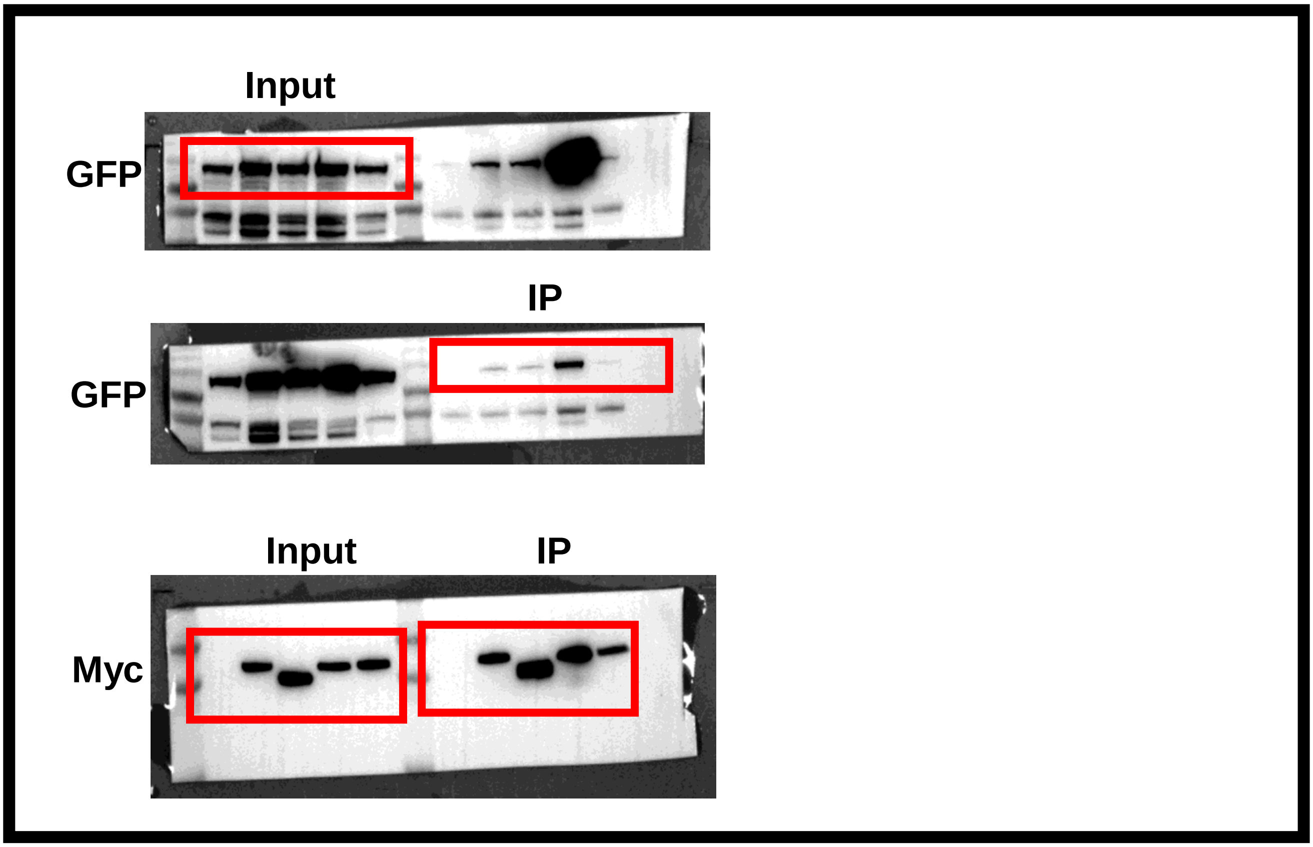

Figure 5H

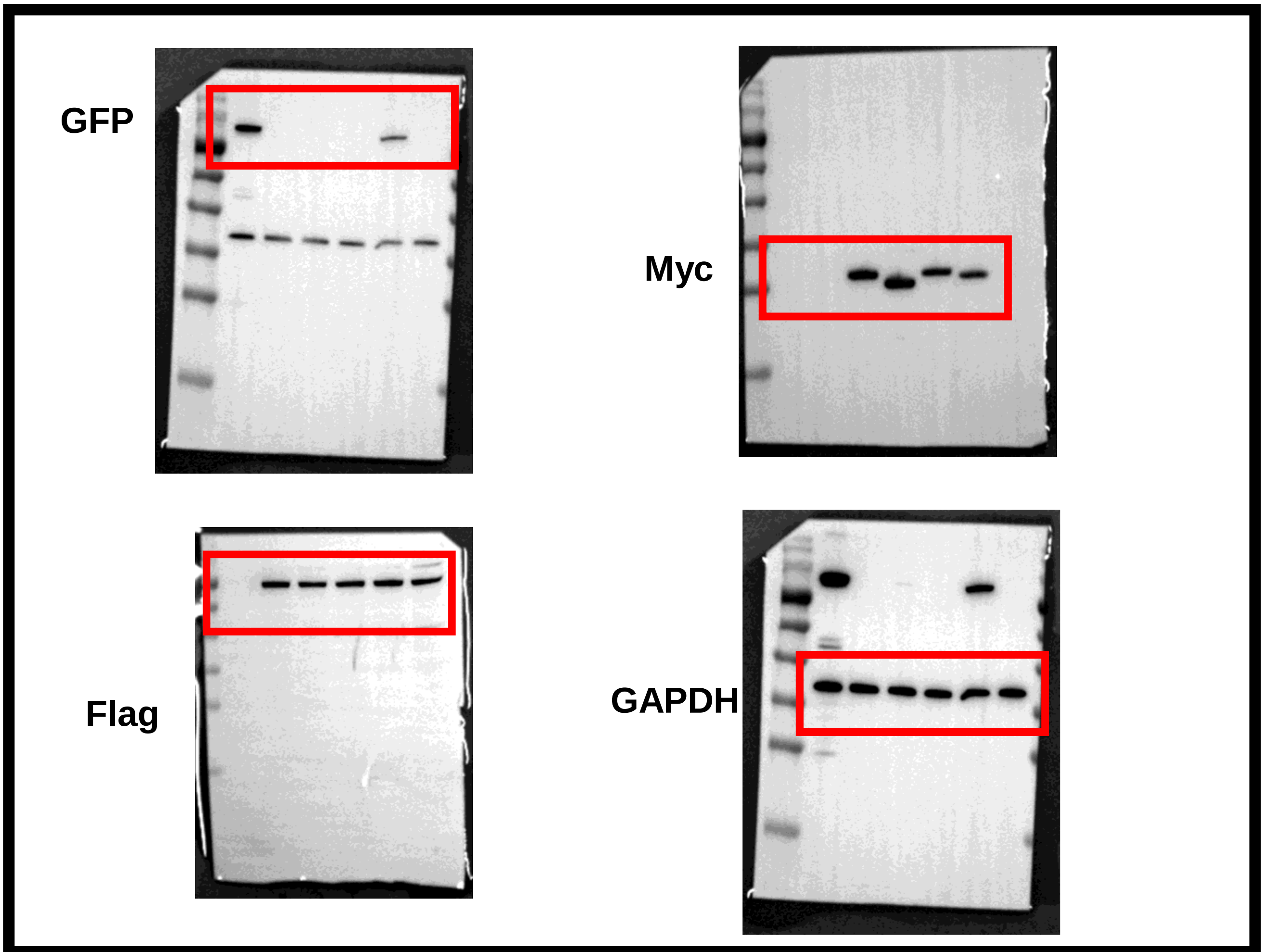

Figure 6F

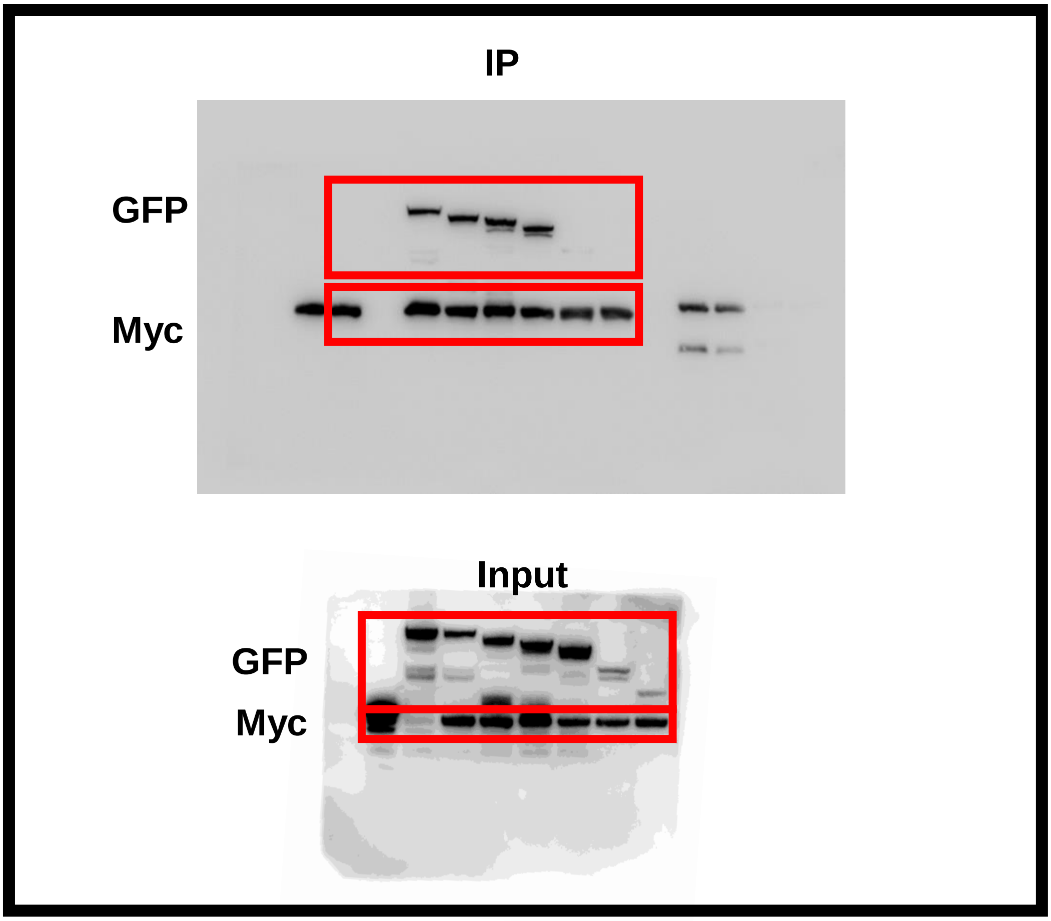

Figure 6H

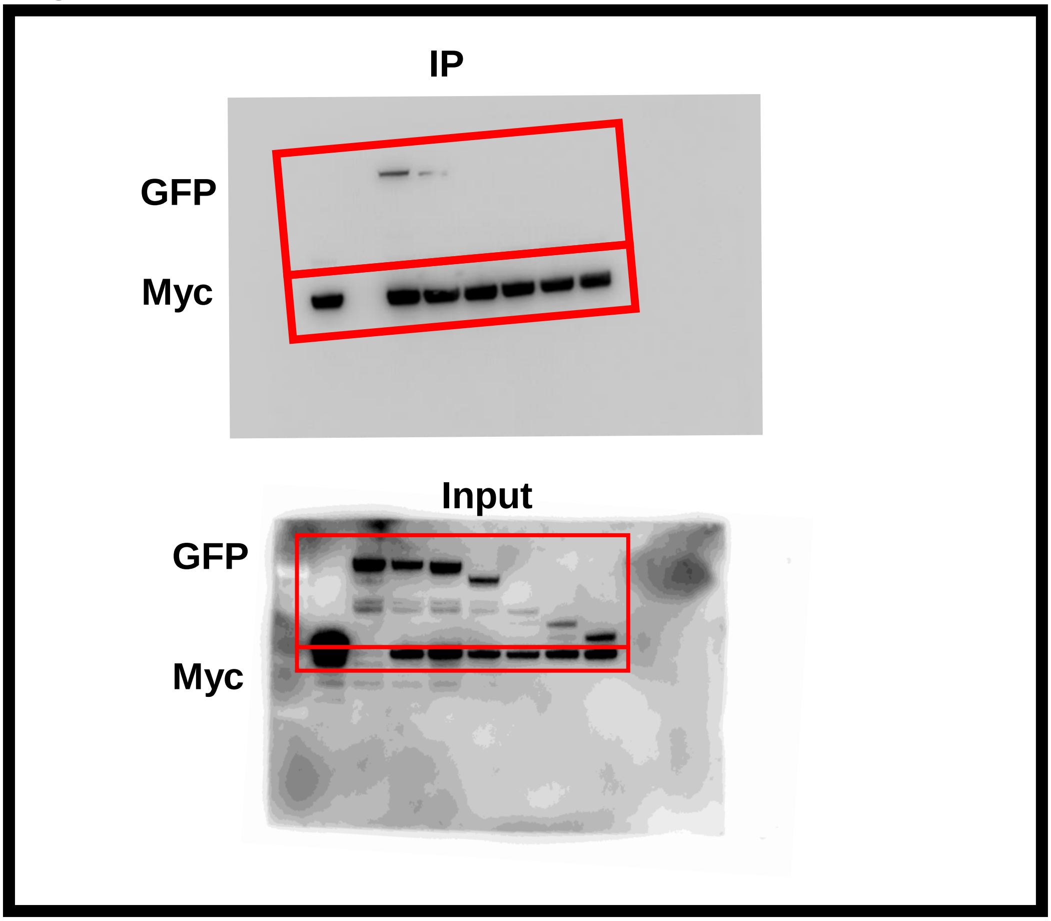

Figure 6J

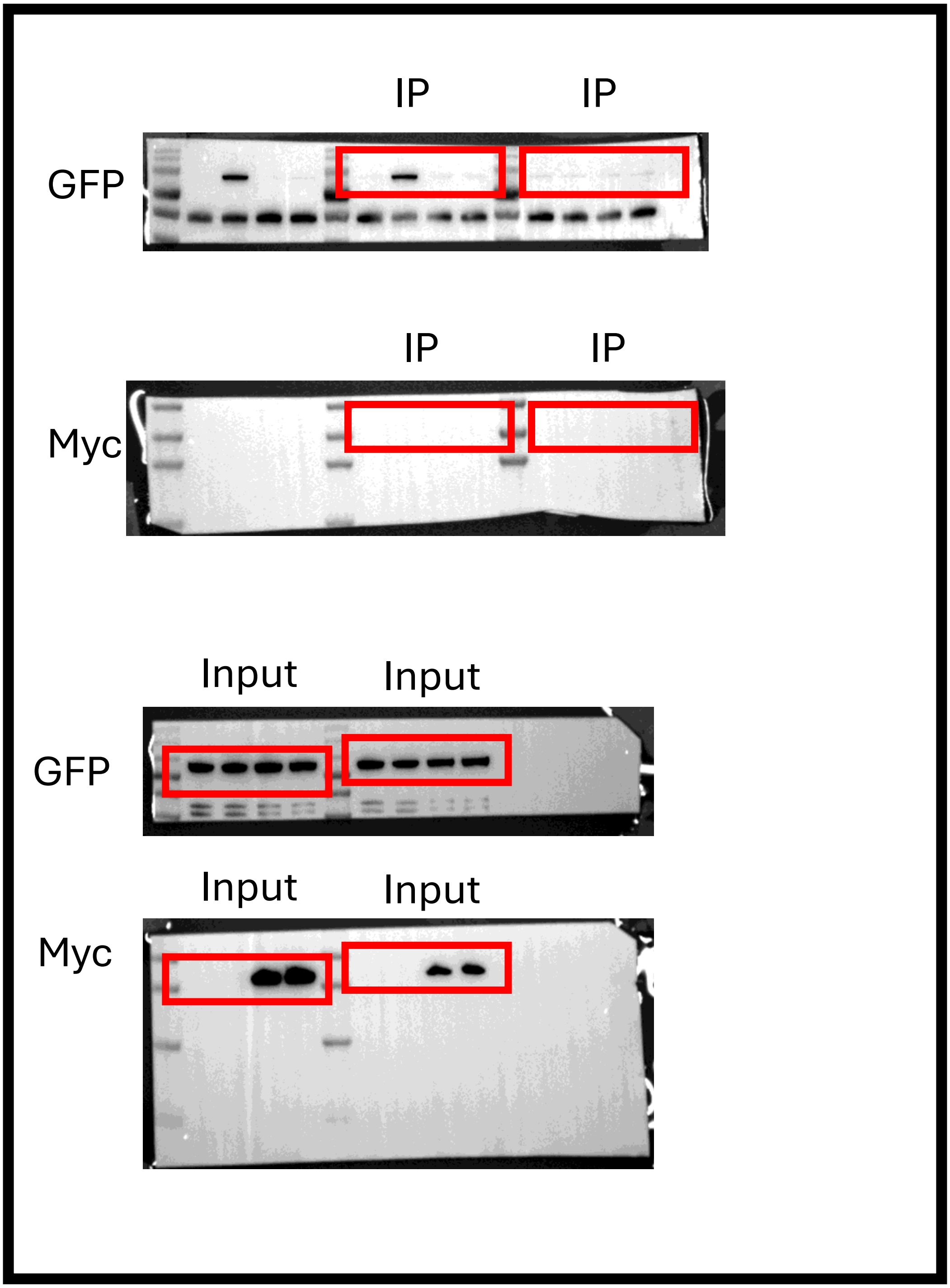

Figure 6K

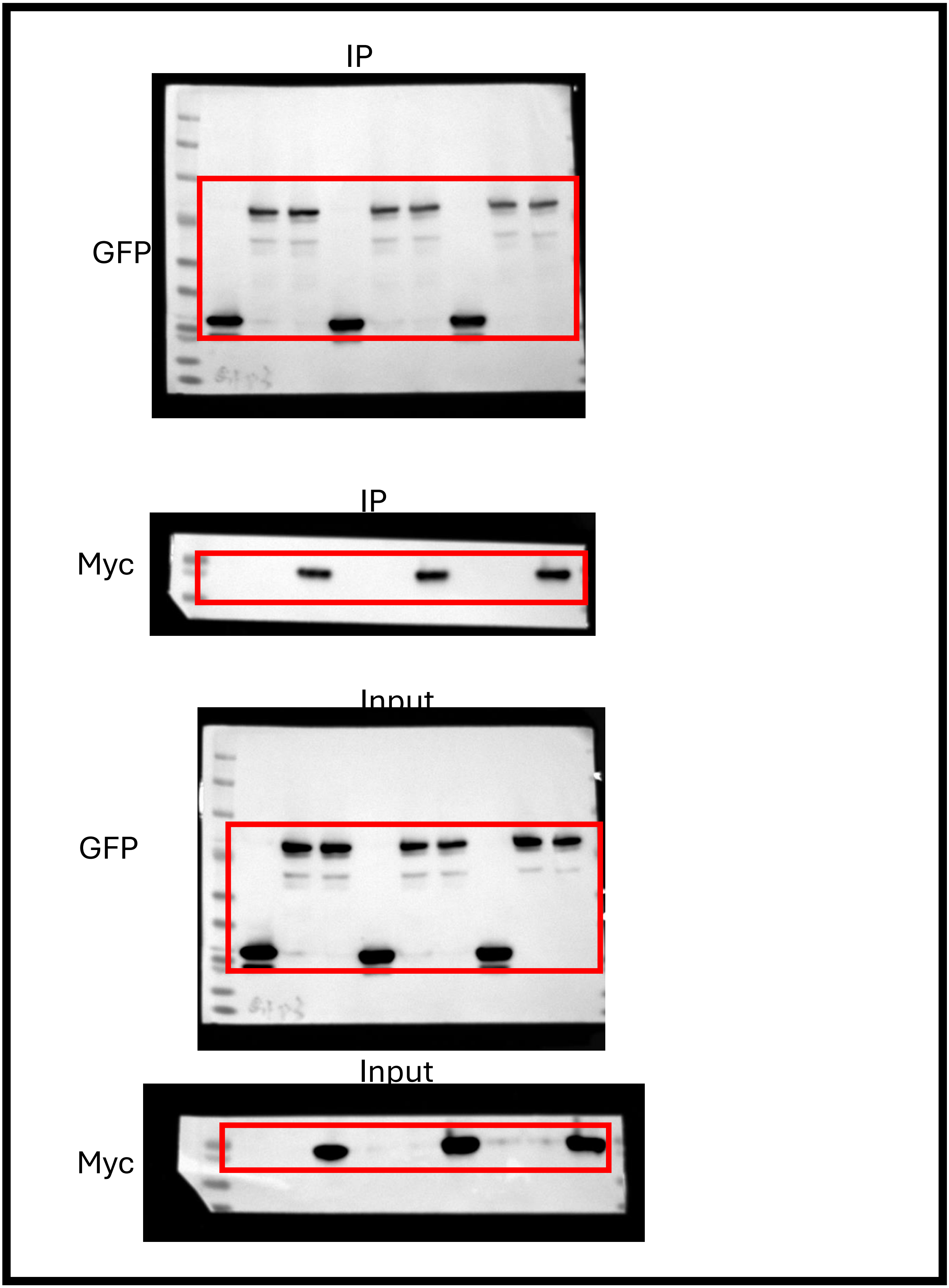

Figure S1E

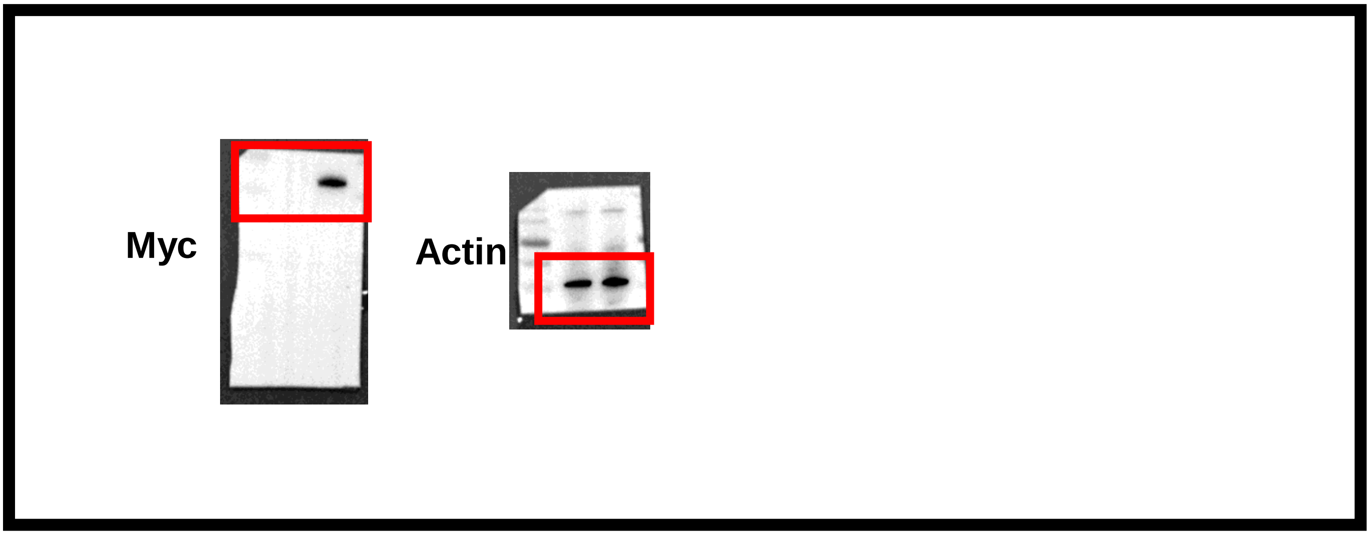

Figure S1I

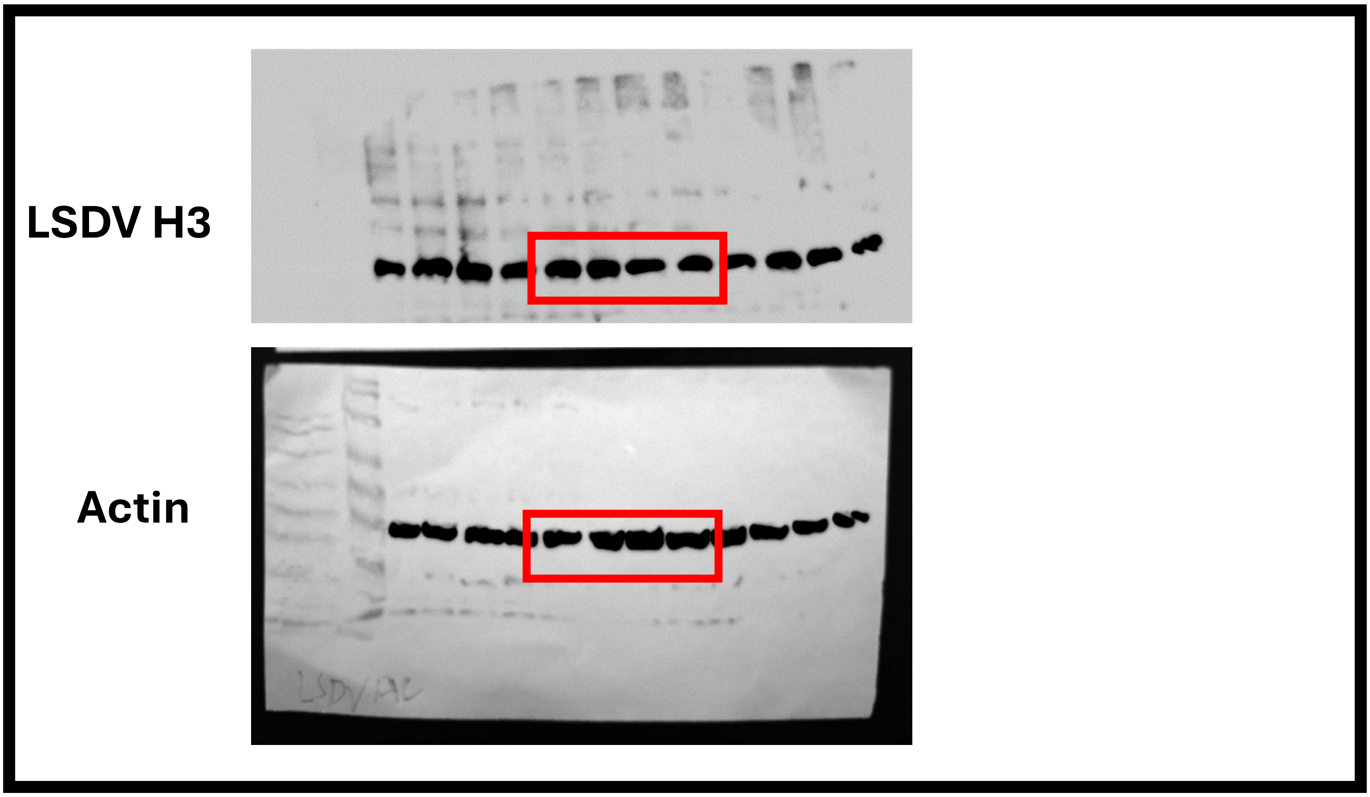

Figure S2B

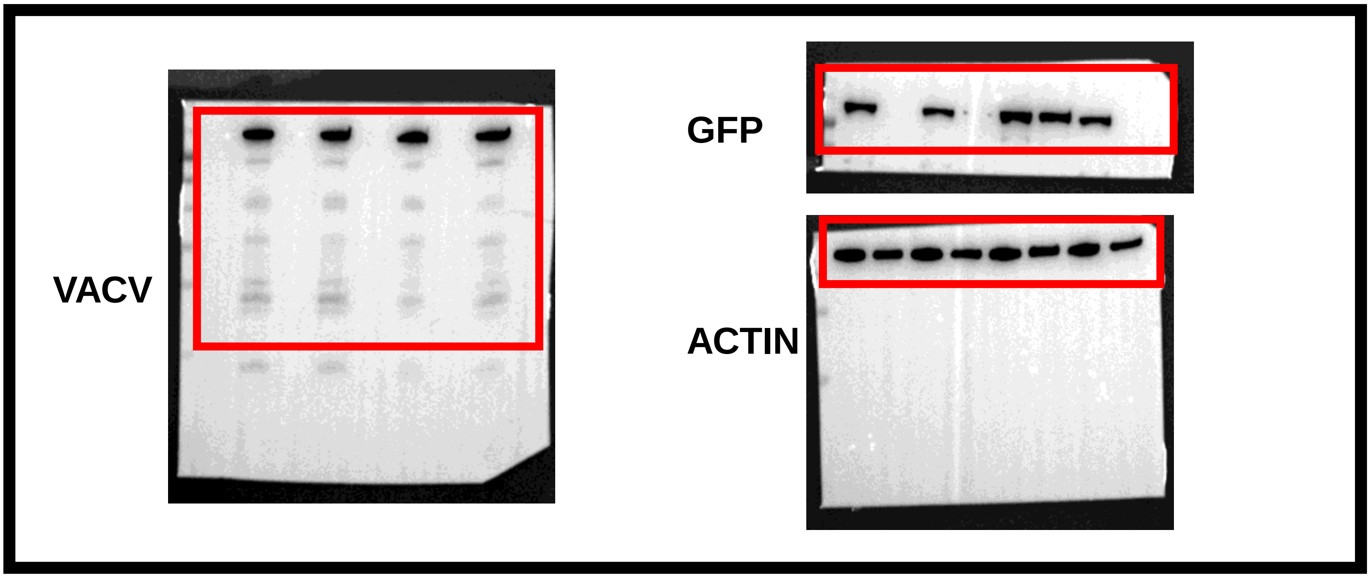

Figure S2C

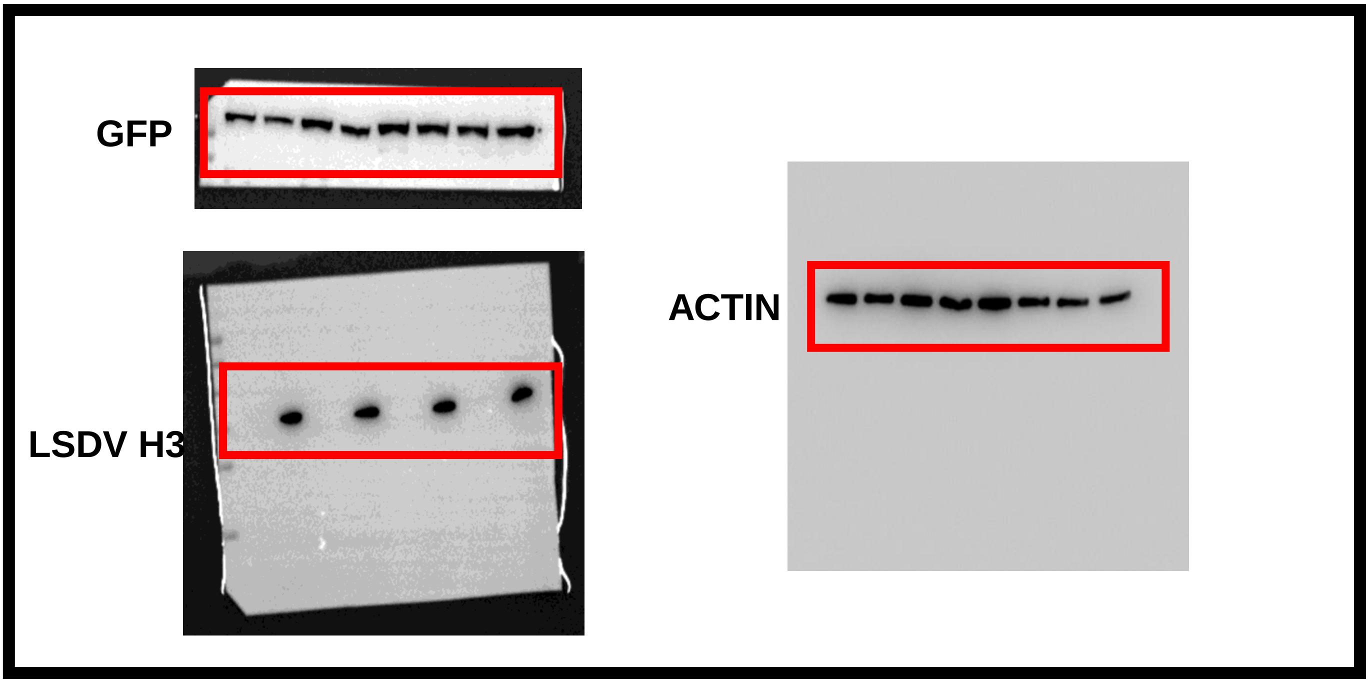

Figure S2D

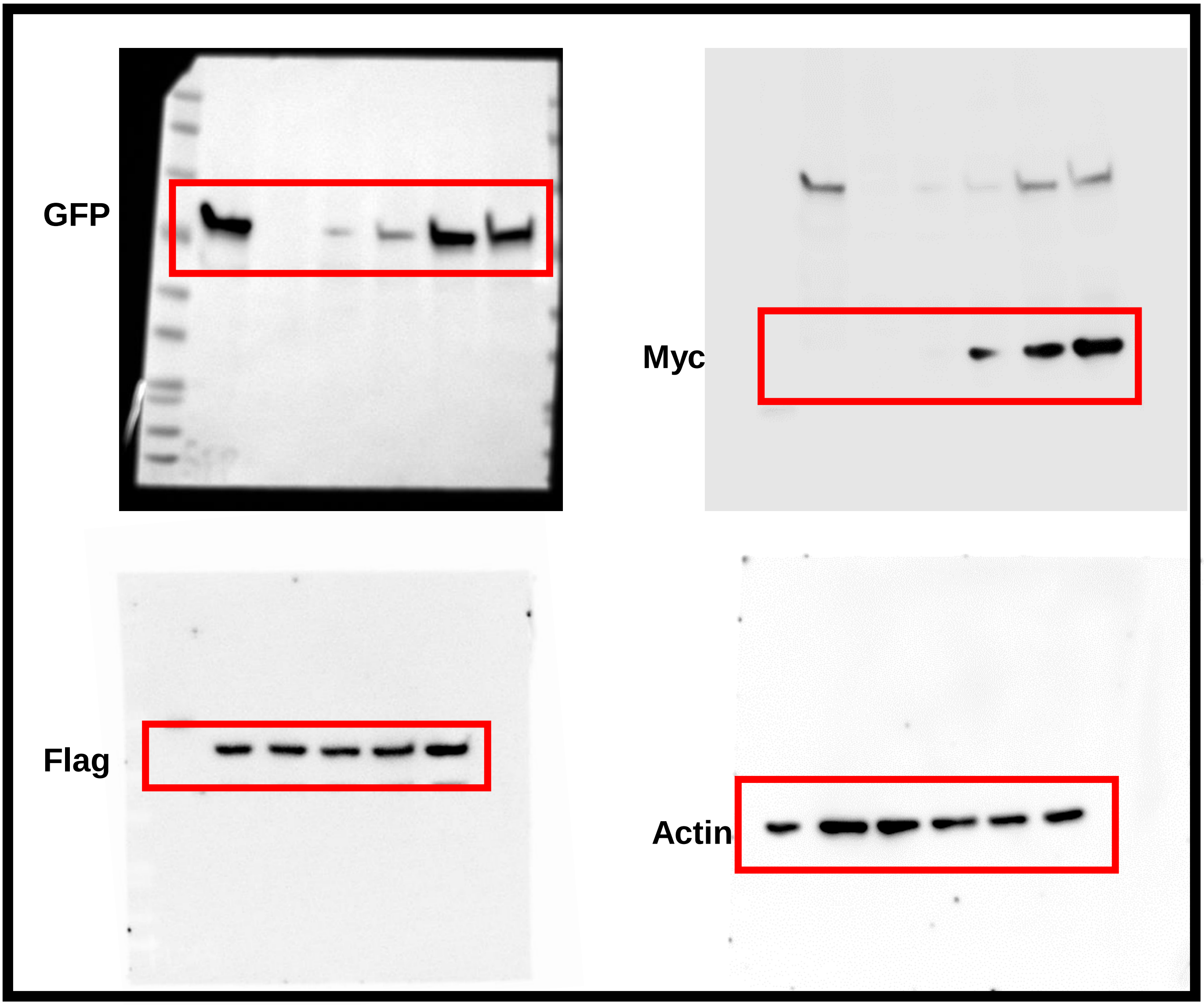

Figure S2E

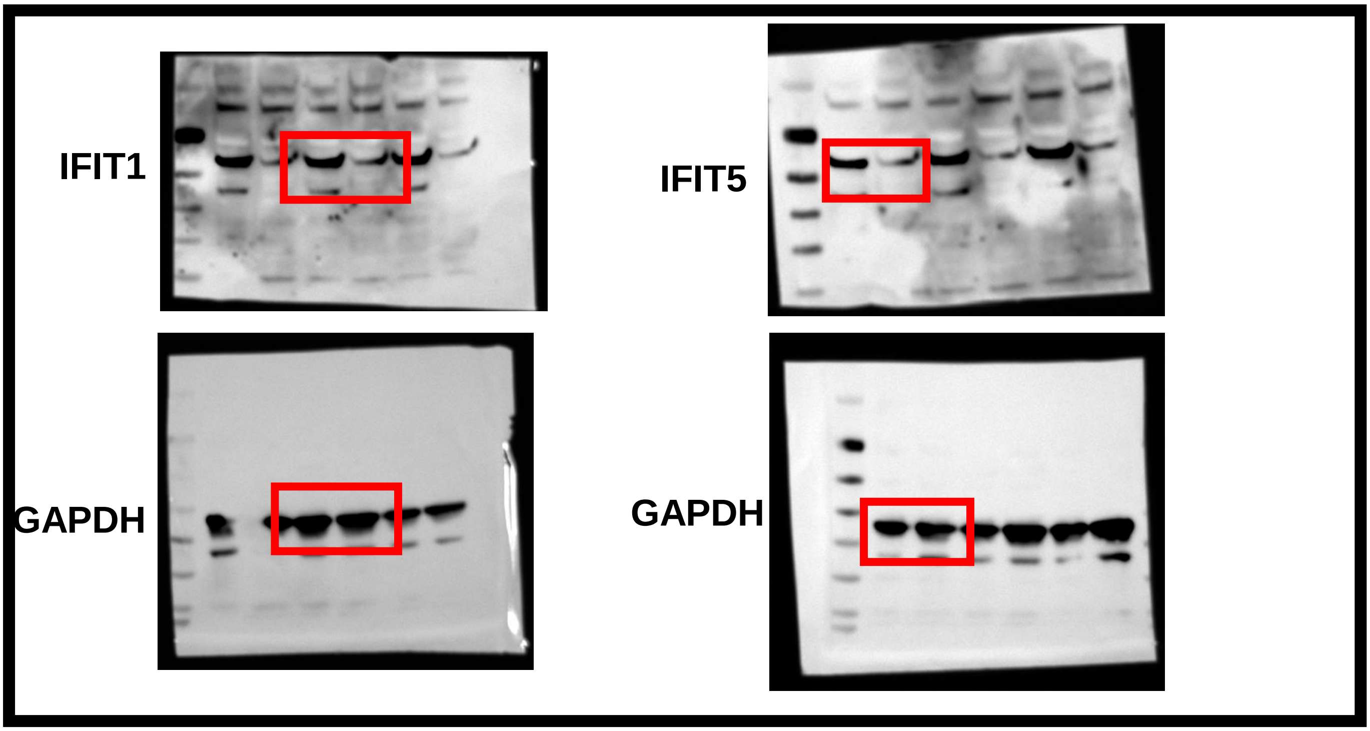

Figure S2G

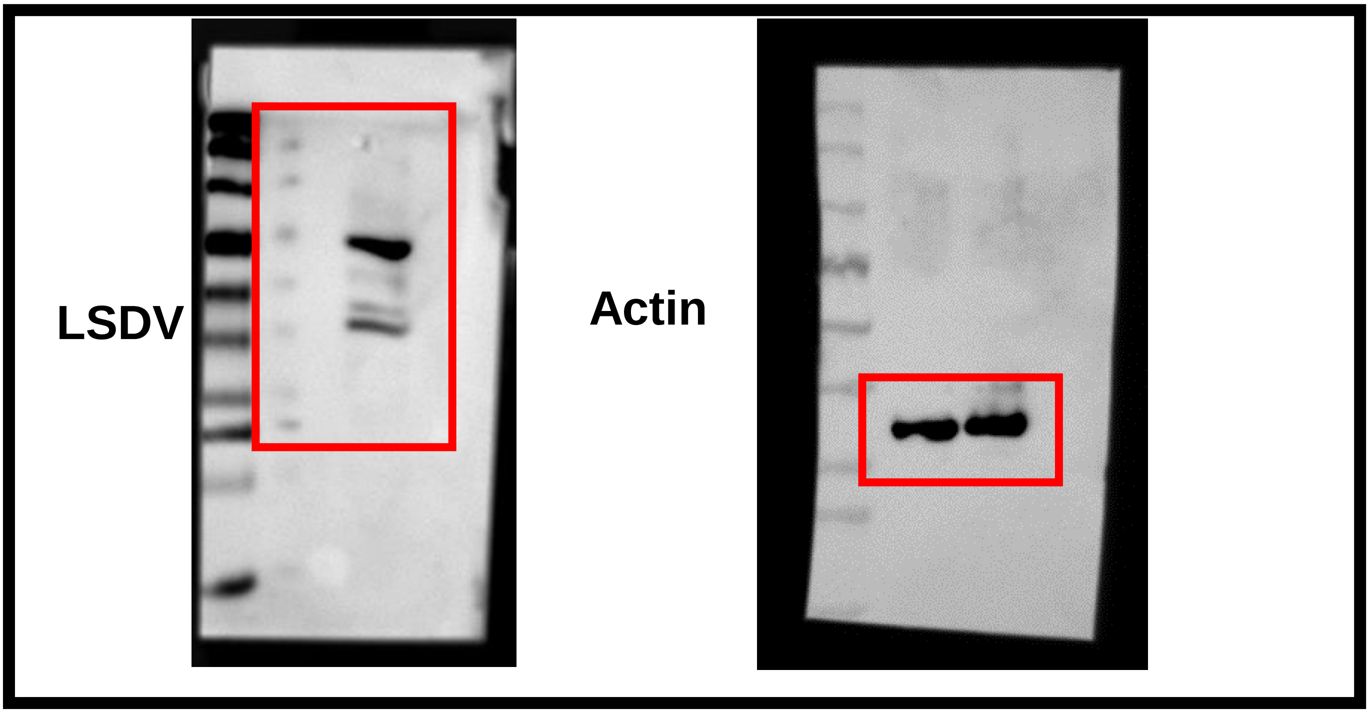

Figure S2H

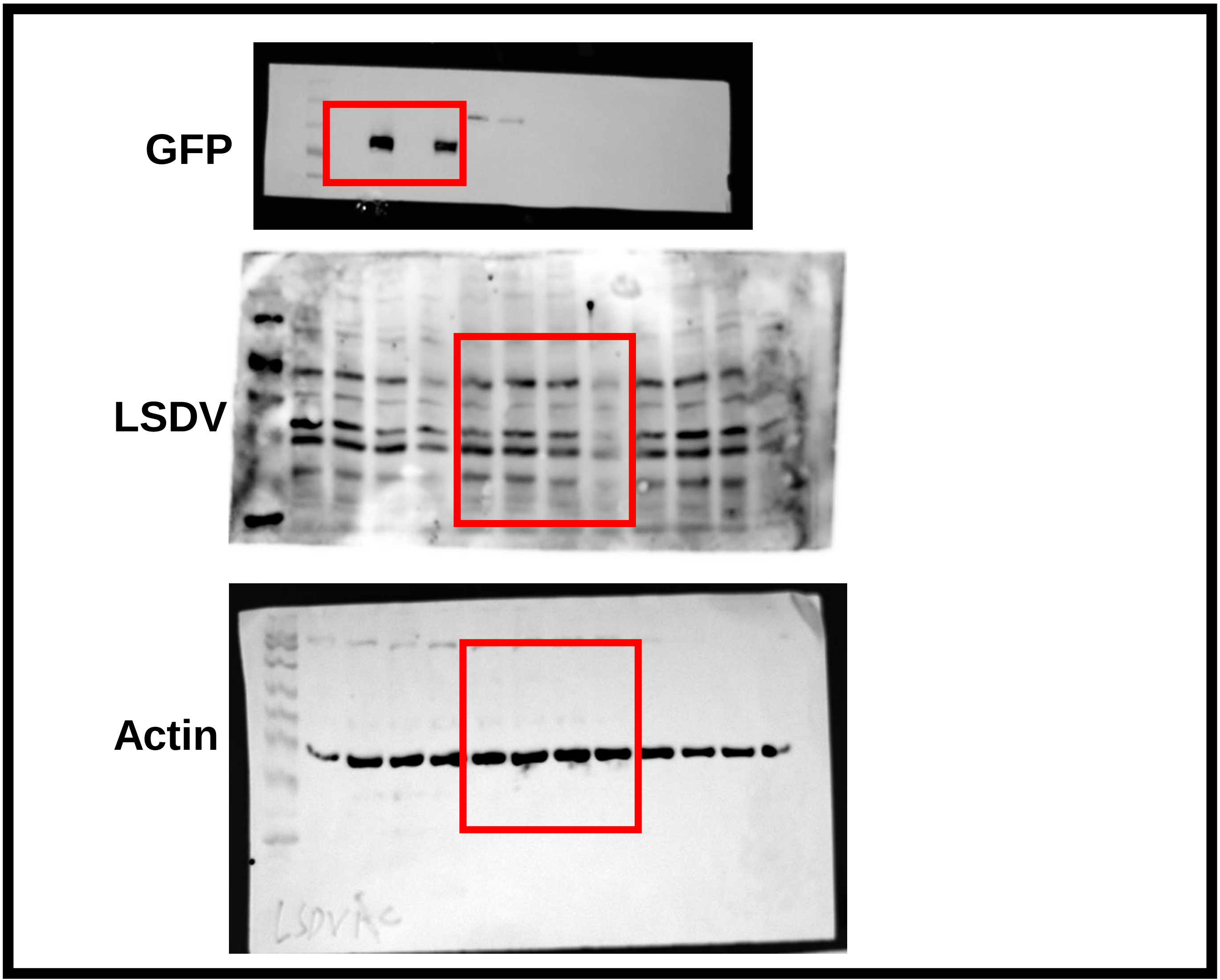

Figure 1G

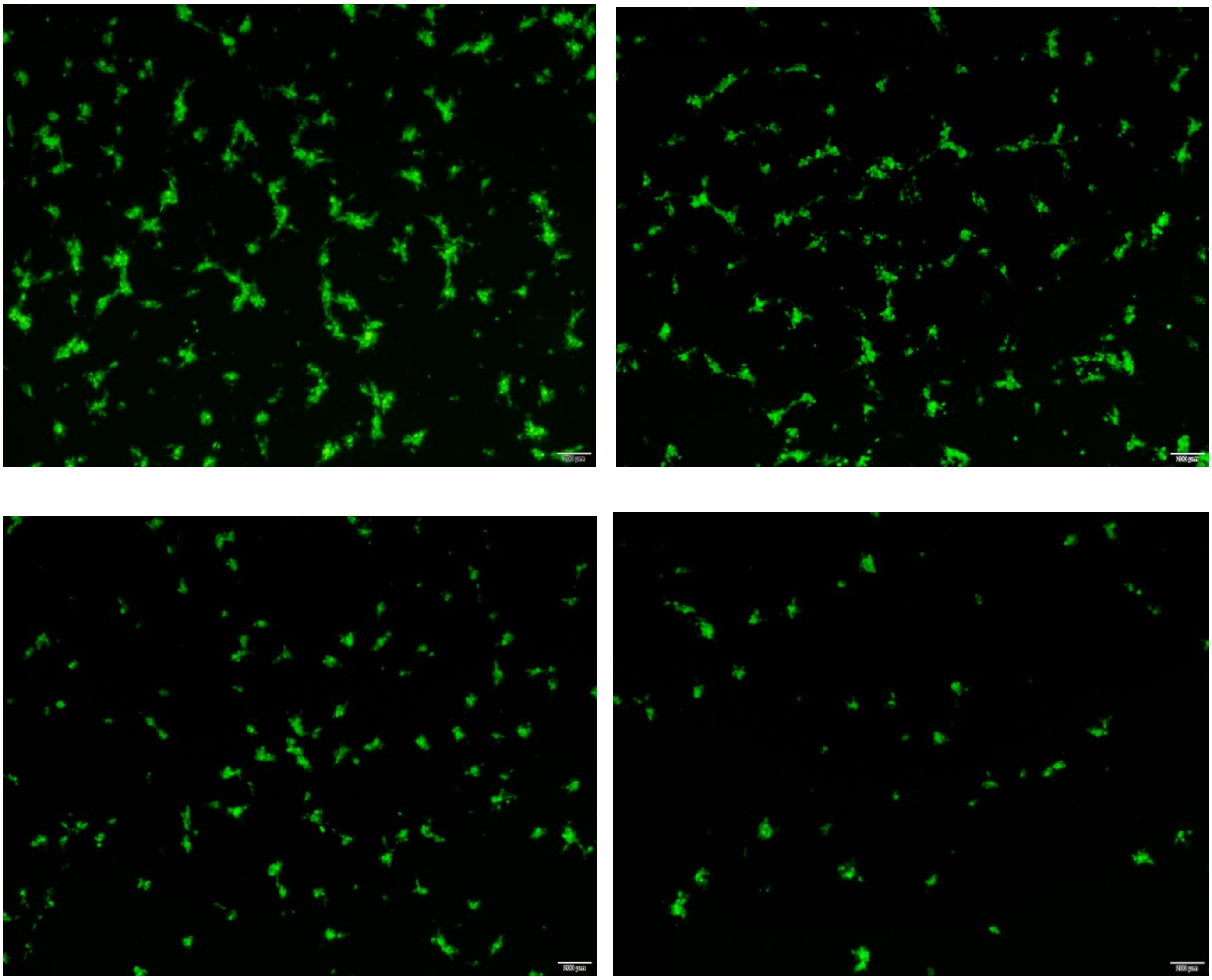

Figure S2A

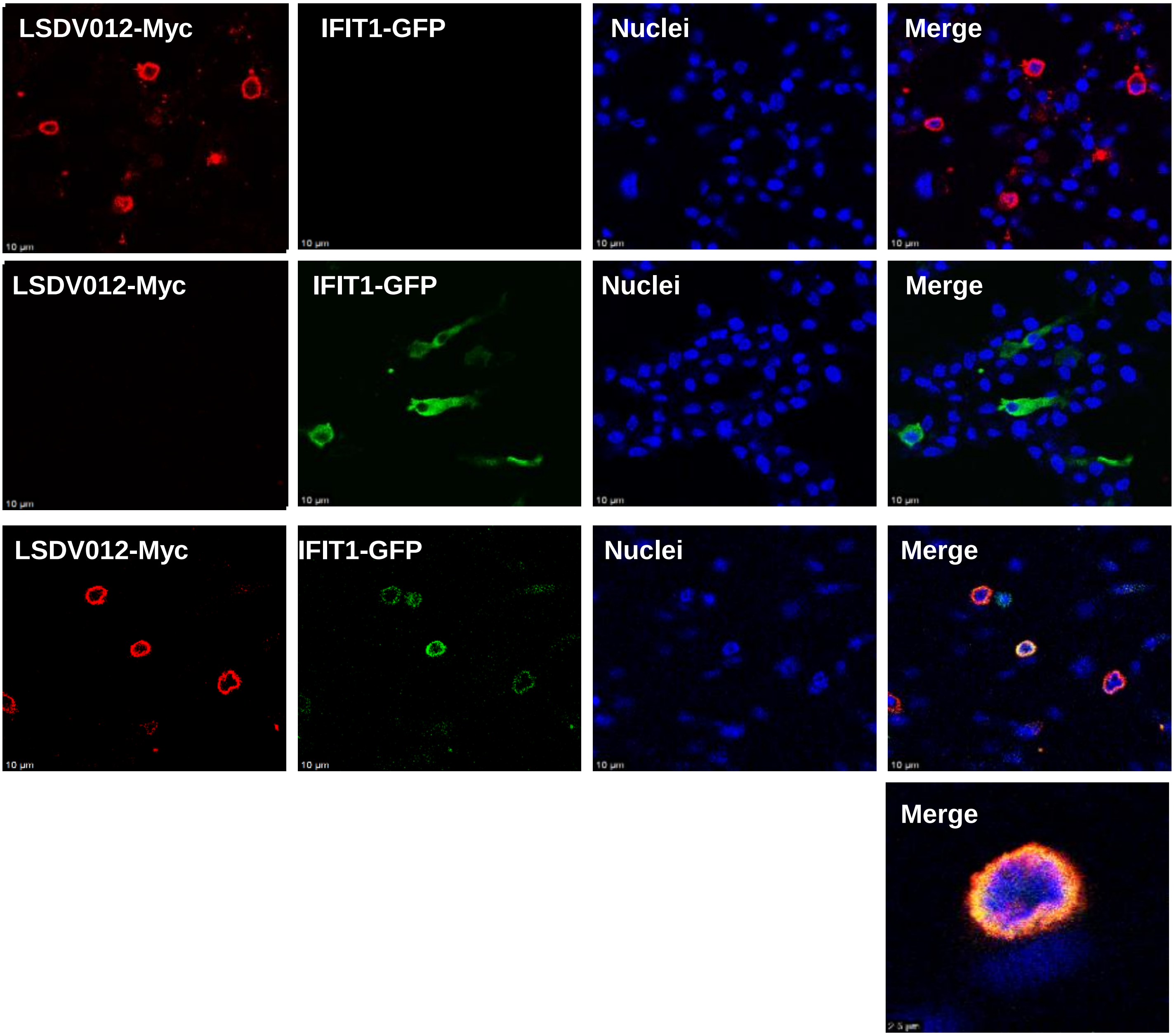
LSDV012-Myc
IFIT1-GFP
Nuclei
Merge
LSDV012-Myc
IFIT1-GFP
Nuclei
Merge
LSDV012-Myc
IFIT1-GFP
Nuclei
Merge
Merge
